# FGF19 in Solid Tumors: Molecular Mechanisms, Metabolic Reprogramming, and Emerging Therapeutic Opportunities

**DOI:** 10.7150/thno.121601

**Published:** 2026-01-01

**Authors:** Jiayi Xu, Peng Sun, Wenjing Zhu, Xinlin Liu, Leina Ma

**Affiliations:** 1 Department of Hepatobiliary and Pancreatic Surgery, The Affiliated Hospital of Qingdao University, Qingdao 266000, China.; 2 Cancer Institute, The Affiliated Hospital of Qingdao University and Qingdao Cancer Institute, Qingdao 266071, China.; 3 Medical Research Department, Qingdao Hospital, University of Health and Rehabilitation Sciences (Qingdao Municipal Hospital), Qingdao 266071, China.

**Keywords:** FGF19, FGFR4, Tumor metabolism, Cancer therapy, Oncogenes

## Abstract

Fibroblast growth factor 19 (FGF19), the human orthologue of murine FGF15, is an endocrine FGF that signals through the FGFR4-β-Klotho receptor complex to regulate bile acid synthesis, glucose and lipid metabolism, and thermogenesis. Beyond its physiological role in metabolic homeostasis, aberrant expression of FGF19 has been increasingly implicated in the initiation and progression of solid tumors. Mechanistically, FGF19 drives signaling cascades that sustain proliferation, invasion, and metabolic reprogramming, while also promoting epithelial-mesenchymal transition, angiogenesis, and immunosuppression to facilitate metastasis. These pleiotropic activities highlight FGF19 as a compelling therapeutic target, and several FGFR4-directed inhibitors have entered clinical evaluation. However, challenges remain, including on-target toxicities, limited selectivity and adaptive resistance. In this review, discuss the molecular mechanisms by which FGF19 shapes tumor biology, evaluate the current status of therapeutic strategies targeting the FGF19-FGFR4 axis, and explore future opportunities such as rational drug combinations and metabolic intervention. A deeper understanding of the interplay between FGF19 signaling, the tumor microenvironment and systemic metabolism will be essential to unlock its potential for precision oncology.

## Introduction

The FGF family is a group of proteins involved in various biological processes, including cell growth, differentiation, and tissue repair 1, 2. Initially identified for their ability to stimulate fibroblast proliferation, a wide range of developmental and physiological functions has since been regulated by FGFs 3. The FGF family is composed of at least 22 members in humans and is classified into seven subfamilies based on their sequence homology and function 4. Specific cell surface receptors are bound by these factors, which activate intracellular signaling pathways that influence cell behavior 5. In cancer, crucial roles are also played by the FGF family, leading to the promotion of tumor growth, angiogenesis, and metastasis, as well as contributing to therapy resistance (Table 1) 6.

Human FGF19 is the ortholog of murine FGF15. Although they have low amino acid sequence similarity, they are homologous proteins. FGF19 is distinguished by its endocrine function, as opposed to the typical paracrine mechanisms of other FGFs. It is secreted by the ileum upon nutrient stimulation, regulating bile acid production in the liver through the FGFR4 and β-Klotho pathway 7. Key enzymes are inhibited by it to maintain bile acid balance and prevent toxicity. Glucose and lipid metabolism are affected by FGF19, with increases in insulin sensitivity and assistance in nutrient partitioning 8, 9. Obesity and type 2 diabetes may be treated by targeting FGF19, due to its lower levels in affected individuals 10.

In recent years, FGF19 has gained attention in cancer research due to its role in various cancers, especially hepatocellular carcinoma (HCC). Tumor growth and progression can be driven by FGF19 overexpression in HCC through the FGF19/FGFR4 signaling pathway 11. Poor prognosis and resistance to immune checkpoint therapies are linked to this pathway. To address this, targeted therapies such as fisogatinib (BLU-554) have been developed, specifically inhibiting the FGF19/FGFR4 pathway and showing potential in curbing HCC progression 12. In nasopharyngeal carcinoma (NPC), FGF19 is overexpressed, promoting angiogenesis and tumor progression, making it a potential non-invasive biomarker for early diagnosis and treatment 13. Increased cell proliferation and poor patient outcomes are associated with the overexpression of FGF19 in head and neck squamous cell carcinoma (HNSCC) 14. Furthermore, FGF19 has been implicated in the development of other cancers, including colorectal cancer (CRC), breast cancer (BC), prostate cancer (PCa), ovarian cancer (OC), gastric cancer (GC), pancreatic ductal adenocarcinoma (PDAC), thyroid cancer (TC), cholangiocarcinoma (CCA), and lung squamous cell carcinoma (LUSC), where it contributes to tumor progression and development through various signaling pathways 15-22. Besides, FGF19 interacts with farnesoid X receptor (FXR), a nuclear receptor that is primarily involved in the regulation of bile acid metabolism. FGF19 can act as a downstream target gene of FXR, with its expression regulated by FXR. During the development and progression of tumors, the abnormal expression of FGF19 and FXR or dysregulation of their signaling pathways is closely associated with tumor progression, like HCC and CRC 23. The relationship between them is of great research value in tumor biology. Additionally, tumor metabolism can be synergistically boosted by FGF19's interactions with other oncogenes, underscoring its potential as a target for cancer therapy. In this review, we delve into the biology and physiological functions of FGF19, as well as the relationship between FGF19 and FXR. Subsequently, we explore its roles in various cancers and discuss the therapeutic applications of targeting the FGF19/FGFR4 signaling pathway in cancer treatment. The aim is to provide comprehensive insights into the potential of targeting the FGF19 pathway for cancer therapy, as understanding the role of FGF19 in these cancers is crucial for developing targeted therapies and improving outcomes of cancer patients.

## The Biology of FGF19

### Structure

**Figure 1:**
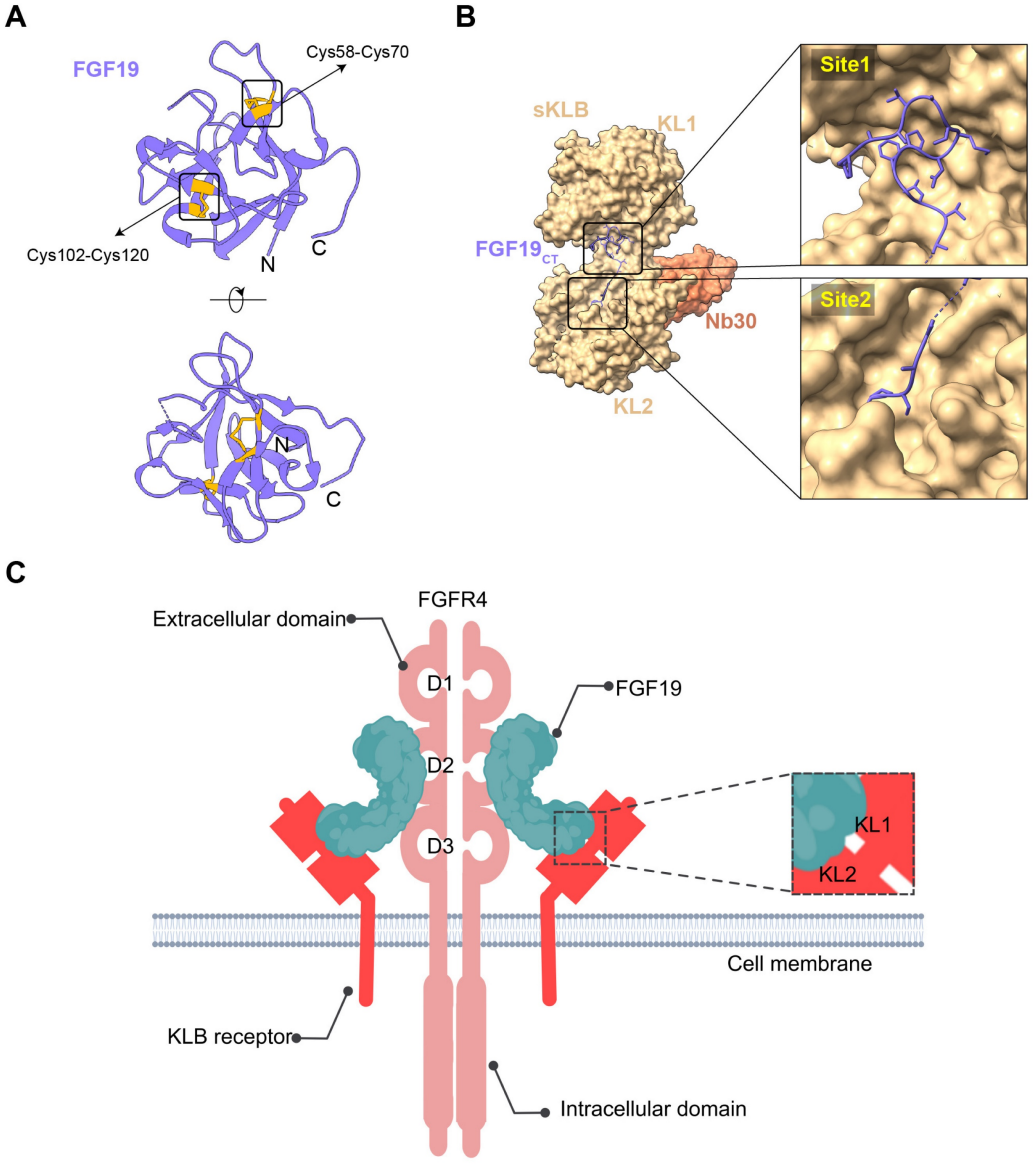
** Structural analysis of FGF19 interactions with β-Klotho and FGFR4.** (A) The front and top view orientations of FGF19 crystal structure (PDB ID: 1PWA). It includes two disulfide bonds between Cys-58 and Cys-70, and between Cys-102 and Cys-120, which contribute to the stabilization of its three-dimensional structure. (B) The structure of beta-klotho in complex with FGF19 c-terminal peptide (FGF19CT) (PDB ID: 6NFJ). It consists of β-Klotho, FGF19, and a nanobody that specifically binds to KLB. The C-terminal region of FGF19 engages with the transmembrane segment of β-Klotho. This interaction involves two principal binding sites. The first site (Site1) features a kinked D-P sequence that binds to KL1, while the second site (Site2) contains an S-P-S sequence and associates with the pseudoglycosidase region within the KL2 domain of β-Klotho. (C) FGFR4 is a single-pass transmembrane tyrosine kinase receptor, comprising an extracellular domain, a transmembrane domain, and an intracellular domain. The extracellular portion of FGFR4 features three immunoglobulin-like domains, namely D1, D2, and D3. The binding interaction primarily occurs via the extracellular region, especially involving the D2 and D3 immunoglobulin-like domains. FGF19 can bind to FGFR4 independently of the co-receptor KLB. Nevertheless, the binding affinity of FGF19 to FGFR4 is significantly enhanced when KLB is present and binds to FGF19. The image was created with MedPeer.cn.

FGF19 is a unique member of the FGF family, marked by differentiating structural features that facilitate its specific biological functions. It is an endocrine FGF with very low heparan sulfate affinity, allowing systemic circulation. Its heparin-binding site diverges from paracrine FGFs. The β10-β12 segment is truncated and lacks the GXXXXGXX(T/S) motif, while the β1-β2 loop is elongated. These structural changes disrupt HS binding, as confirmed by crystallography 24. The β-trefoil configuration constitutes a hallmark feature of FGF19, comprising twelve β-strands arranged antiparallel to form three sets of four-stranded β-sheets. This structural arrangement establishes a stable scaffold essential for receptor interaction 25. Moreover, the β-trefoil architecture is exemplified by its 3D structure determined at a resolution of 1.3 Å. Two recently detected disulfide bonds, located between Cys-58 and Cys-70 as well as between Cys-102 and Cys-120, play a role in sustaining the protein's three-dimensional configuration by anchoring its extended loops 26 (Figure 1A). The heparin interaction sites are characterized by unique shapes and electrostatic properties, accounting for the weak heparin interaction. Distinct structural traits also allow for specific interactions with FGFR4 27. The central region of FGF19, approximately 120 amino acids in length, is characterized by a sequence similarity of 30% to 60% with other FGF proteins 28. Some common functional aspects are maintained with other family members due to this conservation, while unique properties are also exhibited.

Additionally, the C-terminal region of FGF19 plays a crucial role in its biological activity, containing specific sequences that are crucial for interaction with co-receptors and target receptors. The C-terminal of FGF19 interacts with the transmembrane part of β-Klotho. This interaction has two main binding sites. Site1 has a kinked D-P sequence that binds to KL1. A significant role in protein structure stabilization is played by the D-P-L/F sequence in the C-terminal region through intramolecular hydrogen bonding, resulting in a tightly packed and stable conformation critical for binding capabilities 29. Site2 contains an S-P-S sequence and links to the pseudoglycosidase region in β-Klotho's KL2 domain 30 (Figure 1B). This sequence is noted for mimicking sugar molecules, an essential factor in the interaction of FGF19 with KL2 domain, as it emulates the substrate-binding interactions seen in glycoside hydrolase enzymes. Serving as high-affinity cell surface receptors for endocrine FGFs is suggested by the structural similarity of klotho beta (KLB)'s D1 and D2 domains to glycoside hydrolases 31. FGF19 displays activity towards FGFR1c, 2c, 3c, and 4 receptors, but binding is not possible with FGFR1b, 2b, and 3b receptors. Furthermore, its unique structural features are responsible for its function as an endocrine factor, enabling it to travel through the bloodstream to distant targets such as the liver. Signaling cascades, crucial for its biological functions in tumors, are initiated by its interaction with KLB and FGFR4 in hepatocytes or FGFR1c in extrahepatic tissues 32. These structural characteristics of FGF19 are essential in defining its role within the FGF family, while providing insights into potential therapeutic applications in cancer diseases.

### Secretion and Tissue Distribution

The possession of a signal peptide sequence is typical for proteins secreted from cells. Usually, protein is directed through the classical secretion pathway by this signal peptide, which involves translocation into the endoplasmic reticulum and processing in the Golgi apparatus, ultimately leading to its release from the cell via exocytosis 33. However, this traditional pathway is not followed by FGF19 due to the absence of a standard signal peptide sequence. Alternative mechanisms have been proposed for the secretion of FGF19, including via secretory lysosomes or direct translocation across the cell membrane, thereby bypassing the usual route through the endoplasmic reticulum and Golgi apparatus. The distinct nature of FGF19 among growth factors is underscored by this unusual method of secretion. FGF19 is primarily secreted by the enterocytes in the terminal ileum. Its secretion process is closely related to bile acid metabolism. After food intake, bile acids are released into the intestinal lumen. Subsequently, bile acids enter the enterocytes through the apical sodium-dependent bile acid transporter, where they activate the FXR. The activation of FXR promotes the transcription of the FGF19 gene. The produced FGF19 is then secreted into the intestinal lumen and enters the liver via the portal circulation 34.

In terms of tissue distribution, the liver is considered the primary target organ for FGF19, where it plays crucial roles in regulating bile acid synthesis, lipid metabolism, and other metabolic processes 35. Multiple factors regulate the expression of FGF19. Bile acids induce the transcription of the FGF19 gene by activating FXR, with different bile acid components having varying effects on induction 36. HMGA1 can induce the expression and secretion of FGF19, which is more highly expressed in metastatic PDAC cell lines. Silencing of HMGA1 reduces its secretion 37. In disease states such as primary biliary cholangitis (PBC), FGF19 expression is increased, likely related to bile acid metabolic disorders and hepatocyte injury 34. Diet and nutritional status also indirectly regulate FGF19 expression by affecting bile acid secretion. Beyond the ileum and liver, FGF19 is also expressed, albeit at lower levels, in a variety of other tissues, including the brain, adipose tissue, and pancreas. This broader expression pattern suggests that FGF19 may have additional physiological roles beyond its established functions in bile acid homeostasis and liver metabolism. Insulin secretion by pancreatic β cells may be regulated with contributions from FGF19, which is important for maintaining systemic glucose homeostasis 38. Similarly, in the brain, it is possible that FGF19 is involved in regulating metabolic rate and systemic glycemia, indicating a role in central nervous system metabolism. Studies have shown that FGF19 can improve sevoflurane-induced postoperative cognitive dysfunction through the PGC-1α/BDNF/FNDC5 pathway, thereby significantly enhancing cognitive function. Additionally, FGF19 exhibits anti-neuroinflammatory effects by inhibiting neuroinflammatory factors, such as TNF-α, IL-6, and IL-1β, and by reducing oxidative stress to protect neural cells. Neuroprotective effects are exerted by FGF19 through regulation of neuron excitability and metabolic activity, further safeguarding neural cells 39, 40. The potential impact of FGF19 on multiple physiological processes is highlighted by its diverse tissue distribution and varied functions, indicating that it serves as an important factor in both local and systemic regulation.

### Receptor Activation

FGF19 is uniquely engaged with FGFR4 in a manner that distinguishes it from other FGFRs. FGFR4 is a single-pass transmembrane tyrosine kinase receptor that consists of an extracellular domain, a transmembrane domain, and an intracellular domain. The extracellular region of FGFR4 contains three immunoglobulin-like domains, D1, D2, and D3. The interaction is mediated through the receptor's extracellular region, especially the D2 and D3 immunoglobulin-like domains. These domains of FGFR4 facilitate the formation of a stable ligand-receptor complex and provide the specific binding site for FGF19, facilitating a high-affinity interaction crucial for FGFR4 activation 28. Unlike other FGFs, FGF19's interaction with FGFR4 is not dependent on heparan sulfate, which other FGFs typically require as a co-receptor for receptor binding 41. FGFR4 activation is enabled through this direct interaction, triggering downstream signaling pathways that govern metabolic processes. FGF19's binding to FGFR4 is achieved without the co-receptor KLB, necessary for the binding of FGF19 to FGFR1c, 2c, and 3c 42. However, the binding of FGF19 to FGFR4 can be enhanced by KLB when KLB binds to FGF19 (Figure 1C). The genes encoding β-Klotho and its partners, FGFR1c, FGFR2c, and FGFR3c, exhibit distinct expression patterns in mice and humans. In mice, β-Klotho is predominantly expressed in metabolic tissues like the liver, pancreas, adipose tissue, and testes. FGFR1c shows high expression in adipose tissue, pancreas, and male reproductive organs, including epididymis, seminal vesicles, prostate, and vas deferens 43. FGFR2c is more abundantly expressed in the liver and the male reproductive system. FGFR3c is detected in adipose tissue and skin. In humans, β-Klotho is expressed in the liver, where it forms a receptor complex with FGFR1c to mediate FGF21 signaling. FGFR1c is present in adipose tissue and is co-expressed with β-Klotho. FGFR2c is expressed in the liver and forms a receptor complex with β-Klotho. FGFR3c is expressed in the pancreas, where it is co-expressed with β-Klotho for FGF21 signaling 44.

After FGF19 binds to FGFR4, conformational changes occur in FGFR4, activating its tyrosine kinase activity, leading to dimer formation through autophosphorylation and providing binding sites for downstream signaling molecules. The binding and phosphorylation mechanism of FGF19 with FGFR4 is similar in various types of cancer, including HCC and BC. However, the downstream signaling pathways activated by this mechanism may vary between different tumor types. In HCC, FGF19 binds to FGFR4 and forms a complex with FGF receptor substrate 2 and growth factor receptor-bound protein 2, leading to the activation of the Ras-Raf-ERK1/2 MAPK and PI3K-Akt pathways 45. In BC, the co-expression of FGFR4 and FGF19 has been observed, and their interaction is associated with the expression of phosphorylated AKT 46. This suggests that while the initial binding mechanism is conserved, the specific downstream pathways activated may vary depending on the tumor context. The activation of these pathways by FGF19-FGFR4 signaling has been implicated in various cellular processes and is particularly important in the context of metabolism, disease progression, and cancer development 47.

## Regulation of the Gene Encoding FGF19/15

**Figure 2:**
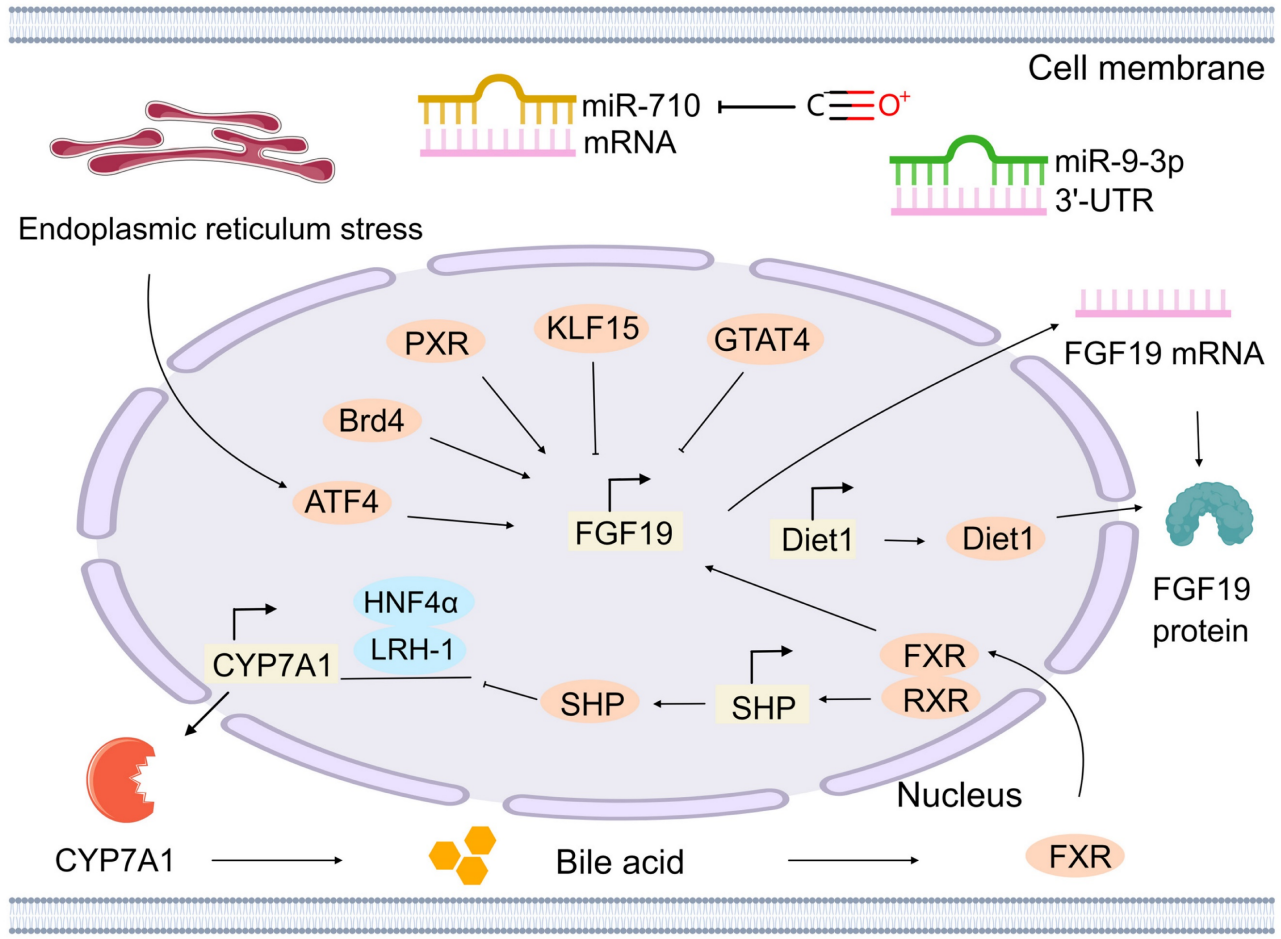
** Regulation of the gene encoding FGF19/15.** At the transcriptional level, bile acids activate FXR, which forms a heterodimer with retinoid X receptor (RXR) to promote FGF19 transcription. This heterodimer also induces SHP, which inhibits CYP7A1 expression mediated by HNF4α and LRH-1, reducing bile acid synthesis and FXR activation, thereby lowering FGF19 expression. Additionally, PXR can upregulate FGF19 expression, while ATF4 under endoplasmic reticulum stress conditions promotes FGF19 expression. Conversely, GATA4 and KLF15 inhibit FGF19 expression. At the transcriptional and post-transcriptional levels, Diet1 overexpression can moderately increase FGF19 mRNA levels and also boost FGF19 protein levels independently of mRNA changes. Certain miRNAs, like miR-710 and miR-9-3p, can transiently suppress FGF19 expression. However, carbon monoxide treatment can reduce the level of miR-710, thereby increasing the expression of FGF19. Moreover, bromodomain-containing protein 4 (Brd4) binds to the FGF19/15 gene promoter, promoting its expression. The image was created with MedPeer.cn.

The FGF19 gene is located on human chromosome 11q13.3 and encodes the FGF19 protein, which is a secretory protein composed of 216 amino acids. The regulation of the FGF19/15 gene is considered a critical factor in understanding its role in cancer, particularly in non-hepatocyte-derived cancers. At the transcriptional level, several transcription factors have been identified as key regulators of FGF19/15 gene expression. The bile acid signaling pathway serves as the primary regulatory pathway. Bile acids in the small intestine activate the FXR, resulting in the formation of a heterodimer with the retinoid X receptor (RXR). This heterodimer recognizes and binds to the FXR response element, thereby promoting transcription of the FGF19 gene 48. The heterodimer also induces expression of the small heterodimer partner (SHP). SHP subsequently inhibits transcription of the cholesterol 7α-hydroxylase (CYP7A1) gene, mediated by hepatocyte nuclear factor 4α (HNF4α) and liver receptor homolog-1 (LRH-1) 49. CYP7A1 is the rate-limiting enzyme in bile acid synthesis, and its activity directly affects bile acid levels. Reduced CYP7A1 activity leads to decreased bile acid synthesis. Consequently, FXR activation is reduced, resulting in decreased FGF19 expression. In addition to FXR, the pregnane X receptor (PXR) can respond to high concentrations of the toxic secondary bile acid lithocholic acid and activate the PXR binding site in the promoter region of the FGF19 gene, thereby promoting the expression of FGF19 50. Other transcription factors, such as Krüppel-like factor 15 (KLF15), GATA binding protein 4 (GATA4), and activating transcription factor 4 (ATF4), are also involved in the transcriptional regulation of the FGF19/15 gene. KLF15 can bind to multiple sites in the FGF19/15 promoter, independently inhibiting FGF19/15 expression outside the bile acid signaling pathway 51. GATA4 directly binds to the GATA4 regulatory element in intron 2 of the FGF19/15 gene and indirectly regulates bile acid uptake and FXR gene expression, thus restricting FGF19/15 expression to the ileum rather than the proximal intestine 52. Under endoplasmic reticulum stress conditions, ATF4 is activated and binds to the amino acid response element in the FGF19/15 promoter, thereby upregulating FGF19/15 expression 53 (Figure 2).

Furthermore, the regulation of FGF19/15 levels by the Diet1 gene occurs at both transcriptional and post-transcriptional levels. In Diet1-deficient mice, a reduction in circulating FGF19/15 levels is observed. Diet1 and FGF19/15 co-localization in intestinal cells suggests their involvement in the FGF19/15 enterohepatic axis. In the intestinal cells of mice and humans, there is a correlation between the expression level of Diet1 and the transcription level of the FGF19 gene. Moderate increases in FGF19 mRNA levels have been demonstrated upon overexpression of Diet1, whereas Diet1 knockdown results in minimal effect 54. In the human HT-29 intestinal cell line, a threefold increase in FGF19 protein secretion has been observed following Diet1 overexpression, whereas partial knockdown reduces secretion by 40%. Moreover, studies utilizing a heterologous promoter to control FGF19 expression have demonstrated that Diet1 can increase FGF19 protein levels independent of its effects on FGF19 mRNA levels 55. In colonic myofibroblasts, FGF19/15 expression is increased by carbon monoxide treatment through the reduction of miR-710 levels, a microRNA that targets FGF19/15 mRNA 56. This finding suggests that miRNAs, such as miR-710, are able to transiently regulate FGF19/15 expression via post-transcriptional mechanisms, particularly under conditions such as oxidative stress. Additionally, the 3′-UTR of FGF19/15 mRNA is specifically recognized by miR-9-3p through complementary pairing with its nucleotide sequence (Figure 2). Following binding, inhibition of FGF19/15 expression can occur through two main mechanisms 57. First, it inhibits translation by preventing translation initiation factors and other components from binding to the mRNA after binding to the 3′-UTR of FGF19/15 mRNA. Second, it degrades mRNA by recruiting RNA degradation-related protein complexes to cleave and degrade FGF19/15 mRNA, thereby inhibiting the production of FGF19/15 protein.

The expression of the FGF19/15 gene is also influenced by epigenetic modifications. Histone modifications, including acetylation, methylation, and other types, are considered crucial mechanisms of epigenetic regulation. In the regulation of the FGF19/15 gene, the promoter region is bound by bromodomain-containing protein 4 (Brd4), a transcriptional regulator that recognizes histone acetylation modifications, thereby promoting its expression. When bromodomain inhibitors, such as JQ-1, are administered, the binding of Brd4 to the FGF19/15 promoter is diminished, thereby resulting in downregulation of FGF19/15 expression 58 (Figure 2).

The regulation of the gene encoding FGF19/15 is a multi-level and multi-factor process, involving transcription factors, post-transcriptional regulation, and epigenetic modifications. Despite these insights, a significant gap in the understanding of the regulation of the FGF19/15 gene in non-hepatocyte-derived cancers remains. Further research is required to explore specific regulatory mechanisms, to identify novel regulatory factors, to investigate the roles of alternative regulatory pathways, and to examine the involvement of different cell types, in order to provide deeper insights into the role of FGF19/15 in non-hepatocyte-derived cancers. Such knowledge will be crucial for the development of targeted therapies and the advancement of our understanding of the complex mechanisms underlying cancer development.

## Functional Overview of FGF19

FGF19 is an endocrine factor with essential roles in regulating various metabolic processes under normal physiological conditions. Here is an overview of its functions in bile acid, glucose, lipid, and energy metabolism.

### Bile Acid Metabolism

**Figure 3:**
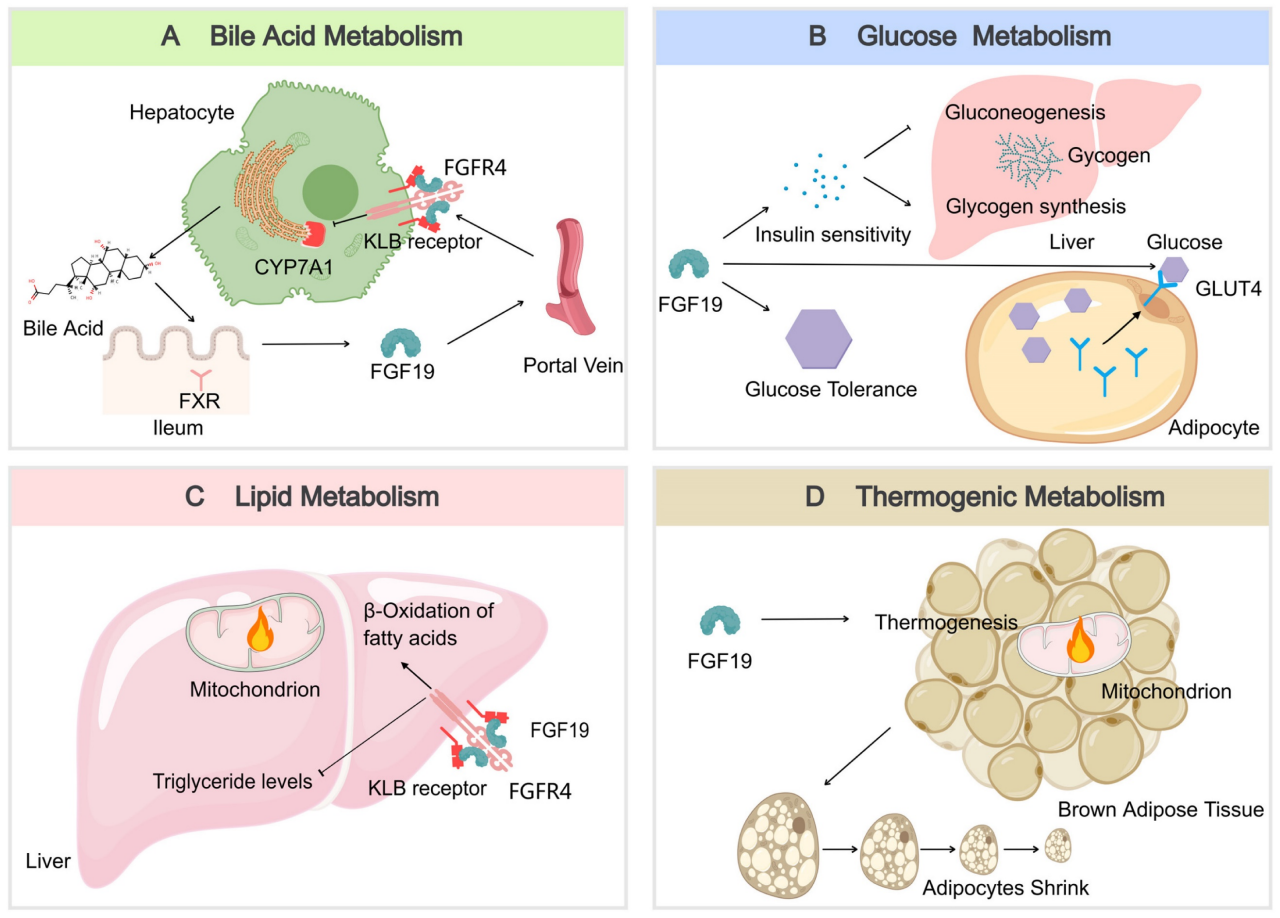
** The multifaceted metabolic regulatory roles of FGF19.** (A) In bile acid metabolism, bile acids trigger FXR activation, leading to increased production of FGF19. FGF19 then travels to the hepatocyte via the portal vein and connects with FGFR4 and β-Klotho. This interaction ultimately prevents cholesterol 7α-hydroxylase (CYP7A1), a key enzyme in the bile acid synthesis pathway, from being transcribed. (B) In glucose metabolism, FGF19 enhances glucose tolerance and insulin sensitivity and is involved in the promotion of glycogen synthesis and the suppression of gluconeogenesis in the liver. Additionally, it enhances GLUT4 translocation in adipocytes, thus facilitating the uptake and utilization of glucose. (C) In lipid metabolism, the FGF19-FGFR4 pathway is associated with a decrease in triglyceride levels and an enhancement of mitochondrial β-oxidation in the liver, potentially impacting overall lipid homeostasis. (D) In thermogenic metabolism, FGF19 activates brown adipose tissue, leading to an increase in energy expenditure and a reduction in adipocyte size. The image was created with MedPeer.cn.

FGF19 serves as a key regulator of bile acid homeostasis. It is produced in the ileum in response to bile acid binding to the nuclear receptor FXR during absorption and then enters the portal circulation 59. Upon reaching the liver, FGF19 binds to the FGFR4/KLB receptor complex, leading to the inhibition of the rate-limiting enzyme CYP7A1. This inhibition reduces the synthesis of new bile acids, thus providing negative feedback to maintain bile acid balance 49 (Figure 3A). This process is essential for preventing excessive bile acid production, which can lead to liver damage and other complications 60.

### Glucose Metabolism

FGF19 also plays a significant role in glucose metabolism. FGF19 is also recognized for its significant role in glucose metabolism. Improvements in glucose tolerance and insulin sensitivity can be mediated through its actions on hepatic and extrahepatic tissues 61. In the liver, gluconeogenesis is inhibited by FGF19, which is the process by which glucose is synthesized from non-carbohydrate precursors, thereby resulting in reduced glucose production. Additionally, glycogen synthesis is stimulated, further contributing to the regulation of glucose levels 62. In extrahepatic tissues, FGF19 can enhance the translocation of GLUT4 in muscle and adipose tissues, thereby facilitating glucose uptake and utilization and contributing to the maintenance of glucose homeostasis 63 (Figure 3B). After meals, FGF19 maintains blood glucose homeostasis by regulating hepatic glycogen synthesis and inducing the dephosphorylation and inactivation of CREB, independent of insulin. In Fgf19 knockout mice (*Fgf19*^-/-^), FGF19 deficiency results in reduced hepatic glycogen, impaired glucose tolerance, and inability to maintain normal blood glucose levels 64.

### Lipid Metabolism

In lipid metabolism, FGF19 is a key regulatory factor that has significant beneficial effects on the lipid profile. FGF19 downregulates the expression of acetyl-CoA carboxylase 2 (ACC2) in the liver, reduces the levels of malonyl-CoA in mitochondria, and increases the activity of carnitine palmitoyltransferase 1, thereby promoting fatty acid β-oxidation and consequently lowering serum triglyceride levels 65 (Figure 3C). After meals, FGF19 can regulate intestinal lipid uptake through the SHP-TFEB axis or the SHP-LSD1 axis, reducing postprandial triglyceride levels 66. Additionally, FGF19 inhibits the expression of intestinal NPC1L1 and postprandial cholesterol absorption by inhibiting SREBF2 via SHP, further regulating lipid metabolism. FGF19 also inhibits hepatic fat production by activating DNMT3A through SHP, a process that is often dysregulated in patients with metabolic-associated fatty liver disease (MAFLD) 67, 68. These mechanisms collectively enable FGF19 to play an essential role in preventing MAFLD, as excessive accumulation of lipids in the liver can lead to inflammation and injury.

### Thermogenic Metabolism

The regulation of energy expenditure and body weight is influenced by FGF19. Numerous studies have demonstrated that an increase in energy expenditure and a reduction in fat mass can be attributed to FGF19, owing to its effects on enhancing brown adipose tissue activity 69. The stimulation of brown adipose tissue and the transformation of white adipose tissue into a more "brown-like" state are linked to higher energy expenditure and a reduced risk of obesity and its associated metabolic disorders 70. Energy expenditure may be enhanced by FGF19, resulting in smaller adipocytes (Figure 3D). These changes are contributing factors to the regulation of body weight and energy balance.

## Omics-based Analysis of FGF19 In Tumors

The extensive metabolic functions of FGF19 are crucial for physiological homeostasis; however, disruptions in these pathways can drive the development of cancer. FGF19's involvement in metabolism makes it a key factor in tumor biology. Omics-based technologies provide new avenues for studying the intricate relationships between FGF19 signaling, metabolism, and tumorigenesis, thereby facilitating the identification of potential therapeutic targets.

Metabolomics has illuminated the metabolic landscape of HCC through a study using capillary electrophoresis-mass spectrometry to analyze CD166-cells from the HCC cell line Li-7 cultured in mTeSR1 medium for 1, 4, and 7 weeks. In this study, 144 metabolites were identified, and significant differences in metabolic profiles across the different culture durations were revealed. Key metabolites, such as L-aspartate, GABA, and L-glutamine, were associated with pathways involved in alanine, aspartate, and glutamate metabolism, which are implicated in the maintenance of cancer stem cell (CSC)-like properties 71. These metabolic changes are associated with FGF19 expression and tumorigenicity, though the precise mechanisms remain unclear. This finding underscores the necessity to explore how FGF19 signaling intersects with metabolic reprogramming to impact tumor progression and identify potential therapeutic targets. In addition, Vittoria Massafra and colleagues conducted a temporal quantitative proteomic analysis in the mouse model to identify FGF19 targets related to metabolism and proliferation. The researchers found that after FGF19 treatment, 189 proteins were upregulated (≥1.5-fold) and 73 proteins were downregulated (≤-1.5-fold). The expression of proteins involved in fatty acid synthesis, including Fabp5, Scd1, and Acsl3, was decreased by FGF19, whereas the expression of Acox1, which is involved in fatty acid oxidation, was increased. Additionally, the expression of proteins known to drive proliferation, including Tgfbi, Vcam1, Anxa2, and Hdlbp, was upregulated by FGF19. Significantly, many FGF19 targets have dual functions in both metabolism and cell proliferation 72. It indicates a close linkage between the roles of FGF19 in metabolism and proliferation. Although FGF19 plays a critical role in metabolic homeostasis within the liver and holds promise for the treatment of metabolic syndrome and cholestatic diseases, its application is limited due to its induction of cell proliferation and the development of HCC, which challenges the development of FGF19 variants that can fully separate metabolic benefits from mitogenic potential.

Building on these metabolic insights and proteomic analysis, genomic studies have provided further evidence of FGF19's role in cancer. It has been shown that the FGF19 gene is frequently amplified in HCC (up to 15%) and has a strong correlation with mRNA expression levels. The FGF19 and CCND1 genes, which are separated by only 45 kb, have often been found to be co-amplified 73. Notably, FGF19 overexpression is tissue-specific to HCC, unlike in other cancers such as breast, lung, or melanoma. In LUSC, FGF19 amplification was found in 40% of smokers but only 5% of never-smokers, suggesting a link between smoking-induced genetic alterations and tumorigenesis 74. Furthermore, *in vitro* experiments have demonstrated that exogenous FGF19 promotes the proliferation of LUSC cells. Future research should focus on elucidating the mechanisms underlying FGF19 amplification and its involvement in tumorigenesis, particularly among smokers, in order to inform the development of targeted therapies.

Moreover, meta-transcriptomic analyses have expanded our understanding of the clinical significance of FGF19 in CRC. Meta-transcriptomic analyses indicate that FGF19 overexpression is a potential marker for CRC and correlates with poor clinical outcomes such as advanced disease and reduced survival rates 75. However, the reason why FGF19 overexpression is associated with poor outcomes remains speculative. Further studies are required to elucidate the molecular pathways involved and to investigate FGF19 as a potential therapeutic target in CRC. The identification of genetic and epigenetic alterations that drive FGF19 overexpression may facilitate the development of targeted therapies and ultimately improve patient outcomes.

The interplay between FGF19, metabolism, and genomic alterations in various cancers is a promising area of research. Future research should employ multi-omics approaches to comprehensively elucidate the role of FGF19 in tumor biology and translate these insights into effective therapeutic strategies.

## The Interplay Between FXR, FGF19, and Tumorigenesis

During the process of tumorigenesis, a close connection exists between FXR, FGF19, and tumors. FGF19 is crucial in bile acid metabolism and enterohepatic circulation, and by inhibiting CYP7A1 expression via FXR, it assists in maintaining the balance of bile acids between the gut and liver. In the liver, FXR acts as a tumor suppressor, primarily functioning through the regulation of bile acid homeostasis. Once the FXR function is lost, the imbalance of bile acid homeostasis may create conditions conducive to the development of HCC 76. FGF19 is a downstream factor of FXR. When the FXR-FGF19 axis is disrupted, the progression of liver cancer can be accelerated. Overexpression of FGF19 has frequently been observed in specific cases of HCC, which are often associated with impaired FXR signaling. In research on the impact of FXR on hepatocarcinogenesis in mice, FXR knockout mouse models have been widely used to elucidate the strong correlation between FXR deficiency and spontaneous liver cancer 77. These investigations have demonstrated that FXR knockout mice spontaneously develop hepatocellular adenomas and HCC by the age of 13 to 15 months. Further mechanistic studies have revealed that the serum and hepatic bile acid levels are significantly elevated in FXR-deficient mice compared to wild-type mice, suggesting that disturbances in bile acid metabolism constitute an important factor in liver cancer development. Additionally, it has been observed that the levels of FGF15 are reduced in FXR-deficient mice, and this reduction is closely linked to dysregulation of bile acid synthesis 78. Notably, restoring FGF15 levels or activating FXR can significantly reduce bile acid accumulation, thereby lowering the incidence of liver cancer. Another study further confirmed the crucial role of bile acid metabolic disorder in hepatocarcinogenesis induced by FXR deficiency, finding that the incidence of liver cancer in FXR-deficient mice increases under a high-bile-acid diet 79. Collectively, these findings underscore the crucial role of FXR in maintaining bile acid homeostasis and inhibiting liver cancer development, thereby establishing a solid theoretical basis for future strategies targeting the prevention and treatment of liver cancer through FXR modulation. Moreover, in FGF19 transgenic mice, spontaneous development of HCC is observed at 8 to 10 months of age. 2- to 4-month-old transgenic mice exhibit significantly higher levels of 5-bromo-2′-deoxyuridine (BrdU)-labeled hepatocytes compared to age-matched wild-type mice 80. It suggests that FGF19 exhibits tumor-promoting characteristics under certain circumstances, particularly in mouse models, where prolonged high-level expression might increase the risk of HCC. In the intestine, FXR signaling is equally crucial for the maintenance of normal intestinal function and the prevention of tumorigenesis. Loss of FXR function in the intestine can also result in increased bile acid levels and dysbiosis of the gut microbiota, thereby contributing to the development of CRC. It is known that bile acid imbalance, which is affected by FXR signaling, can promote intestinal epithelial cell proliferation and mutation, thereby increasing cancer risk 81.

Although both FGF15 and FGF19 inhibit bile acid synthesis, they show significant differences in metabolism and tumorigenesis. FGF19 can reduce HbA1c, protect β-cells, and induce HCC in mice, while FGF15 lacks these effects and does not induce HCC in various mouse models of metabolic diseases. This indicates that fundamental species-related differences exist between FGF15 and FGF19 in mice, thereby limiting the relevance of mouse models in FXR/FGF19 pathway research 82. Hence, safety assessments conducted in mouse models may not adequately reflect human risk, indicating that particular attention should be given to the influence of species differences on tumor risk when translating therapeutic strategies from animal models to clinical settings.

Additionally, FXR agonists are classified into two categories: endogenous bile acids and synthetic non-bile acid compounds. Bile acid agonists are based on a four-ring steroidal framework. For example, 6α-ethyl-chenodeoxycholic acid stabilizes the FXR structure through hydrogen bonds and π-cation interactions. Non-bile acid agonists form stable complexes with the ligand-binding domain of FXR via hydrogen bonds and hydrophobic interactions. Derivatives, such as 14cc of WAY-362450, have also demonstrated high efficacy 83. FXR agonists, such as obeticholic acid (OCA), have been approved for the treatment of chronic liver diseases, including PBC and NASH 84, 85. However, whether FXR agonists increase the risk of cancer varies with the type of cancer. For example, in HCC, OCA has shown anti-tumor activity by reducing tumor and liver weights, as well as inhibiting the mTOR-S6K pathway 86. In triple-negative breast cancer models, OCA also reduced tumor progression by inhibiting cancer cell proliferation and migration, as well as inducing cell death 87. Conversely, GW4064, an FXR agonist, has been reported to enhance the migration and invasion of PanCa cells 88. Given the dual roles of FXR agonists in cancer treatment, future research should focus on developing personalized therapeutic strategies that consider the specific FXR status present in various cancer types. Additionally, investigating the mechanisms underlying FXR's impact on cancer cell behavior and identifying biomarkers that predict a response to FXR modulators may improve the efficacy and safety profiles of these treatments. This approach may help optimize the use of FXR-targeted therapies, thereby minimizing adverse effects and maximizing anti-tumor benefits across diverse cancers.

The interaction between intestinal FXR agonists, FXR antagonists, and the gut microbiota has garnered attention regarding their roles in tumor development and progression, with recent studies providing novel insights and potential therapeutic strategies. In gastroesophageal adenocarcinoma (GEAC), high-fat diets can alter the gut microbiota, increasing bile acid levels and promoting carcinogenesis through inflammation and DNA damage. FXR can regulate bile acid homeostasis and inhibit cancer-related processes; its loss accelerates GEAC, while FXR agonists, such as OCA, can ameliorate dysplasia 89. FGF19 signaling, closely linked to FXR activation, may protect against cancer by modulating bile acid metabolism and reducing inflammation. Conversely, deoxycholic acid (DCA), a FXR antagonist that is produced by microbial metabolism, can inhibit FXR signaling. This inhibition reduces FGF19 expression, disrupting the FGFR4/β-Klotho complex activation and leading to increased bile acid synthesis, which can promote CRC development 81. In HCC, FXR activation induces FGF19 expression, which is vital for liver homeostasis and can counteract HCC development. Conversely, DCA reduces FGF19 expression and may facilitate HCC progression by promoting DNA damage and enhancing cell survival 90, 91. However, the potential role of the gut microbiota, particularly bacterial species that metabolize bile acids to produce FXR agonists and antagonists, in influencing cancer through FXR signaling and FGF19/15 induction remains largely unexplored in many other tumors. Looking ahead, this area holds significant potential for future research. The ability of the gut microbiota to modulate bile acid metabolism, thereby affecting FXR and subsequently FGF19, could have significant implications for the development and progression of FGF19-related cancers. In-depth investigation of these interactions may not only improve understanding of the gut-cancer axis, but also facilitate the development of novel therapeutic strategies targeting both the microbiota and FXR signaling pathways in cancer treatment.

## Role of FGF19 in Cancer Cachexia

Cachexia is a multifactorial syndrome, typically caused by cancer or chronic diseases, and is characterized by muscle wasting, weight loss, and fatigue. It stems from metabolic and inflammatory issues. Clinically, the diagnosis is established through the observation of weight loss and reduced serum albumin levels. Although current management strategies, including nutritional support, pharmacological interventions, and exercise regimens, are employed, these treatments remain insufficient, and the syndrome continues to contribute to diminished quality of life and reduced survival, accounting for approximately 20% of cancer-related mortalities 92.

In the state of cancer cachexia, metabolic dysfunction represents a hallmark, with FGF19 hypothesized to play a role in its pathogenesis by influencing metabolic pathways. FGF19 has been shown to activate the AMPK/SIRT-1/PGC-α signaling pathway, which promotes the hypertrophy of muscle fibers and thereby increases muscle mass in healthy mice 93. In the meantime, muscle wasting is a typical manifestation of cancer cachexia. Thus, it can be inferred that impaired FGF19 function may contribute to disruptions in muscle metabolism, adversely affecting muscle quality and function. Furthermore, cancer cachexia is strongly associated with systemic inflammatory responses. FGF19 can act through FGFR4 to modulate the Wnt/GSK-3β/β-catenin signaling pathway, thereby sustaining hyperproliferation of keratinocytes and perpetuating inflammatory reactions as observed in psoriasis 94. Elevated levels of inflammatory mediators are commonly observed in cancer cachexia, and it is plausible that FGF19 exacerbates the inflammatory milieu, thus advancing disease progression. Alterations in the gut microbiota have also been implicated in the pathophysiology of cancer cachexia 95. FGF19 is involved in bile acid metabolism, which is regulated by the gut microbiota; thus, FGF19 may indirectly modulate the composition and function of gut microbiota via its effects on bile acid metabolism, ultimately contributing to the development of cachexia.

Given the critical role of FGF19 in metabolic regulation, modulation of FGF19 expression or activity could potentially ameliorate metabolic disturbances and improve the nutritional status of patients with cancer cachexia. Moreover, owing to its regulatory effects on muscle mass, therapeutic targeting of FGF19 may also offer benefits in the management of muscle wasting in these patients.

## Role of FGF19 in Tumor Metabolism

The preceding section explored the omics analysis of relevant studies, which revealed the intricate landscape of FGF19 in tumors, elucidated the complex interplay between FXR, FGF19, and tumorigenesis, and examined its association with cachexia. However, tumor metabolic reprogramming, a key characteristic that enables tumor cells to adapt to their microenvironment and sustain growth and proliferation, is considered of considerable significance. FGF19, acting as a core regulator of cellular metabolism, may coordinate the alterations in tumor cell metabolic pathways, thereby promoting their rapid growth and survival. In this section, we aim to dissect the potential mechanisms underlying FGF19-driven tumor metabolic reprogramming. A thorough understanding of these mechanisms is anticipated to reveal the oncogenic potential mediated by FGF19 via tumor metabolism and to offer new directions for the development of feasible therapeutic strategies.

### The Role of FGF19 in Glycolysis (Warburg Effect) Within Tumor Metabolism

Tumor cells exhibit increased glycolytic flux, favoring aerobic glycolysis even in oxygen-rich conditions. This phenomenon is referred to as the Warburg effect 96. The overexpression of glucose transporters such as GLUT1 and GLUT3 serves as a key factor in the Warburg effect. GLUT1, which is regulated through pathways such as PI3K/AKT, HIF-1, p53, Ras, and c-Myc, serves as the primary glucose transporter in many cancers 97. Activation of the PI3K/AKT/mTOR pathway can be caused by KRAS mutations in lung adenocarcinoma, which induces GLUT1 expression 98, 99. Under hypoxic conditions, the direct regulation of GLUT3 transcription is performed by HIF-1α 100. The subcellular localization of GLUT proteins on the cell membrane is crucial for their function, and glucose uptake is enhanced by tumor cells through the regulation of GLUT expression and membrane localization 101. FGF19 differentially affects GLUT1 and GLUT3. Under normal diets, placental GLUT1 is upregulated by FGF19 to enhance glucose transport; however, this effect is reduced in high-fat diets due to already elevated GLUT1 expression driven by high glucose levels. In contrast, minimal impact is observed by FGF19 on GLUT3 expression, as it is primarily regulated by insulin and linked to placental insulin sensitivity 63.

The interplay between FGF19, the PI3K/AKT signaling pathway, and GLUT1 is extremely crucial in tumor metabolism and significant in cancer progression and treatment. The PI3K/AKT signaling pathway, which is vital for the proliferation and survival of tumor cells, can be activated by FGF19. The regulation of GLUT1 by the PI3K/AKT pathway occurs through multiple mechanisms 102. Specifically, glucose uptake is enhanced by the PI3K/AKT pathway, which promotes the translocation of GLUT1 to the cell membrane and upregulates its expression through mechanisms involving the mechanistic target of rapamycin complex 1 (mTORC1) and HIF-1α 103, 104. This increased glucose uptake provides energy and metabolic intermediates to cells, supporting rapid proliferation. In cancer, high GLUT1 expression in cancer cells is often associated with the abnormal activation of the PI3K/AKT pathway. This high expression contributes to the Warburg effect, in which tumor cells obtain energy preferentially through glycolysis rather than oxidative phosphorylation to meet the metabolic demands of rapid proliferation, thereby promoting tumor growth, metastasis, and chemoresistance 105. Lenvatinib serves as a targeted therapeutic drug that inhibits PI3K/AKT pathway activity through the downregulation of FGF19. This inhibition effectively suppresses tumor cell proliferation, migration, and invasion, and apoptosis is induced. It has been found in studies that the anti-tumor effects of lenvatinib are enhanced by FGF19 depletion, through the reduction of p-PI3K and p-AKT expression. Conversely, the inhibitory effects of lenvatinib on the PI3K/AKT pathway are weakened by FGF19 overexpression, thereby reducing its anti-tumor efficacy 15. Therefore, FGF19, through the modulation of the PI3K/AKT pathway and the indirect influence on GLUT1-mediated glucose metabolism, plays a key role in tumor metabolism and represents a promising target for cancer metabolism therapy.

Tumor metabolism is significantly impacted by the Wnt/β-catenin signaling pathway, which promotes the Warburg effect and enhances glycolysis over oxidative phosphorylation. Achieving this involves the upregulation of pyruvate dehydrogenase kinase 1 (PDK1), which inhibits the activity of pyruvate dehydrogenase (PDH), thereby reducing pyruvate entry into mitochondria and increasing lactate production 106. Expression of lactate/pyruvate transporters such as MCT1/SLC11A1 is also increased, facilitating lactate efflux and maintaining acid-base balance 107. Additionally, the regulation of amino acid metabolism is performed by the pathway through the inhibition of transcription factors like CEBPA and FOXA1 108. In HCC, the downregulation of enzymes involved in histidine and arginine metabolism occurs, altering the cellular amino acid balance, inhibiting the urea cycle, and promoting tumor cell proliferation. Tetrahydrofolate levels can also be increased, reducing sensitivity to methotrexate 109. Metabolic reprogramming is promoted by the interaction between the Wnt/β-catenin pathway and c-Myc, thereby enhancing glycolysis and amino acid metabolism through gene regulation 110. Furthermore, mitochondrial function can be impaired through the inhibition of cytochrome c oxidase, thus further driving glycolysis for energy production 111.

In glioma cells, the regulatory role of FGF19 within the Wnt/β-catenin pathway is particularly evident. Increased levels of FGF19 are observed with the downregulation of miR-520e, which leads to the stabilization of β-catenin and the activation of the Wnt/β-catenin pathway, driving glioma cell proliferation and invasion. Conversely, FGF19 levels are reduced by the overexpression of miR-520e, thereby lowering β-catenin levels and activity. However, these antitumor effects can be partially reversed by restoring FGF19 expression, which reactivates the Wnt/β-catenin pathway 112. This underscores the critical role of FGF19 in maintaining the activity of the Wnt/β-catenin pathway and downstream metabolic effects. Given the central role of FGF19 in regulating the Wnt/β-catenin pathway and its downstream metabolic effects, the targeting of FGF19 represents a promising therapeutic strategy for modulating tumor metabolism. The inhibition of FGF19 may disrupt the activation of the Wnt/β-catenin pathway, thereby reversing the Warburg effect and potentially overcoming chemotherapy resistance.

FGF19 may also impact tumor metabolism through the activation of AMPK, a key sensor of cellular energy status. The binding of FGF19 to its receptor can trigger the activation of AMPK, leading to a series of downstream effects. This activation may enhance mitochondrial biogenesis and function via the AMPKα-PGC-1α-SIRT1 pathway, promoting oxidative phosphorylation and increasing ATP production 113. Hepatic stellate cells can also be activated by FGF19, leading to the release of ANGPTL4. The released ANGPTL4 enhances glycolysis in HCC cells, thus promoting the progression of HCC 114. Hence, the balance between glycolysis and oxidative phosphorylation in tumor cells might be influenced by FGF19, potentially shifting metabolic preference toward more efficient energy production. Significant implications for tumor growth, survival, and therapeutic strategies targeting tumor metabolism could result from this. These findings highlight FGF19's potential as a novel therapeutic target in the development of tumor metabolism-targeted therapeutic strategies.

### The Role of FGF19 in Fatty Acid Synthesis Within Tumor Metabolism

Tumor cells exhibit a remarkable reprogramming of metabolic pathways to support rapid proliferation, survival, and aggressive behavior. Among these metabolic adaptations, an enhanced capacity for fatty acid synthesis is recognized as a critical feature. This elevated synthetic pathway is essential for meeting the heightened demands for membrane components and signaling molecules that drive the relentless expansion and invasive nature of tumors 115. The enhanced synthetic capacity in tumor cells relies on the upregulation of key enzymatic components. The conversion of acetyl-CoA to malonyl-CoA, the committed step in fatty acid synthesis, is catalyzed by Acetyl-CoA carboxylase (ACC) 116. Long-chain fatty acids are produced by Fatty acid synthase (FASN) using malonyl-CoA. SREBP-1, a transcription factor, drives the expression of both ACC and FASN. SREBP-1 is stabilized by the PI3K/AKT pathway, often active in tumors due to mutations, leading to its nuclear translocation, where it enhances the transcription of ACC and FASN 117, 118. FASN is frequently overexpressed in various cancers. Promising results have been shown by inhibitors like TVB-2640, which target FASN by disrupting fatty acid elongation to reduce membrane lipid synthesis and impair tumor growth 119. Lipids are also acquired by tumor cells from their microenvironment. The multifaceted regulation of fatty acid synthesis in tumor cells is a critical metabolic vulnerability 120. Targeting these pathways holds significant therapeutic potential, with research exploring combination therapies to disrupt the lipid metabolic network sustaining tumor progression.

In tumor cells, it has been demonstrated that FGF19 can activate downstream signaling pathways, including the FGFR4-ERK pathway, to promote the expression of key enzymes involved in fatty acid synthesis, such as FASN and ACC. An increase in these enzymes accelerates the *de novo* synthesis of fatty acids within tumor cells, providing essential membrane components and energy for the rapid proliferation of tumors. Ras proteins within the cell are recruited and activated by FGFR4, and the activated Ras proteins further activate Raf kinase. MEK is phosphorylated and activated by Raf kinase, which subsequently phosphorylates ERK to activate it. The activated ERK translocates from the cytoplasm to the nucleus, where it phosphorylates various transcription factors, such as c-Fos, c-Jun, Elk-1, and other ETS family members, altering their activity states 121. The phosphorylated transcription factors bind to specific sequences within the promoter regions of the FASN and ACC genes, such as AP-1 sites, thereby enhancing their transcriptional activity. Targeting FGF19 holds therapeutic potential for modulating tumor metabolism by inhibiting the FGFR4-ERK pathway, thereby reducing the expression of key fatty acid synthesis enzymes, such as FASN and ACC, and ultimately disrupting the metabolic support for tumor proliferation.

### The Role of FGF19 in Amino Acid Metabolism Within Tumor Metabolism

Amino acids are fundamental components of proteins and indispensable for various cellular processes, including cell growth, proliferation, and metabolism. In the context of tumor metabolism, amino acids serve multiple critical functions. Firstly, they are the primary substrates for protein synthesis, a process that is vital for the rapid proliferation of tumor cells. An increased demand for amino acids is often observed in tumor cells to support their elevated rate of protein synthesis and biomass accumulation. In addition to their role in protein synthesis, amino acids function as signaling molecules, regulating metabolic pathways that support rapid tumor growth 122. The mTORC1 pathway is one of the key pathways regulated by amino acids. mTORC1 is a central regulator of cell growth, proliferation, and metabolism, and integrates signals derived from nutrients, growth factors, and cellular energy status. Amino acids, particularly branched-chain amino acids such as leucine, are considered potent activators of mTORC1. The activation of mTORC1 by amino acids results in the promotion of protein synthesis, lipid synthesis, and glucose uptake, and the inhibition of autophagy, thereby supporting the anabolic processes necessary for tumor growth 123. Moreover, amino acids are also involved in the regulation of other metabolic pathways in tumor cells. For example, glutamine, one of the most abundant amino acids in the bloodstream, is a major source of nitrogen and carbon for tumor cells. Metabolism via the tricarboxylic acid (TCA) cycle enables glutamine to provide both energy and biosynthetic precursors 124. Tumor cells often exhibit increased glutamine uptake and metabolism, a phenomenon known as "glutamine addiction," which is essential for their survival and growth 125. Additionally, serine and glycine are important for nucleotide synthesis and one-carbon metabolism, processes that are crucial for DNA synthesis and repair in rapidly dividing tumor cells.

FGF19 is a crucial metabolic regulator that not only plays a role in bile acid and lipid metabolism but also significantly impacts amino acid metabolism. In tumor metabolism, the regulation of amino acid metabolism by FGF19 through multiple mechanisms has been shown to influence tumor cell growth and proliferation. FGF19 can activate the mTORC1 signaling pathway. Through the activation of downstream MAPK and PI3K/AKT pathways via its receptor FGFR4, mTORC1 activity is enhanced, thereby promoting amino acid uptake and utilization, processes that are essential for rapid tumor cell proliferation 126. Additionally, FGF19 can modulate the expression of enzymes involved in amino acid metabolism. For example, an increase in the expression of glutamine synthetase via the mTORC1 pathway has been observed, thus influencing the intracellular amino acid balance. In tumor models, increased cell proliferation and tumor development have been closely associated with FGF19 overexpression; for example, in HCC models, significant upregulation of proliferation markers such as Ki-67 by FGF19 has been demonstrated 127. This effect is mediated in part by the regulation of amino acid metabolism, as amino acids are essential for the synthesis of proteins and nucleic acids necessary for rapid cell division.

Given its key role in amino acid metabolism and tumor development, the FGF19 signaling pathway, particularly FGFR4, has been regarded as a potential therapeutic target. A modified FGF19 variant (M70), which retains metabolic regulatory activity but lacks tumorigenic potential, has been identified. M70, characterized by a five-amino acid deletion and three substitutions at the N-terminal region, has been demonstrated to fail to induce liver tumors in mice when expressed via an adenoviral vector over a 24-week period. Notably, this approach has been shown to yield serum levels of FGF19 or M70 at approximately 2 µg/mL, a concentration 10,000 times higher than typical human serum levels. Within this model, a significant increase in the expression of several markers, including glutamine synthase, Ki-67, α-fetoprotein, and cyclins by FGF19 has been observed, whereas M70 has not been found to elicit these effects 128. This variant may offer therapeutic benefits by modulating amino acid metabolism while minimizing the associated risk of tumor progression.

### FGF19-mediated Regulation of Mitochondrial Function

FGF19 exerts significant regulatory effects on mitochondrial function. It can promote mitochondrial biogenesis and fusion by activating the FGFR4/AMPKα-p38/MAPK signaling axis. As a result of this activation, the expression of mitochondrial fusion proteins MFN1 and MFN2 is upregulated, whereas Drp1 phosphorylation (Ser616)-mediated mitochondrial fission is inhibited 113. These processes contribute to the maintenance of cellular energy homeostasis and the enhancement of metabolic as well as antioxidant capacities. Moreover, activation of the AMPK pathway by FGF19 can suppress NADPH oxidase activity and elevate antioxidant enzyme levels, ultimately resulting in a reduction of LPS-induced ROS 60. Furthermore, mitochondrial dysfunction is alleviated through the promotion of PGC1α, mitochondrial transcription factor A, and heme oxygenase 1 expression within mitochondria by FGF19. This mechanism is particularly effective in mitigating mitochondrial dysfunction induced by palmitate 129. Overall, these effects of FGF19 enhance cellular metabolic flexibility and stress resistance through the promotion of mitochondrial biogenesis and fusion, in addition to the alleviation of mitochondrial dysfunction. Its potential role in tumor metabolic reprogramming may contribute to tumor aggressiveness and therapy resistance. Future investigations are warranted to explore the intersection of FGF19-mediated mitochondrial alterations and oncogenic pathways, as such studies may offer novel strategies for targeting tumor metabolism and improving outcomes in cancer therapy.

### The role of FGF19-mTOR Signaling Within Tumor Metabolism

The mTOR signaling pathway, particularly mTORC1, serves as a key regulator of anabolic metabolism in tumor cells. Signals from nutrients and growth factors are integrated by it to drive various biosynthetic processes 130. IRS-1 is suppressed by chronic mTORC1 activation, dampening PI3K/AKT signaling and promoting insulin resistance in cancer cells. Influenced by amino acids via Rag GTPases, glucose through AMPK, and hypoxia via HIF-1α, mTORC1 is activated downstream of RTKs 131. Once active, S6K1 and 4E-BP1 are phosphorylated by mTORC1 to promote protein synthesis, while lipid metabolism is regulated through lipin-1 phosphorylation 132. MYC and HIF-1α are also activated by mTORC1, which leads to the upregulation of amino acid transporters 133. Lipogenesis is promoted by mTORC1 through the stimulation of SREBP1/2 via Lipin-1 phosphorylation, which drives the expression of fatty acid synthase and HMG-CoA reductase 134. However, PGC-1α is inhibited by mTORC1, reducing mitochondrial biogenesis and oxidative phosphorylation, and favoring glycolysis 135. This interplay highlights mTORC1's role in meeting the anabolic needs of tumor cells.

FGF19 binding to FGFR and Klotho activates the mTOR pathway, promoting protein synthesis and tumor cell metabolism. It supports rapid tumor proliferation and survival under increased metabolic demand. The ERK/AKT-p70S6K-S6 pathway is upregulated by the FGF19/FGFR4 axis in HCC and HNSCC. mTORC1 and ERK pathways converge on S6, driving metabolic reprogramming to meet the energy needs of rapidly growing tumor cells 126. In tumors where FGF19 is overexpressed, mTOR activation plays a crucial role in metabolic adaptation. For instance, in LUSC, a correlation exists between high FGF19 expression and mTOR activation, enhancing glucose and amino acid uptake, glycolysis, and oxidative phosphorylation to support tumor growth. Metabolic activity is suppressed by the mTOR inhibitor AZD2014 in FGF19-overexpressing LSQ cells, and tumor growth is inhibited *in vivo*, highlighting the key regulatory role of mTOR in FGF19-driven tumor metabolism 136. Given their roles in tumor metabolism, the combination of therapies targeting both pathways may yield better antitumor effects. Such a combination would inhibit tumor cell proliferation signals and metabolic support, more comprehensively suppressing tumor growth and progression. In summary, the mTOR pathway is activated by FGF19, which drives metabolic reprogramming in tumor cells and supports their growth and proliferation. The inhibition of the mTOR pathway can block this process, providing a basis for combined therapeutic strategies targeting FGF19 and mTOR, offering a new direction for tumor treatment, particularly in targeting tumor metabolism.

### The link between FGF19 and Oncogenes (MYC and KRAS) Related to Tumor Metabolism

Metabolic alterations in cancer cells are crucially prompted by the oncogenes MYC and KRAS. Glutamine catabolism is promoted by MYC through the activation of GLS and IDH2 137. One-carbon metabolism is stimulated by MYC through PHGDH and SHMT2, and it collaborates with transcription factors such as SP1 and E2F to modulate metabolic gene expression. In contrast, glucose uptake is boosted by KRAS through the PI3K/AKT pathway, micropinocytosis is stimulated by RAC1, SREBP1 is stabilized for lipogenesis, and the redox balance is maintained via NOX4 and G6PD 138. The activity of G6PD can be enhanced by KRAS activation through the PI3K/Akt signaling pathway, thereby promoting cell proliferation and metabolism 139. In tumors with KRAS mutations, the expression level of G6PD is closely related to tumor progression and prognosis. For example, in lung cancer patients with co-mutations of KRAS and LKB1, a high expression of G6PD is associated with a poor prognosis. Additionally, the growth and survival of KRAS-driven tumor cells are supported by G6PD through the maintenance of NADPH levels and antioxidant capacity 140. Meanwhile, the expression and activity of NOX4 can also be significantly increased by KRAS activation. In lung cancer cells with KRAS mutations, the expression level of NOX4 is markedly elevated. Cellular signaling and metabolic processes are regulated by NOX4 through the generation of ROS. In KRAS-driven tumor cells, NOX4 activity enables cells to adapt to oxidative stress, thereby promoting tumor progression 141. ULK1 is also activated by NOX4 to induce autophagy, and metabolic flux is adjusted through enzyme modifications such as AMPK-mediated ACLY phosphorylation 142. In liver cancer, MYC overexpression is frequently observed, fueling cell proliferation and metabolism by activating glycolysis, fatty acid synthesis, and amino acid metabolism 143. Elucidating these mechanisms is vital for designing targeted therapies against cancers driven by MYC and KRAS.

Research has shown that FGF19 and its analog, aldafermin, synergize with MYC to induce aggressive hepatocarcinogenesis. In experimental models, liver cancer development is greatly accelerated by the combined overexpression of FGF19 and MYC, resulting in more aggressive tumor phenotypes. This synergy might result from the activation of common signaling pathways or the regulation of metabolism-related genes, further boosting the proliferative and survival abilities of tumor cells and driving the rapid progression and deterioration of liver cancer 69. The mechanism of this synergistic effect remains unclear and requires further research to elucidate the potential of treating liver cancer through FGF19 and MYC. KRAS mutations, however, are one of the primary driving factors in PDAC. It has been shown that HMGA1 is upregulated in KRAS-driven tumors and can directly induce the expression of FGF19. The expression and secretion of FGF19 can be activated by HMGA1, serving as a "molecular switch." FGF19 not only provides signals to induce rapid growth and invasion of tumor cells into surrounding tissues but also collaborates with HMGA1 to form a dense, fibrous, scar-like wall around tumor cells, preventing treatment from reaching them 37. In PDAC, the high expression levels of FGF19 and HMGA1 together define a tumor subtype characterized by an extremely poor prognosis. This suggests that in KRAS-mutant PDAC, tumor progression and stromal formation may be further promoted by FGF19 through its synergistic action with HMGA1. Given the synergistic effects of FGF19 with MYC and KRAS, a therapeutic strategy targeting FGF19, MYC, and KRAS jointly is expected to become a new direction in cancer treatment. A comprehensive understanding of the specific mechanisms of FGF19 in tumor metabolism is essential for developing more effective therapeutic approaches.

## The Other Roles of FGF19 in Tumors

### Promoting Tumor Cell Proliferation

FGF19 exerts a significant impact on promoting tumor cell proliferation primarily through the activation of the FGFR4 signaling pathway. A series of intracellular signal transduction cascades is triggered once FGF19 binds to FGFR4 144. Various biological processes related to cell growth and division involve these activated molecules. For example, the expression of cyclins and cyclin-dependent kinases (CDKs), key regulators of the cell cycle, can be promoted by these molecules 145. FGF19 can engage in crosstalk with the epidermal growth factor receptor (EGFR) signaling pathway. Research has shown that FGF19 can induce the expression of the EGFR ligand amphiregulin, which subsequently activates the β-catenin signaling pathway. This activation leads to the upregulation of cyclin D1, which promotes the cell cycle process and enhances tumor cell proliferation 146. Cyclin D1 activates the CDK4/6 kinase, which phosphorylates and inactivates the RB1 protein, thereby removing the inhibitory effect of RB1 on the cell cycle and facilitating the transition of cells from the G1 phase to the S phase, thus accelerating cell proliferation 147.

### Enhancing Tumor Cell Invasion and Metastasis

FGF19 is integral to the enhancement of tumor cells' invasion and metastasis capabilities. This effect is realized by inducing tumor cells to secrete matrix metalloproteinases (MMPs). Upon interacting with tumor cells, FGF19 activates specific transcription factors, which are subsequently bound to the promoter regions of MMP-encoding genes. This binding promotes the transcription and translation of these genes into MMP proteins. MMPs represent a family of proteolytic enzymes capable of degrading extracellular matrix components such as collagen, laminin, and fibronectin 148. As the extracellular matrix is degraded, the physical barriers that restrict tumor cell movement are dismantled. This process enables tumor cells to detach from the primary tumor mass, invade surrounding tissues, and enter the bloodstream or lymphatic system, thereby facilitating metastasis 149.

In the complex process of cancer progression, the EMT is recognized as a critical event. It is a complex cellular biological process characterized by the loss of cell polarity and intercellular connections in epithelial cells, as well as the acquisition of mesenchymal cell properties. This transition endows tumor cells with enhanced migratory and invasive capabilities, thereby facilitating metastasis. The EMT process may be influenced by FGF19 through the activation of specific signaling pathways. Approximately 30% of individuals diagnosed with HCC are observed to have abnormalities in the FGF19-FGFR4 signaling cascade. Specifically, upon binding to FGFR4, FGF19 initiates the activation of the GSK3β/β-catenin/E-cadherin signaling axis. This activation subsequently upregulates the expression of genes associated with cellular proliferation while concurrently inhibiting apoptotic processes, ultimately increasing the invasive capability of HCC cells 150. Therefore, the interaction between FGF19 and EMT is of great significance for understanding the metastatic mechanisms of tumor cells. Furthermore, in a zebrafish model that replicates HNSCC, the ablation of the human FGF19 homolog, Fgf15, was found to significantly reduce tumor cell migration 151.

### Influencing the Tumor Microenvironment

FGF19 plays a crucial role in shaping the tumor microenvironment through its interactions with various cell types. Specifically, it can induce the differentiation of hepatic stellate cells into inflammatory fibroblasts. When FGF19 binds to the receptors on hepatic stellate cells, it activates a signaling pathway that alters gene expression profiles. This leads to the transformation of these cells into inflammatory fibroblasts, which then secrete a variety of cytokines, chemokines, and growth factors 114. These secreted factors create an inflammatory and pro-tumorigenic microenvironment, promoting angiogenesis and recruiting immune cells that can either support or suppress tumor growth. This environment is conducive to the survival, proliferation, and invasion of tumor cells, thereby promoting tumor progression. Angiogenesis, in particular, supplies the tumor with increased oxygen and nutrients, facilitating rapid tumor growth and metastasis 152.

In the context of LSQ, FGF19 binding to FGFR4 can also promote the expression of vascular endothelial growth factor (VEGF), increasing tumor vascular density and creating favorable conditions for tumor cell proliferation and metastasis 136. Inflammatory cancer-associated fibroblasts (iCAFs) induced by FGF19 can secrete immunosuppressive factors such as interleukin-10 (IL-10) and transforming growth factor-β (TGF-β), which dampen anti-tumor immune responses. In CRC liver metastasis, TGF-β secreted by these iCAFs has been shown to inhibit the proliferation and function of CD8+ T cells, reducing the body's immune surveillance against the tumor 114. FGF19 also modulates the recruitment and polarization of immune cells within the tumor microenvironment. For example, the chemokine CCL2, secreted by FGF19-induced iCAFs, acts as a chemoattractant for monocytes and macrophages. Moreover, the FGF19/FGFR4 signaling pathway influences the polarization state of macrophages, promoting the M2 polarization of tumor-associated macrophages (TAMs). These M2-polarized TAMs, characterized by their immunosuppressive phenotype and pro-angiogenic functions, contribute to the establishment of an immunosuppressive environment that supports tumor growth and metastasis 153.

The activation of the FGF19/FGFR4 signaling pathway increases the infiltration of TAMs and myeloid-derived suppressor cells (MDSCs) while decreasing the accumulation of CD8+ T cells, leading to the formation of an immunosuppressive microenvironment that favors tumor progression 154. Additionally, the FGF19/FGFR4 signaling pathway activates the PI3K/AKT pathway, which in turn can promote the expression of IGF2BP1. Subsequently, PD-L1 mRNA is further stabilized by IGF2BP1, resulting in increased PD-L1 protein expression. Through this mechanism, recognition and attack by T cells is evaded by tumor cells within the immune microenvironment, thereby enhancing tumor immune evasion via immune checkpoint pathways 155. Lenvatinib, a targeted therapy, can downregulate PD-L1 via the FGFR4-GSK3β axis, promoting PD-L1 degradation and enhancing antitumor immunity. This intervention restores T-cell-mediated cytotoxicity in IFN-γ-pre-treated HCC cells. Lenvatinib also inhibits STAT5 phosphorylation, reduces Treg differentiation and infiltration, and modulates the tumor immune microenvironment by promoting GZMK+CD8 T cell infiltration and reducing TIM-3 and CTLA-4 expression on Tregs, further enhancing the antitumor response 156.

In CRC, a positive correlation between FGF19 expression and NK cell infiltration has been observed, whereas a negative correlation exists with the infiltration of neutrophils, regulatory T cells (Tregs), Th1/Th2 cells, B cells, helper T cells, activated dendritic cells, cytotoxic cells, and macrophages. The formation of neutrophil extracellular traps, which are fibrous structures released by neutrophils that can encapsulate tumor cells and thereby shield them from immune cell attack, can be promoted by FGF19. By enhancing neutrophil extracellular trap formation, FGF19 can inhibit the functions of NK cells and cytotoxic T cells, preventing their interaction with tumor cells and thus protecting tumor cells from immune attack 157. High FGF19 expression is associated with reduced proportions of immune cells and may drive tumor progression by suppressing immune activity. Therefore, FGF19 is considered a significant factor in tumor progression and immune evasion, representing a potential target for immunotherapeutic strategies.

### Interaction with Signaling Pathways

**Figure 4:**
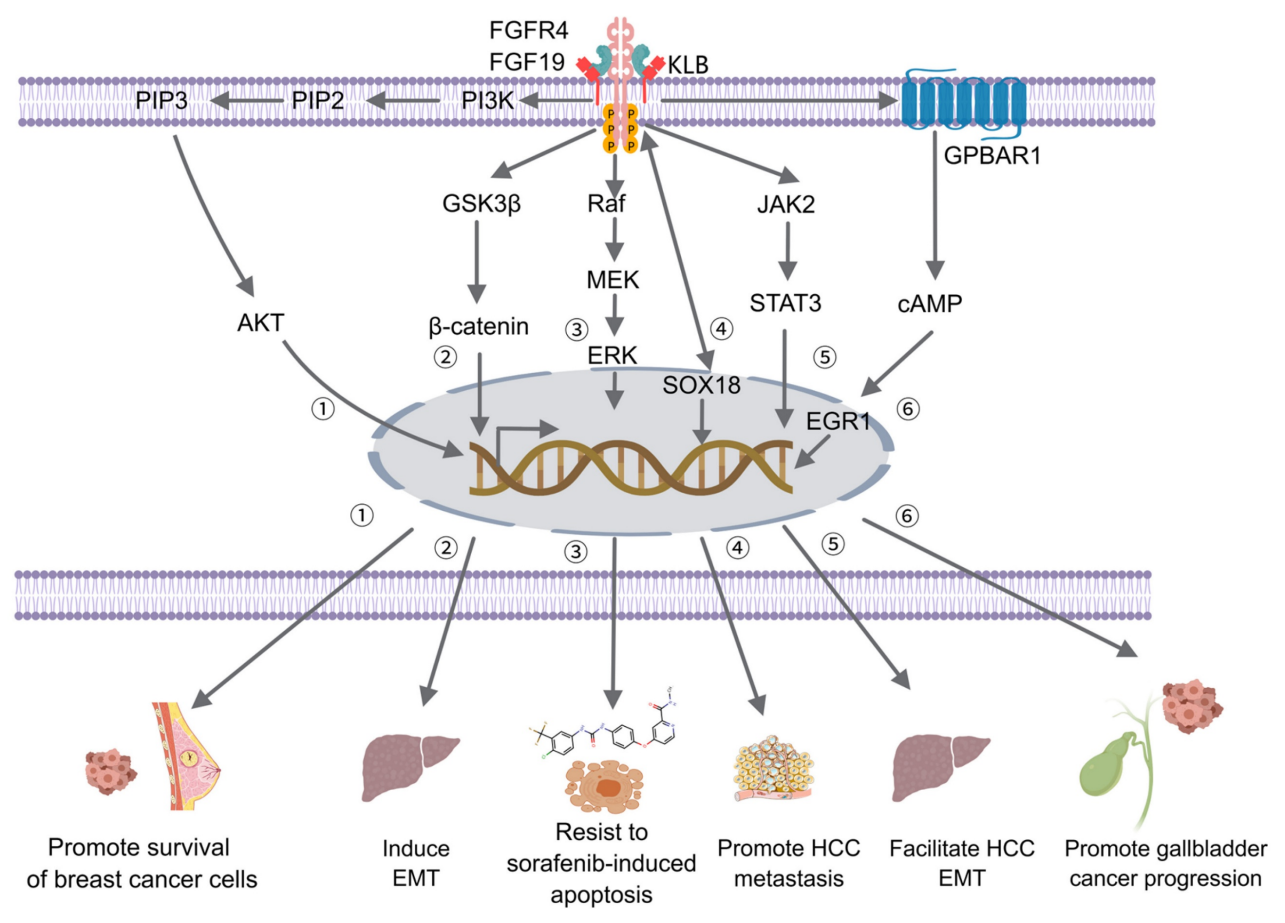
** Signaling pathways of FGF19/FGFR4.** The FGF19/FGFR4 signaling pathway is critical in the regulation of various cellular processes and disease states. Its activation begins when FGF19 binds to FGFR4, thereby leading to dimer formation through autophosphorylation and triggering multiple downstream cascades. (1) In breast cancer, it activates the PI3K/AKT pathway, which is responsible for the phosphorylation of PIP2, generating PIP3, and activating AKT, a process beneficial to tumor cell survival. (2) FGF19 is also responsible for the induction of epithelial-mesenchymal transition (EMT) in hepatocellular carcinoma (HCC) cells through the modulation of the GSK3β/β-catenin pathway by means of FGFR4 activation. (3) The binding of FGF19 to FGFR4 activates the Raf-MEK-ERK pathway, which confers resistance to sorafenib-induced apoptosis. (4) In HCC, the FGF19-SOX18-FGFR4 loop is integral to HCC metastasis. (5) The FGF19/JAK2/STAT3 pathway also contributes to the EMT of HCC. (6) Additionally, the GPBAR1-cAMP-EGR1 axis-dependent autocrine pathway is significant in promoting gallbladder cancer progression. The image was created with MedPeer.cn.

FGF19 significantly interacts with crucial signaling pathways related to cancer, such as the PI3K/AKT and ERK/MAPK signaling pathways. The activation of FGFR4 by FGF19 has been demonstrated to induce PI3K activation within the PI3K/AKT pathway. Phosphorylation of phosphatidylinositol-4,5-bisphosphate (PIP2) by activated PI3K results in the generation of phosphatidylinositol-3,4,5-trisphosphate (PIP3), subsequently recruiting and activating AKT 158. The activation of AKT can mediate BC cell survival 46 (Figure 4). The Ras/Raf/MAPK (MEK)/ERK pathway can activate ERK1/2 through a cascade of reactions, promoting cell proliferation and survival. It regulates the expression of MMPs to enhance the invasive and metastatic capabilities of tumor cells. Additionally, transcription factors are activated, leading to increased expression of VEGF, thereby promoting tumor angiogenesis and supporting tumor growth and metastasis 159. The ERK/MAPK pathway can be activated through the binding of FGF19 to FGFR4, triggering the Raf-MEK-ERK cascade. Activated ERK then translocates into the nucleus and phosphorylates various transcription factors, which regulate gene expression involved in tumor cell proliferation, differentiation, and survival 160. The FGF19-FGFR4 axis can bypass the inhibition of the Raf-MEK-ERK pathway imposed by tyrosine kinase inhibitors (TKIs). In doing so, it maintains ERK phosphorylation. This process promotes the growth and survival of HCC, ultimately leading to resistance to sorafenib 161 (Figure 4). The interaction between FGF19 and the ERK/MAPK pathway can enhance the mitogenic and oncogenic potential of tumor cells. Overall, the cross-talk between FGF19 and these signaling pathways plays a complex yet crucial role in tumor development and progression.

Tumor progression can also be promoted via an FGF19-FGFR4-initiated GPBAR1-cAMP-EGR1 axis-dependent autocrine pathway in gallbladder carcinoma 162. Besides, the FGFR4/GSK3β/β-catenin pathway appears to be crucial for FGF19-induced EMT in HCC cells (Figure 4). FGF19 levels are notably increased and inversely related to E-cadherin expression. E-cadherin expression is suppressed by ectopically expressed FGF19, driving EMT and invasion in epithelial-like HCC cells. In contrast, reducing FGF19 in mesenchymal-like HCC cells boosts E-cadherin expression and reduces EMT features. Moreover, while FGF19 depletion fails to reverse EMT traits when GSK3β inhibitors are present, knocking out FGFR4 substantially diminishes FGF19-induced EMT 163.

Additionally, the FGF19-SOX18-FGFR4 signaling loop is pivotal in the promotion of HCC metastasis. Specifically, activation of the SOX18 promoter is facilitated via the p-FRS2/p-GSK3β/β-catenin pathway, resulting in increased SOX18 expression 164. Elevated levels of SOX18 subsequently boost the transcription of FGFR4 and FLT4, creating a positive feedback mechanism that amplifies the invasive and metastatic potential of HCC cells. SOX18 overexpression is strongly correlated with poor tumor differentiation, TNM stage, and unfavorable prognosis in HCC. HCC invasion and metastasis are driven by the upregulation of metastasis-related genes, and HCC cell proliferation is stimulated while apoptosis is inhibited through modulation of the AMPK/mTOR pathway. In human HCC tissues, SOX18 expression shows a positive correlation with FGF19, FGFR4, and FLT4 expression. Patients coexpressing FGF19/SOX18, SOX18/FGFR4, or SOX18/FLT4 exhibit the poorest prognosis 165. These findings highlight the critical role of the FGF19-SOX18-FGFR4 signaling loop in HCC progression and prognosis, indicating its potential as a therapeutic target (Figure 4). The role of the FGF19/JAK2/STAT3 pathway is also of great significance. Activation of the JAK-STAT pathway enhances pro-inflammatory cytokines like IL-6 and TNF-α, driving inflammation and tumor growth. It forms a positive feedback loop to promote tumor development further. It can also enhance enzymes such as MMP-2 and MMP-9, which degrade the extracellular matrix, thereby aiding cancer cell invasion and metastasis. Additionally, it regulates cell adhesion molecules, such as E-cadherin, which facilitates the spread of cancer cells 166. The crosstalk between FGF19 and the JAK-STAT pathway is a crucial link that further elucidates the complex signaling network driving tumor progression. ER stress is triggered by the small hepatitis B virus surface antigen protein, resulting in the activation of ATF4. Once activated, ATF4 boosts the expression and secretion of FGF19. The autocrine release of FGF19 then activates the JAK2/STAT3 signaling pathway. The activation subsequently induces EMT in HCC cells, significantly enhancing their migratory capacity, invasiveness, and metastatic potential 167 (Figure 4).

Future research should focus on elucidating the detailed mechanisms through which FGF19 interacts with various signaling pathways and networks in different tumor types. Understanding these interactions could lead to the development of targeted therapies that disrupt FGF19-driven oncogenic processes.

## Expression and Mechanistic Roles of FGF19 in Various Tumors

The understanding of FGF19 mentioned above is primarily focused on its diverse roles in tumor metabolism reprogramming, proliferation, invasion, metastasis, and modulation of the tumor microenvironment. Collectively, they underscore the significant and multifaceted influence of FGF19 on cancer biology. However, it is becoming increasingly clear that the oncogenic impact of FGF19 is not a one-size-fits-all phenomenon but rather a highly context-dependent process that varies significantly across different tumor types. In this section, we aim to dissect the tumor-specific mechanisms by which FGF19 contributes to oncogenesis and tumor progression in various cancers. Elucidating these distinct pathways can provide a more granular and comprehensive understanding of FGF19's role in cancer, ultimately revealing potential therapeutic vulnerabilities that can be exploited to target this versatile oncogenic factor more effectively.

### Hepatocellular Carcinoma

HCC represents the most prevalent form of liver cancer and a leading cause of cancer-related mortality worldwide. According to recent data, HCC accounts for approximately 90% of all primary liver cancers and is responsible for about 1 million deaths annually 168, 169. The incidence of HCC exhibits global variation, with peak rates being observed in regions such as Asia and Africa, where chronic hepatitis B and C infections are endemic 170. Notably, the incidence of HCC has been rising in Western countries, largely due to the escalating prevalence of MAFLD and its progression to NASH 171. This etiological shift underscores the need for a deeper understanding of the molecular mechanisms underlying HCC development and progression, including the role of FGF19.

FGF19 is frequently overexpressed in HCC, with its elevated expression attributed to amplification of the FGF19 gene and increased transcriptional activity. It has been demonstrated through studies that, in comparison to normal liver tissues, the mRNA and protein levels of FGF19 in HCC samples can be several-fold higher 11. This overexpression is correlated with distinct genetic alterations within HCC. The abnormal activation of the Wnt/β-catenin signaling pathway, primarily through mechanisms such as APC gene mutation or deletion, overexpression or mutation of β-catenin, and overexpression of Wnt ligands, leads to uncontrolled cell proliferation, inhibition of apoptosis, and promotion of tumor invasion and metastasis 172. Specifically, in the context of HCC, FGF19 has been shown to interact with the Wnt/β-catenin pathway, further promoting tumor progression through cross-talk mechanisms that enhance the oncogenic potential of both signaling networks. Genetic mutations, particularly in genes such as β-catenin, can activate downstream signaling pathways, which subsequently upregulate the expression of FGF19. The Wnt/β-catenin pathway, which is an established oncogenic pathway in HCC, when activated, may bind to specific response elements within the FGF19 promoter region, thereby enhancing its transcription 173, 174.

**Figure 5:**
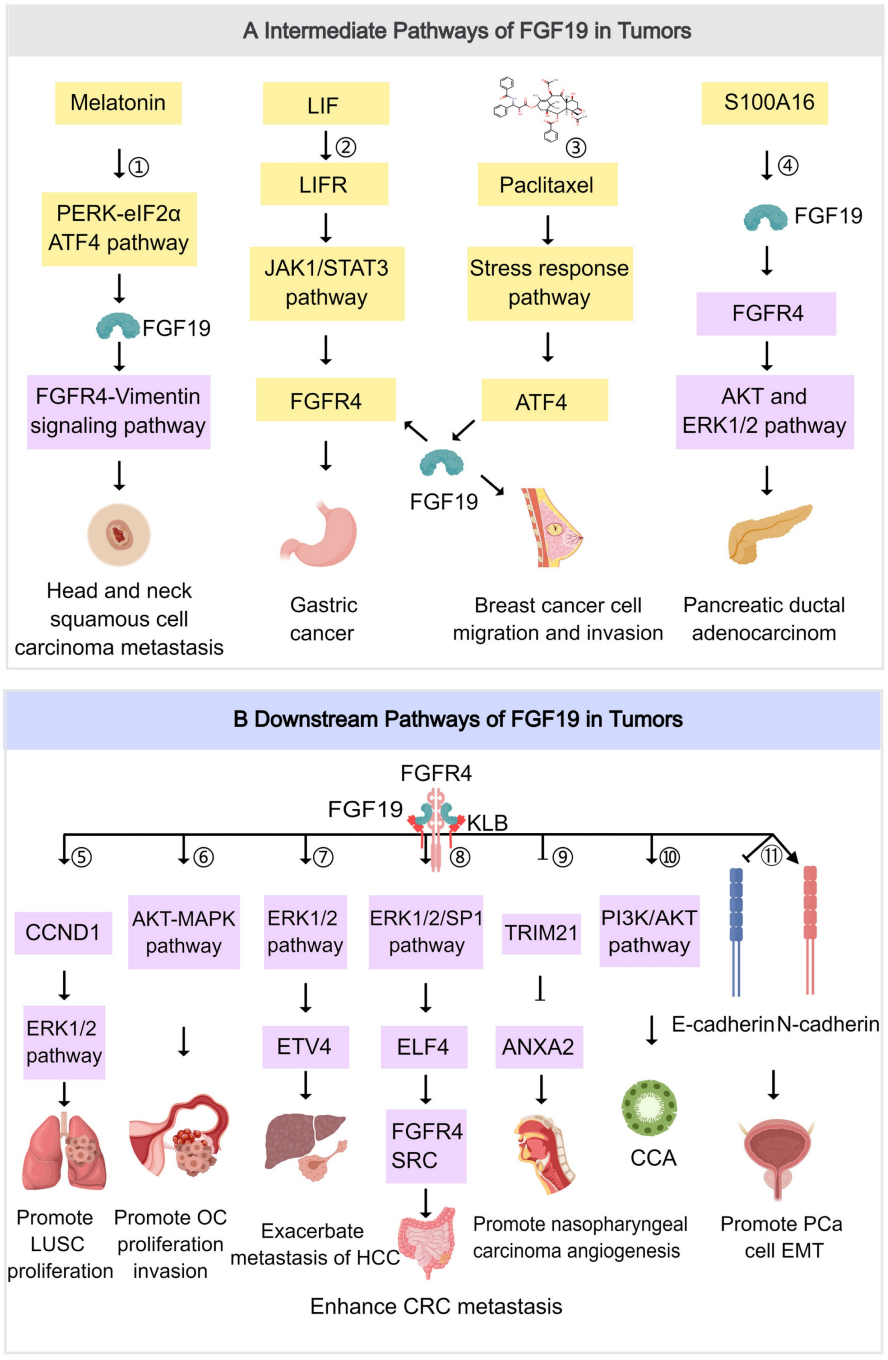
** The multifaceted roles of FGF19 in promoting cancer progression through various signaling pathways.** (1) In head and neck squamous cell carcinoma, high doses of melatonin activate the PERK-eIF2α-ATF4 pathway, thereby upregulating FGF19 and promoting FGFR4-Vimentin signaling, which reduces anti-metastatic activity. (2) In gastric cancer, the LIF-LIFR-JAK1/STAT3 pathway upregulates FGFR4, with FGF19 enhancing tumorigenesis. (3) In breast cancer, paclitaxel induces stress response pathway, which leads to an increase in ATF4 and FGF19, promoting cell migration and invasion. (4) In pancreatic ductal adenocarcinoma, S100A16 drives progression via the AKT and ERK1/2 pathways, which are dependent on FGF19. (5) In lung squamous cell carcinoma (LUSC), FGF19 co-amplifies with CCND1, activates ERK1/2 via FGFR4, promoting cell proliferation. (6) In ovarian cancer (OC), the FGF19-FGFR4 axis is responsible for the promotion of proliferation and invasion via the AKT-MAPK pathway. (7) In hepatocellular carcinoma (HCC), FGF19 binds FGFR4 to activate ERK1/2, thereby upregulating ETV4 and leading to the worsening of metastatic activity. (8) In colorectal cancer (CRC), FGF19 upregulates ELF4 via the ERK1/2/SP1 axis, thereby promoting metastasis through FGFR4 and SRC. (9) In nasopharyngeal carcinoma, FGF19 is correlated with microvascular density and stimulates angiogenesis via the suppression of TRIM21-mediated ANXA2 ubiquitination. (10) In cholangiocarcinoma (CCA), FGF19 plays a role in the mediation of the PI3K/AKT pathway, contributing to disease progression. (11) In prostate cancer (PCa), FGF19 activates its receptor to inhibit E-cadherin and promote N-cadherin, thereby driving the epithelial-mesenchymal transition (EMT) process. The image was created with MedPeer.cn.

Within the tumor microenvironment of HCC, FGF19 is primarily expressed by cancer cells; however, it can also be secreted and affect surrounding stromal cells. For instance, cancer-associated fibroblasts (CAFs) can respond to FGF19 from HCC cells 175. These CAFs then contribute to tumor progression by secreting factors such as extracellular matrix-remodeling enzymes and growth factors that support tumor growth and metastasis 176. Moreover, FGF19 activates FGFR4, triggering the PI3K/AKT pathway and stabilizing HIF1α, which upregulates HOXB5 expression. HOXB5 then enhances the expression of FGFR4 and CXCL1. Increased FGFR4 fosters HCC cell proliferation and metastasis, while the CXCL1/CXCR2 complex recruits MDSCs, suppressing antitumor immunity and facilitating metastasis. In mice, FGF15, the analog of FGF19, promotes HCC metastasis via HOXB5. HOXB5 knockdown impairs this effect, highlighting its key role in FGF19/FGF15-mediated HCC metastasis 177. Additionally, the FGF19/FGFR4 signaling pathway can promote store-operated calcium entry (SOCE) through the activation of the PLCγ and ERK1/2 pathways. Subsequently, the SOCE-calcineurin signaling pathway is activated, which in turn facilitates the activation and nuclear translocation of nuclear factor of activated T cells (NFAT)-c2. NFATc2 activation further upregulates the transcription of stemness-related genes such as NANOG, OCT4, and SOX2, as well as the expression of FGF19 itself. This molecular cascade enhances the self-renewal capacity of liver cancer stem cells, thereby playing a critical role in the initiation and progression of HCC 178. The pathway also drives IGF2BP1 expression, which promotes the expression of PD-L1, thereby contributing to immune escape 155. FGF19 is capable of promoting angiogenesis, which is essential for tumor metastasis, by stimulating VEGF production in both HCC and stromal cells. Newly formed blood vessels provide routes for cancer cells to enter the circulation and metastasize to distant organs 175. Additionally, when FGF19 binds to FGFR4, it activates the ERK1/2 signaling pathway, resulting in the upregulation of ETV4. ETV4, activated by the FGF19/FGFR4 pathway, further enhances pathway activity by upregulating FGFR4, forming a positive feedback loop. This mechanism increases ETV4 expression, thereby exacerbating HCC metastasis 154 (Figure 5).

In addition, FGF19-mediated signaling can contribute to drug resistance in HCC. Overexpression of FGF19 suppresses sorafenib-induced ROS production and apoptosis, thereby enabling cancer cells to evade the cytotoxic effects of sorafenib. Conversely, the loss of the FGF19/FGFR4 pathway markedly boosts sorafenib-induced ROS and apoptosis, highlighting the critical role it plays in sustaining HCC cell survival and conferring resistance to sorafenib 161.

Advances in HCC research also elucidate the interaction between gut microbiota, bile acids, and the FGF19 signaling pathway. Bile acids, which are synthesized in the liver, are regulated through the FXR-FGF19 axis and GPBAR1/TGR5 signaling pathways. The gut microbiota can modulate bile acid metabolism, converting them into secondary bile acids, which subsequently influence host physiology and immune cell differentiation. For example, 3-oxo-LCA can inhibit Th17 cells, whereas iso-LCA can promote Treg cells. Hydrophobic bile acids like DCA, linked to DNA damage and cell survival, have been demonstrated to drive HCC development. In (nonalcoholic steatohepatitis-associated) NASH-HCC, bile acid-metabolizing bacteria, such as Clostridium and Bacteroides, are correlated with elevated TCA levels; however, reducing hydrophobic bile acids can reverse disease progression. Furthermore, bile acid conversion mediated by the gut microbiota influences natural killer T cell accumulation and HCC-related immune responses via the CXCL16-CXCR6 signaling axis 91. The FGF19 pathway, which is crucial for bile acid synthesis and liver homeostasis, may be indirectly influenced by the gut microbiota. Modulation of the gut microbiota and bile acid metabolism via the FGF19 pathway may represent a potential future strategy for therapeutic intervention in HCC.

Thus, FGF19 serves a multifaceted role in promoting proliferation, metastasis, immune evasion, and drug resistance in HCC, acting as a pivotal mechanism underpinning HCC progression and therapeutic resistance. Consequently, targeting the FGF19/FGFR4 pathway emerges as a potential therapeutic strategy for HCC treatment.

### Colorectal Cancer

CRC ranks as the third most common cancer globally and is the second leading cause of cancer-related deaths. It is estimated that approximately 1.93 million new cases of CRC will be diagnosed worldwide in 2025 179. While overall incidence rates in many developed countries are declining due to effective screening and improved treatments, the incidence of early-onset CRC among individuals under 50 is rising alarmingly. This form of CRC often presents as more aggressive and is associated with poorer outcomes 180.

FGF19, a key player in CRC progression and liver metastasis, exerts its effects through a multitude of intricate mechanisms. The overexpression of FGF19 in CRC cells has been correlated with liver metastasis and a decreased overall survival (OS) in patients 181. By engaging the FGF receptor and β-klotho co-receptor complex, FGF19 activates signaling cascades, which are fundamental to cellular proliferation, differentiation, survival, and migration. In the context of CRC, it promotes cancer cell proliferation and survival, fosters angiogenesis, modulates bile acid metabolism, thereby influencing the intestinal microenvironment and augmenting inflammation, and regulates intestinal stem cells 182. Moreover, FGF19 enhances ELF4 expression via the ERK1/2/SP1 pathway. When ELF4 is overexpressed, it activates the transcription of its downstream genes, FGFR4 and SRC, which in turn drive CRC metastasis 183 (Figure 5). Furthermore, CRC cells overexpressing FGF19 display enhanced liver metastatic capacity. FGF19 modulates the immune microenvironment, fostering conditions conducive to colorectal carcinoma liver metastasis (CRCLM). This modulation induces the polarization of hepatic stellate cells into iCAFs by triggering the autocrine action of IL-1α via the FGFR4-JAK2-STAT3 pathway. These FGF19-induced iCAFs stimulate neutrophil infiltration and neutrophil extracellular trap formation within liver metastatic niches by releasing complement C5a and IL-1β, thereby facilitating the liver colonization of CRC cells 181. Single-cell RNA sequencing (scRNA-Seq) studies have demonstrated significant interactions mediated by FGF19 between CRC cells and CAFs, leading to the activation of hepatic stellate cells and their differentiation into CAFs. Importantly, the FGF19/ANGPTL4 axis has been identified as critical to CRCLM, with disruption of this axis significantly inhibiting CRCLM *in vivo* 114. ScRNA-Seq studies also reveal that FGF19 expression exhibits significant cellular heterogeneity, being primarily detected in various cell types, including squamous epithelial cells, myeloid cells, stromal cells, T cells, NK cells, and innate lymphoid cells (ILCs). Within immune cells, FGF19 expression is particularly concentrated in monocytes, cytotoxic T lymphocytes, cells expressing promyelocytic leukemia zinc finger protein, dendritic cells, and macrophages. Notably, FGF19 is highly expressed in cytotoxic T lymphocytes, CD4+ T cells, and cells expressing promyelocytic leukemia zinc finger protein. It elucidates the intercellular interactions of FGF19 within the tumor microenvironment, suggesting that it may influence the progression of CRC and the regulation of the immune microenvironment by affecting the functions and interactions of these key immune cells 157.

In the occurrence and development of CRC, the gut microbiota, bile acids, and the FGF19 signaling pathway have also been implicated. The gut microbiota can convert primary bile acids into secondary bile acids, a process that significantly influences the expression of FGF19. DCA, one of the secondary bile acids, can inhibit the expression of the FXR. While the activation of FXR can induce the expression of FGF19, which can inhibit the expression of CYP7A1 and reduce the synthesis of primary bile acids. However, high concentrations of secondary bile acids have been found to be cytotoxic and may induce genetic mutations as well as genomic instability in colonic epithelial cells, thereby promoting tumorigenesis. Moreover, dysbiosis of the gut microbiota has been associated with inflammation and insulin resistance, both of which contribute to the progression of CRC 81. Therefore, maintaining the balance of the gut microbiota is crucial in the regulation of bile acid metabolism and the FGF19 signaling pathway, as well as the prevention of CRC.

Collectively, these findings underscore the multifaceted role of FGF19 in CRC development and progression, making it an appealing target for novel therapeutic strategies. Further exploration of the intricate mechanisms underlying FGF19's actions and the development of more potent FGF19-targeted therapies are imperative for improving patient outcomes in this disease.

### Breast Cancer

BC is recognized as the leading malignant tumor affecting women globally, with an estimated 2.3 million new cases reported in 2022, which account for 23.8% of all new cancer cases 184, 185. The incidence of BC continues to increase, posing significant challenges to global cancer prevention and treatment efforts. Metastasis, rather than the primary tumor, represents the primary cause of mortality among patients diagnosed with BC 186. Despite the availability of numerous treatment options, including surgery, chemotherapy, radiation therapy, and targeted therapies, the high incidence and poor prognosis associated with metastatic disease underscore the necessity to further explore and comprehend the molecular mechanisms driving BC progression.

The progression of BC involves the upregulation of FGF19 expression following chemotherapy, especially with the use of paclitaxel. Paclitaxel, a widely utilized chemotherapeutic agent, not only enhances the migration and invasion of BC cells but also triggers a stress response pathway that upregulates FGF19 expression. This phenomenon suggests a link between paclitaxel treatment and altered FGF19 levels. Furthermore, the ATF4, interacting with the stress response, is posited to facilitate this effect by regulating FGF19 expression 16 (Figure 5). Consequently, increased levels of FGF19 promote aggressive behavior in BC cells. The oncogenic effects of FGF19 are exerted through its binding to FGFR4 on BC cells, thereby activating the AKT signaling pathway 187. AKT signaling is pivotal for cell survival, proliferation, and migration, and its upregulation by FGF19 is instrumental in promoting the malignant behavior observed in these cells 188. Notably, FGF19 is highly expressed in the luminal molecular subtype of breast tumors, and its levels are found to correlate with secretion from cancer cells. Genetic knockout of FGF19 or inhibition of FGFR4 has been demonstrated to inhibit breast tumor progression and metastasis in mouse models, suggesting that targeting FGFR4 may be a viable therapeutic strategy for cancer suppression. Additionally, some breast cancers are resistant to doxorubicin due to frequent elevation of FGFR4 expression, which enhances resistance by upregulating anti-apoptotic protein Bcl-xL via the MAPK pathway 189. Exploring the role of the FGF19/FGFR4 signaling pathway in breast cancer drug resistance, developing specific targeted drugs, optimizing combination therapy strategies, and exploring new treatment technologies are expected to offer more effective treatment options for breast cancer patients and help overcome the challenge of drug resistance.

There are also research reports that indicate that in a subset of basal-like BC cells, FGF19 is secreted in an autocrine manner, binding to FGFR4 and activating the PI3K/AKT signaling pathway. This activation fosters the survival of cancer cells and their resistance to chemotherapy. The inhibition of FGF19, achieved through methods such as siRNA-mediated silencing or neutralization by an anti-FGF19 antibody, specifically decreases AKT phosphorylation, suppresses cancer cell growth, and enhances doxorubicin sensitivity in FGFR4+/FGF19+ BC cells 46. It is implied by these findings that the disruption of the FGFR4/FGF19 autocrine signaling loop may present a novel therapeutic approach for treating BC, particularly those with a basal-like phenotype. Moreover, FGF19's impact on BC may also be influenced by its role in systemic and microenvironmental metabolism 46. The ablation of FGFR4 results in increased levels of FGF21, which is another member of the FGF family with metabolic effects akin to FGF19. These alterations affect adipogenesis and the secretory function of adipocytes, which in turn influence lipogenesis, glycolysis, and energy homeostasis within breast tissue and tumor foci. The metabolic reprogramming of breast epithelial cells caused by these changes contributes to the suppression of tumor progression, suggesting that the tumor-delaying effect of FGFR4 deficiency may be largely attributed to the elevated anti-obesogenic FGF21 and its resultant metabolic effects 190. Additionally, studies have suggested a possible connection between FGF19 and Crohn's disease (CD), as both conditions share common genetic and inflammatory pathways. In CD patients, FGF19 and other genes, such as B4GALNT2, have been identified as differentially expressed and possessing prognostic significance in BC. While the exact mechanism by which FGF19 influences BC prognosis in CD patients has not yet been clarified, it is speculated to be part of the intricate interplay between chronic inflammation, immune responses, and gene expression changes associated with both conditions 191.

The role of FGF19 in BC progression renders it a promising therapeutic target. Inhibiting FGF19 or its associated signaling pathways can slow tumor growth and metastasis by reducing the proliferation, migration, and invasion of BC cells. Neutralizing antibodies against FGF19, or siRNA knockdown of FGF19, may offer a highly specific approach through binding to FGF19 and preventing its interaction with FGFR4, consequently inhibiting downstream signaling while simultaneously enhancing chemotherapeutic sensitivity.

### Prostate Cancer

PCa is considered one of the most prevalent cancer types among males worldwide, characterized by a significant incidence rate 192. According to the World Health Organization, it holds the second position among the most common cancers affecting men globally, with annual diagnoses exceeding 1.4 million cases. Geographic variations in PCa incidence are evident, with higher rates reported in developed nations such as the United States and Western Europe 193. Despite advancements in early detection through prostate-specific antigen (PSA) testing and subsequent improvements in survival rates, PCa continues to be a leading cause of cancer-related mortality among males 194.

FGF19 has been identified as a significant contributor to the progression of PCa. Its role in PCa is multifaceted, affecting cell proliferation, EMT, and apoptosis. Specifically, FGF19 promotes the viability and EMT of PCa cells, as demonstrated by increased cell survival, elevated expression of N-cadherin, a marker of EMT, and reduced levels of E-cadherin in PCa cell lines such as LNCaP and PC3 under conditions of androgen deprivation 195 (Figure 5). In clinical settings, elevated serum levels of FGF19 are associated with higher Gleason grades of PCa, a measure of cancer aggressiveness. Immunohistochemical analysis further supports these findings, revealing that FGF19-positive tissues are correlated with higher PSA levels and a poorer prognosis, including decreased 5-year biochemical recurrence-free survival rates after radical prostatectomy 195. These findings suggest that FGF19 may contribute to the progression and recurrence of PCa through the modulation of cell proliferation and EMT.

Mechanistically, FGF19 functions as an autocrine growth factor in PCa, being expressed in both primary and metastatic PCa tissues. Silencing FGF19 in PCa cells that express autocrine FGF19 results in reduced invasion and proliferation *in vitro*, as well as tumor growth *in vivo*, highlighting its oncogenic potential. FGF19 mediates its oncogenic effects via classic FGF receptors, aided by the coreceptors α-Klotho and/or KLB, which are also expressed in PCa cells 17. This signaling pathway proves to be critical for the oncogenic effects of FGF19 in PCa. The role of FGF19 in PCa extends beyond the autocrine function alone; it is identified among the secretory proteins in the secretome of a patient-derived xenograft, MDA-PCa-118b, derived from an osteoblastic bone lesion in human PCa. Within the tumor microenvironment, FGF19 serves dual roles as an autocrine and a paracrine factor, impacting PCa-118b tumor cells and stromal cells, including endothelial cells and osteoblasts 196. This suggests that FGF19 participates in the complex signaling networks that support metastatic growth in bone, potentially contributing to the development of osteoblastic bone metastases in PCa. Given its role in promoting EMT and bone metastasis, targeting FGF19 along with its related signaling pathways may present a novel therapeutic strategy for PCa, with the potential to mitigate cancer progression, recurrence, and the onset of bone metastases. Continued research is vital for further elucidating the mechanisms through which FGF19 influences PCa and for developing efficacious therapies targeting this oncogenic factor.

### Ovarian Cancer

OC stands as a major gynecological malignancy worldwide, with approximately 239,000 new cases each year 197. It has a high mortality rate owing to the absence of early symptoms and effective screening methods. Consequently, the majority of OC cases are identified at an advanced stage, by which time the cancer has often spread beyond the ovarian confines. Among the various subtypes, epithelial ovarian cancer (EOC) is the most common, accounting for approximately 90% of all OC instances 198. Despite advancements in therapeutic interventions, including surgery and chemotherapy, the overall five-year survival rate remains relatively low, highlighting the urgent necessity for innovative therapeutic strategies 199.

Studies have shown that FGF19 is expressed in OC tissues. Hu Lingling and Cong Lanxiang found that high expression of FGF19 and FGFR4 in advanced serous OC is predictive of poor prognosis 200. The dual high expression of FGF19-FGFR4 serves as a more sensitive prognostic indicator for advanced serous ovarian cancer. FGF19 is associated with OC cell proliferation 201. Carcinogenic factors elevate FGF19 gene expression in OC tissues, stimulating excessive FGF19 synthesis and enhancing angiogenesis, which fuels cancer growth. Zhang Yi et al. found that exogenous aFGF and bFGF enhance OVCA3 cell proliferation, with a positive correlation between their concentrations. Specifically, 1.2 μg/ml aFGF and 100 ng/ml bFGF increased the proliferation of OVCA3 cells by factors of 1.51 and 1.85, respectively, compared to controls 202. Besides, FGF19 is intimately linked with ovarian cancer, particularly in high-grade serous ovarian cancer (HGSOC), where it is often amplified and overexpressed. FGF19 enhances cisplatin resistance by inducing autophagy via the following mechanism. FGF19 activates the p38 MAPK signaling pathway, which in turn induces autophagy. Studies have demonstrated that silencing FGF19 inhibits autophagy in ovarian cancer cells. This is characterized by reduced expression of autophagy-related proteins such as LC3 and Beclin 1, while the expression of SQSTM1/p62 is upregulated. Furthermore, silencing FGF19 leads to decreased phosphorylation of p38 MAPK, an effect that can be reversed by IFN-γ, a well-known activator of p38 MAPK 18. Moreover, overexpression of FGF19 predicts a poor prognosis for patients with advanced-stage serous OC. It promotes cancer cell proliferation and invasion through the FGFR4-AKT-MAPK signaling pathway 200 (Figure 5). High FGF19 expression has been found in AZD4547-resistant OC cells. Combining AZD4547 with FGF19 siRNA or FGFR4 inhibitor results in reduced cell proliferation, suggesting a strategy to overcome resistance by targeting FGF19/FGFR4 203. These findings provide a novel therapeutic target for the treatment of OC. However, to fully elucidate the mechanisms underlying the effects of FGF19 on OC, further research remains essential. This includes molecular and cellular studies to explore the intricate interactions between FGF19 and its receptors, along with the downstream signaling pathways in OC cells.

### Gastric Cancer

GC remains one of the most prevalent and lethal malignancies worldwide. According to the latest global cancer statistics, it is the fifth most frequently diagnosed cancer and ranks third among the leading causes of cancer-related deaths 204, 205. The high incidence and mortality rates are largely attributed to its asymptomatic nature during the early stages, leading to late diagnosis and poor prognosis. Thus, understanding the molecular mechanisms underlying GC progression is essential for the development of effective therapeutic strategies.

FGF19 is upregulated in GC and correlates with factors including tumor invasion depth, lymph node involvement, and TNM staging. It has been experimentally demonstrated in controlled environments to enhance the migratory and invasive capabilities of GC cells 19. This leads to the upregulation of downstream targets, such as ERK1/2 and AKT, which are well-known regulators of cell growth and division 206. FGF19 promotes tumorigenesis by stimulating proliferation, inhibiting apoptosis, and promoting angiogenesis 207. It influences GC via a regulatory loop involving leukemia inhibitory factor (LIF), LIFR, FGFR4, and STAT3. In GC cells, LIF binds to LIFR to activate JAK1/STAT3, causing upregulation of FGFR4. FGF19, as the ligand for FGFR4, enhances oncogenesis 208 (Figure 5). LIR-201 inhibits LIFR, disrupting this loop by blocking STAT3 phosphorylation and FGFR4 upregulation. Additionally, Roblitinib against FGFR4 inhibits STAT3 phosphorylation and oncogenesis, indicating FGFR4 as a downstream target 208. Additionally, the impact of FGF19 on GC via FOXC1 regulation has been noted, although the direct mechanism remains unclear 209. Further investigation into the downstream signaling cascades and molecular interactions is required to elucidate the precise function of FGF19 in GC. It has been demonstrated that, at non-hepatotoxic doses, GC regulates levels of FGF-related molecules by decreasing ileal Fgf15 and increasing liver Fgf21, without affecting the expression of Fxr, which triggers an autocrine mechanism within the liver wherein GC-stimulated FGF21 inhibits CYP7A1 promoter activity. Furthermore, in mice with experimentally induced colitis, there is an additional elevation of liver Fgf21 levels. Consequently, GC disrupts the normal intestinal regulation of CYP7A1 expression in the liver, mediated by the FXR/FGF19 pathway, through this FGF21-dependent mechanism 210. Therefore, interfering with FGF19 and its associated pathways presents a potential approach for devising new treatment methods for GC. Additional studies are required to fully understand the underlying mechanisms and enhance the effectiveness of therapies targeting FGF19.

### Head and Neck Squamous Cell Carcinoma

HNSCC primarily arises from the mucosa of the oral cavity, pharynx, and larynx and is often linked to tobacco, alcohol, and HPV infection 211. It ranks as the sixth most common malignancy globally, with approximately 890,000 new cases and 450,000 deaths annually, accounting for about 4.5% of all cancer diagnoses and deaths. The incidence varies by region 212. It is an aggressive cancer that is treated with multimodal approaches, including surgery, chemoradiotherapy, and newer therapies based on recent advancements in molecular genetics.

The mechanisms by which FGF19 influences HNSCC are complex and involve both autocrine signaling and interactions with other cellular pathways. Firstly, FGF19 promotes tumorigenesis in HNSCC through autocrine signaling, where the amplification of the FGF19 gene leads to increased FGF19 secretion. This activates FGFR4-dependent signaling pathways like the ERK/AKT-p70S6K-S6 pathway, thereby enhancing cell proliferation and colony formation. Conversely, the knockout of FGF19 suppresses tumor growth in HNSCC cells with high endogenous FGF19, underscoring the therapeutic potential of targeting the FGF19/FGFR4 axis 14. Additionally, FGF19 interacts with melatonin treatment in a complex manner. High-dose melatonin activates the ER stress-associated PERK-eIF2α-ATF4 pathway, leading to upregulation of FGF19 expression in HNSCC cells, promoting FGFR4-Vimentin invasive signaling, attenuating melatonin's ability to suppress metastasis 20 (Figure 5). Long-term exposure to high-dose melatonin can induce EMT in HNSCC cells, making them more aggressive; however, genetic depletion of FGF19 and FGFR4 or treatment with an FGFR4 inhibitor can alleviate this effect. Furthermore, the dysregulation of the FGF19-KLB axis in the tumor microenvironment also plays a significant role in the progression of HNSCC. Higher expression of KLB is associated with better prognoses and longer survival times in HNSCC patients, suggesting its potential as a prognostic marker 213. Additionally, variations in drug susceptibility and immunological infiltration are observed based on KLB expression levels, indicating that KLB may influence immunological and therapeutic responses in HNSCC patients 213. Therefore, FGF19 may serve as a potential therapeutic target. However, the detailed molecular mechanisms through which FGF19 and KLB specifically interact to affect HNSCC progression and management remain to be fully elucidated through further research.

### Pancreatic Ductal Adenocarcinoma

Pancreatic cancer, particularly PDAC, is highly lethal. The 5-year survival rate has risen to approximately 13% in 2024, up from 9% a decade ago 214. However, it remains the third leading cause of cancer-related deaths in the US, with an estimated 66,440 new cases and 51,750 deaths in 2024 215. Most patients are diagnosed at advanced stages, with only 20% to 25% eligible for surgery 216. Recurrence and metastasis are common even after surgery, resulting in a poor prognosis.

In PDAC cells, FGFR4 activation induced by FGF19 contributes to tumor suppression by enhancing cell adhesion to the extracellular matrix, potentially inhibiting metastatic behavior 217. Furthermore, S100A16 is prominently expressed in PDAC and is crucial in promoting the proliferation, migration, and invasion of these cancer cells, with its effects being dependent on FGF19. FGF19 likely triggers downstream signaling pathways through its receptors, subsequently influencing the activity of S100A16. This protein is capable of activating the AKT and ERK1/2 pathways, which are vital for the metastasis and progression of PDAC 218 (Figure 5). The AKT pathway is essential for cellular survival, growth, and metabolism, whereas the ERK1/2 pathway is involved in cell proliferation, differentiation, and survival. Additionally, the reduction of S100A16 expression results in the arrest of the cell cycle in the G2/M phase and induces apoptosis in PDAC cells, underscoring its significance in supporting cell growth and viability. FGF19 indirectly affects cell cycle progression and apoptosis by modulating the expression and functionality of S100A16 21. These findings indicate the existence of some kind of interaction or signal transduction chain among FGF19, S100A16, AKT, and ERK1/2, which collectively influence the development of PDAC. Due to the overexpression of S100A16 in PDAC and its role in promoting cancer progression through FGF19-mediated signaling pathways, S100A16, as well as the potentially involved FGF19 signaling pathways, are considered promising therapeutic targets for PDAC.

### Nasopharyngeal Carcinoma

NPC is a malignant tumor of the head and neck that is more prevalent in males, with a male-to-female incidence ratio of about 2.5:1 and a peak incidence age of 40-59 years 219. The etiology is related to multiple factors, including EBV infection. Radiotherapy is pivotal for NPC, yet it is less effective against radiation-resistant tumors, which increases the risk of recurrence. Traditional treatments have shortcomings. Imprecise radiotherapy may harm normal tissues, causing issues such as dry mouth and mucositis 220. Chemotherapy might trigger nausea, vomiting, and bone marrow suppression, and surgery involves high risks and potential functional loss, particularly for recurrent cases 221. Thus, molecular targeted therapy emerges as a promising new research direction for overcoming these challenges.

High expression of FGF19 promotes the malignant behaviors of NPC cells. The expression of FGF19 correlates with microvessel density in NPC tissues, indicating its crucial role in angiogenesis. FGF19 derived from NPC cells stimulates angiogenesis both *in vivo* and *in vitro*, which is a pivotal factor for tumor growth and metastasis. Mechanistically, angiogenesis is enhanced by FGF19 through its effect on the expression of Annexin A2 (ANXA2), a protein playing a key role in this process. Notably, tripartite motif-containing 21 (TRIM21) is responsible for interacting with ANXA2 and its ubiquitination. While TRIM21-mediated ubiquitination of ANXA2 generally inhibits its function, FGF19 can hinder this process, thereby potentiating ANXA2 function and further promoting angiogenesis 13 (Figure 5). Additionally, FGF19 in MSC-derived exosomes promotes NPC progression by activating the FGF19-FGFR4-ERK signaling pathway and modulating EMT, thereby influencing NPC cells through exosome interaction. FGF19, alongside genes like PTEN, CCDN1, FGF3, and FGF4, exhibits copy number variation in NPC patients with brain metastasis, suggesting its potential role in the genomic changes driving the development or progression of this condition 222, 223. Therefore, therapeutic strategies targeting FGF19 may offer new treatment options for NPC patients by providing a non-invasive biomarker. Compared with traditional treatment methods, this strategy may exhibit improved therapeutic efficacy with fewer side effects.

### Thyroid Cancer

TC is the most common endocrine malignancy, with an increasing incidence globally 224. It includes papillary, follicular, medullary, and anaplastic types. Traditional treatments such as surgery, radioactive iodine therapy, external beam radiation, and chemotherapy have limitations, including high recurrence rates, iodine resistance, and poor efficacy in advanced cases 225. These challenges highlight the need for novel therapies. FGF19 is emerging as a potential oncogenic driver in TC, with studies suggesting its role in tumor progression.

In TC patients, immunohistochemical analysis has revealed a strikingly high rate of FGF19 protein expression in cancerous tissues compared to normal thyroid tissues. This overexpression of FGF19 is strongly associated with advanced tumor stages, including tumor-node-metastasis, extrathyroidal extension, lymph node involvement, and distant metastasis 22. As the cancer progresses, FGF19 expression increases, suggesting its potential role in the invasive and metastatic processes of TC. Moreover, experimental studies have shown that downregulating FGF19 expression through the use of siRNA-FGF19 can significantly inhibit the migration and invasion capabilities of TC cells *in vitro* 22. This finding further supports the importance of FGF19 in the progression of TC. Additionally, recent research has highlighted an intricate interplay among β-Klotho, FGF19, and FGFR-4 in serum, which may collectively contribute to the pathogenesis of TC. β-Klotho appears to function as a modulator, influencing the levels of FGF19 and FGFR4, thereby indirectly regulating FGF19 expression in TC tissues 226. Given the overexpression of FGF19 in TC tissues and its strong correlation with tumor malignancy, FGF19 holds significant potential as a molecular marker for early TC diagnosis. Detecting FGF19 expression levels could facilitate early detection of TC, thereby improving treatment outcomes and enhancing patient survival rates. Further research remains necessary to elucidate its mechanisms and therapeutic potential.

### Cholangiocarcinoma

CCA is a rare and aggressive biliary tract cancer characterized by intrahepatic, perihilar, and distal subtypes 227. Incidence rates are higher in Southeast Asia due to liver fluke infections, and a rising trend is observed in Western countries, likely linked to conditions such as primary sclerosing cholangitis and MAFLD. Treatment options are limited, with surgical intervention considered optimal for localized disease. However, most patients are diagnosed when tumors are advanced and unresectable. Administered systemic chemotherapy using gemcitabine and cisplatin provides modest benefits, yet the prognosis remains poor 228.

FGF19 is a gut hormone that regulates bile acid synthesis and promotes liver regeneration. In perihilar CCA, which originates in the bile ducts near the liver, the relationship between FGF19 and the disease is multifaceted. Bile flow is often obstructed by CCA, disrupting bile acid metabolism and the bile salt-FGF19 axis 229. Bile salts, which play a vital role in dietary fat absorption and regulate bile acid synthesis via FGF19, decline sharply postoperatively in CCA patients undergoing liver resection, indicating axis disruption. However, this disruption is more closely tied to liver function and the risk of liver failure rather than directly impacting liver regeneration. The effects of FGF19 in promoting liver regeneration are overshadowed by the disease's pathology and the impact of surgery 35. Additionally, FGF19 plays a significant role in CCA progression by mediating the PI3K/AKT signaling pathway (Figure 5). Expression of FGF19 is downregulated by lenvatinib, a multi-target drug, in CCA cells, thereby inactivating the PI3K/AKT pathway and inhibiting tumor cell proliferation, migration, and invasion 230. In contrast, the PI3K/AKT pathway activation is caused by FGF19 overexpression, which counteracts the anti-tumor effects of lenvatinib. Targeting of FGF19 by lenvatinib results in reduced proliferation markers such as Ki-67 and invasion-related proteins like vimentin and VEGF in CCA, highlighting FGF19 as a promising therapeutic target. By targeting FGF19, key aspects of tumor biology may not only be addressed, but also personalized treatment options for patients with specific molecular alterations may be offered, potentially improving prognosis in this aggressive cancer.

### Lung Squamous Cell Carcinoma

LUSC represents approximately 30% of non-small-cell lung cancer (NSCLC) cases, which predominantly affects older males and is closely linked to smoking 231. Standard treatments involve surgical removal for early-stage cases, as well as chemotherapy and radiation therapy. Despite these options, challenges are faced by current therapies, which include reduced effectiveness in advanced stages, significant side effects, limited availability of targeted treatments, and lower immunotherapy response rates compared to other lung cancer types 232. As a result, a critical need exists for more effective and targeted approaches to enhance the prognosis for LUSC patients.

FGF19 has been identified as a key oncogenic driver in the development and progression of LUSC, particularly among Chinese patients who smoke 74. Amplification and heightened expression of FGF19 in LUSC are significantly associated with poor overall and progression-free survival. Overexpression of FGF19 promotes cell growth, progression, and metastasis in LUSC cells, whereas its downregulation effectively inhibits LUSC progression both *in vitro* and *in vivo*. Mechanistically, an interaction between FGF19 and GLI2 forms a positive feedback loop that promotes LUSC cell invasion and metastasis. The interaction is crucial for driving the aggressive behavior of LUSC. Additionally, high serum levels of FGF19 can serve as a novel diagnostic index for lung cancer. Inhibiting GLI2 or the receptor FGFR4 can effectively reduce FGF19-mediated LUSC invasion and metastasis, suggesting potential therapeutic strategies for FGF19-driven LUSC 233. FGF19 is frequently found to be co-amplified with the nearby gene CCND1 in a subset of patients with LUSC, which is associated with a poor prognosis. The simultaneous amplification and expression of FGF19 and CCND1 stimulate cell cycle progression in LUSC cells by activating FGFR4-ERK1/2 signaling, enhancing the CCND1-driven phosphorylation and inactivation of retinoblastoma. Reduced expression of CCND1 in mouse models diminishes the cell proliferation induced by FGF19 and extends survival 234 (Figure 5). The oncogenic role of FGF19 in LUSC has been partially elucidated; therefore, a combined inhibition strategy targeting both FGF19 and CCND1 may offer a promising therapeutic pathway for the treatment of this cancer.

### Other Tumors

In hepatoblastoma, FGF19 binds to the receptor, activating downstream signaling pathways. These pathways include the phosphorylation of FRS2, activation of the MAPK signaling pathway, and inhibition of Rb phosphorylation, which in turn promote cell cycle progression from G1 to S phase and drive cell proliferation. The high expression of FGF19 is closely related to the proliferative capacity of hepatoblastoma cells. Silencing the FGF19 gene using shRNA or neutralizing secreted FGF19 using anti-FGF19 antibodies can significantly inhibit the proliferation of hepatoblastoma cells, with this inhibitory effect being dose-dependent. Additionally, the use of FGF receptor kinase inhibitors, such as LY2874455, can effectively inhibit the activation of the FGF19 signaling pathway, thereby suppressing the growth of hepatoblastoma cells 235. Moreover, scRNA-seq technology has been widely employed to dissect the transcriptional heterogeneity of tumor cells in hepatoblastoma. Through the analysis of primary tumor tissue samples from patients with hepatoblastoma, gene expression patterns associated with FGF19 have been precisely identified. Relevant studies have revealed significant transcriptional heterogeneity of FGF19 in hepatoblastoma, with its expression closely correlated with the activation of the Wnt signaling pathway. Further analysis demonstrated that the expression of FGF19 was colocalized with markers of cholangiocytes, suggesting that FGF19 may regulate the proliferation of tumor cells through a cholangiocytic differentiation pathway. In addition, scRNA-seq analysis revealed significant differences in FGF19 expression levels among various tumor cell subpopulations, with these differences closely associated with the proliferative capacity of tumor cells 236. Taken together, these findings indicate the heterogeneous expression of FGF19 in hepatoblastoma and underscore its role in tumor cell proliferation. This provides a theoretical framework for the development of future targeted therapies.

In dermatofibrosarcoma protuberans (DFSP), FGF19 activates downstream signaling pathways by binding to FGFR1 and FGFR2, thereby promoting cell proliferation, survival, and migration, and ultimately driving tumor growth. This activation primarily occurs through autocrine or paracrine mechanisms. Additionally, the fusion protein formed by the gene translocation of COL1A1-PDGFB, which is commonly seen in DFSP, further enhances the activity of the FGFR signaling pathway 237. This fusion protein is capable of interacting with FGFR, leading to increased phosphorylation and thereby amplifying the downstream signaling cascade, which further promotes tumor growth and survival 238.

In glioblastoma multiforme (GBM), FGF19 can also significantly enhance the proliferation, migration, and invasiveness of GBM cells. This synergistic effect is more pronounced in recurrent tumors and is closely associated with poor prognosis. High expression of FGF19 is not only associated with increased clonogenicity and stem cell-like properties of GBM cells, but also with enhanced tumor invasiveness, mediated by alterations in cell adhesion and activation of the integrin signaling pathway via FGFR4. Combined targeting of the FGF19/FGFR4 axis and the integrin signaling pathway has the potential to offer a more effective therapeutic strategy for GBM 239. In rhabdomyosarcoma (RMS), elevated expression levels of FGF19 are typically observed compared to those in normal tissues, and this expression is positively correlated with increased tumor invasiveness and malignancy. Furthermore, high FGF19 expression is closely associated with poor prognosis in RMS patients, as evidenced by higher recurrence rates and reduced survival 240. Therefore, it can serve as a potential biomarker for the diagnosis and prognostic evaluation of RMS.

Future research ought to be directed toward several key areas. Mechanistic studies are necessary to investigate how FGF19 interacts with other pathways, such as integrin signaling in glioblastoma multiforme, and its role in tumor progression, with the potential to reveal novel therapeutic targets and inform combination strategies. The efficacy and safety of FGF19-targeted therapies, administered alone or in combination, ought to be evaluated in clinical trials, specifically in rare tumors including hepatoblastoma, DFSP GBM, and RMS. Furthermore, the validation of FGF19 as a biomarker through large-scale studies in RMS and other tumors is necessary to facilitate early detection, risk stratification, and the personalization of treatment decisions.

### Comparison across Cancer Types

FGF19 overexpression is often observed in various cancers, and its contribution to tumor progression is primarily mediated through the activation of FGFR4, although the specific mechanisms and outcomes differ among cancer types. In HCC, FGF19 is signaled through FGFR4/β-Klotho, leading to the activation of ERK1/2, which promotes cell proliferation and resistance to the drug sorafenib. In CRC, liver metastasis is enhanced by FGF19 through the upregulation of ELF4 and FGFR4. BC progression features FGF19 upregulation post-chemotherapy, especially with paclitaxel, promoting cell migration, invasion, and stress response, likely via ATF4. Whereas in OC, resistance to cisplatin is mediated by inducing autophagy via activating the p38 MAPK signaling pathway. In GC, FGFR4 expression is increased by FGF19 via the LIF-LIFR-JAK1/STAT3 pathway. The activation of the AKT/ERK1/2 pathways marks PDAC, and angiogenesis in NPC is promoted through FGFR4/ERK signaling. Metabolism and metastasis impacts in TC are mediated via β-Klotho/FGFR4 pathways, while CCA involves the activation of the PI3K/AKT pathway. The feedback loop involving GLI2 characterizes LUSC. A poor prognosis is often associated with elevated FGF19 expression in numerous studies. Understanding the varied roles of FGF19 in each cancer type is crucial for researchers, as this understanding prompts the creation of tailored therapies targeting FGF19. Given the involvement of FGFR4, the diversity of downstream pathways and outcomes necessitates cancer-specific strategies.

## FGF19/FGFR4-Targeted Therapeutic Strategies

FGF19 plays a crucial role in various cancers, with its aberrant activation closely linked to tumor initiation, progression, and metastasis 241. Aberrant activation of the FGF19/FGFR4 signaling pathway has been identified in many cancers. The oncogenic potential has led to a rigorous investigation of several FGFR inhibitors for their mechanisms of action and therapeutic potential (Table 2). These inhibitors, due to their ability to specifically block the FGF19/FGFR4 signaling pathway, can minimize interference with other FGFR family members, such as FGFR1, FGFR2, and FGFR3. However, these inhibitors also have limited efficacy, with low response rates and short response durations in trials. There are also other strategies emerging, including monoclonal antibodies such as 1A6, G1A8, HS29, U3-1784, and LD1, antibody-drug conjugates (ADCs) such as 3A11-MMAE and 3A11-Exatecan, CRISPR-Cas9 gene-editing technology, and combination therapies such as the use of FGFR4 inhibitors with PD-1 inhibitors and the combination of H3B-6527 with Palbociclib, for their mechanisms of action and therapeutic potential. These strategies have demonstrated significant antitumor effects in clinical trials and *in vitro* and *in vivo* experiments. Although potentially more effective, combination therapies remain complex and may induce adverse reactions. Patient adherence may be affected by safety concerns, including hepatotoxicity and other side effects, such as gastrointestinal and skin issues. Patient selection and treatment strategies are complicated by limitations in biomarkers, such as poor correlation between FGF19 gene amplification and protein expression. Therefore, the development of precise combination therapies should be based on a deeper understanding of the tumor microenvironment. Additionally, optimization of safety requires comprehensive preclinical toxicity studies. For biomarker discovery, advanced genomic and proteomic analyses should be employed, potentially improving patient selection and treatment outcomes. Additionally, emerging therapies such as CAR-T cell therapy and siRNA technology have also demonstrated potential. Future research should continue to explore new targets, optimize combination therapy regimens, and develop safer and more effective delivery systems to fully realize the potential of FGF19/FGFR4-targeted therapy, thereby providing more effective treatment options and better prognoses for cancer patients.

### Specific Inhibitors

#### Irpagratinib (ABSK011)

Irpagratinib (ABSK011) was developed by Abbisko Therapeutics Co., Ltd. Its structure features a pyrido-pyrimidine ring as its core, combined with substituents such as phenyl rings, piperidinyl rings, methoxy groups, and fluorine atoms. It selectively targets Cys 552, a cysteine residue within the active site of FGFR4, resulting in the irreversible inhibition of FGFR4 autophosphorylation 242. This effectively blocks the activation of the FGF19-FGFR4 signaling pathway, reducing tumor cell proliferation. Irpagratinib exhibited significant anti-tumor activity in HCC patients with FGF19 overexpression. Clinical trials of therapy have demonstrated a high overall response rate (ORR) and favorable response duration. Abbisko Therapeutics presented the Phase I study data of Irpagratinib monotherapy for advanced HCC patients at the European Society for Medical Oncology Congress in 2024. The findings indicated that in HCC patients who had previously received immune checkpoint inhibitors and multi-kinase tyrosine kinase inhibitors, and showed FGF19 overexpression, Irpagratinib achieved an ORR of 44.8%, a median response duration of 7.4 months, and a median progression-free survival of 5.5 months 243, 244. Adverse reactions were observed, including elevations in ALT and AST, as well as elevated bilirubin and alkaline phosphatase, although the incidence rates for these were unspecified. Gastrointestinal reactions were documented, including diarrhea with an incidence of more than 20%, and Grade 3/4 in over 5% of cases. Hematologic reactions comprised thrombocytopenia, with incidence remaining unspecified. Other reactions were also observed, including hyperphosphatemia and elevated total bile acid levels, with their incidence also unspecified. Irpagratinib is primarily used to treat advanced solid tumors, particularly HCC, CCA, and BC with abnormalities in the FGFR4 signaling pathway. In general, although Irpagratinib has demonstrated promise in tumor treatment, further research is needed to optimize approaches and balance its effectiveness with the management of side effects.

#### Roblitinib (FGF401)

FGF401, developed by Novartis, contains groups such as tetrahydro-naphthyridine and piperazine. Its aldehyde group is responsible for forming a reversible covalent bond with Cys552 in the kinase domain of FGFR4. The nitrogen atom of the pyridine ring is involved in forming a hydrogen bond with the N-H group of A553, and the oxygen atom of the piperazine group is involved in forming a hydrogen bond with the guanidino group of R483. This binding mode enables the overcoming of “gatekeeper mutations” in FGFR4, thereby achieving high selectivity with low off-target effects. Some results from clinical trials have been published 245. In a Phase I clinical trial involving patients with advanced solid tumors, FGF401 demonstrated certain antitumor activities in combination with pembrolizumab. In the 80 mg twice-daily dose group, an ORR of 16.7% and a disease control rate (DCR) of 50.0% were observed. The pharmacokinetic profile of the drug indicated a median time to reach peak plasma concentration (Tmax) ranging from 0.55 to 1.03 hours, with an average terminal half-life (T1/2) between 4.00 and 4.92 hours. Attributable to its capability to normalize tumor vasculature and improve the tumor microenvironment, the combination of FGF401 with vinorelbine significantly enhanced antitumor activity, as observed in a separate study. Nonetheless, several adverse effects were uncovered during clinical trials. Diarrhea (94.7%), elevated aspartate aminotransferase (AST) levels (57.9%), and elevated alanine aminotransferase (ALT) levels (47.4%) were the most frequently reported treatment-emergent adverse events (TEAEs). Proteinuria (21.1%), anemia (21.1%), and hyperphosphatemia (15.8%) were observed as additional adverse events (AEs). Dose-limiting toxicities (DLTs) were experienced by 40.0% of patients within 28 days post initial dosing, comprising 3 DLTs, in the 100 mg dosage group. Moreover, treatment interruption due to AEs occurred in 21.1% of patients. Implication of FGF401 in bile acid metabolism disorders may occur, potentially contributing to gastrointestinal adverse reactions, notably diarrhea. Additionally, the inhibitory effect of FGFR4 by FGF401 may potentially increase the risk of cholestasis injury in some patients 246. In summary, although FGF401 has demonstrated antitumor potential, and the high incidence of AEs, especially at elevated doses, necessitates further research to optimize dosing regimens and balance efficacy with safety.

#### Fisogatinib (BLU-554)

The quinazoline ring serves as the core structure of BLU-554, thereby enabling specific binding to the kinase domain of FGFR4 and inhibiting its activity 247. The presence of the tetrahydrofuran ring adds rigidity and stability to the molecule, thereby facilitating its binding to FGFR4. The acrylamide group is likely to participate in hydrogen bond interactions with FGFR4, further enhancing the binding affinity. BLU-554's selectivity is attributed to steric hindrance and the limited rotational freedom of its tetrahydro-pyran ring, which restricts binding to FGFR1-3 P-loop cysteines. FGFR4's rigid hinge region also enhances BLU-554 binding to Cys552, reducing non-target reactivity 248. BLU-554 is primarily utilized for treating HCC and other cancers. A global Phase I clinical trial involved 38 patients with FGFR4-driven HCC who had undergone multiple chemotherapy treatments; six patients (16%) achieved objective responses, 26 patients (68%) had disease control, and 18 patients (49%) experienced a reduction in tumor burden. Another Phase I trial, involving dose-escalation and dose-expansion protocols, enlisted 25 patients administered BLU-554 at doses ranging from 140 to 900 mg once daily, with the maximum tolerated dose (600 mg once daily) expanded to include 81 patients. Among FGF19-positive patients, the overall response rate was 17%, with a median duration of response (DOR) of 5.3 months 249. In terms of adverse reactions, Phase I trials primarily reported Grade 1-2 events, mainly gastrointestinal issues such as diarrhea, nausea, and vomiting. Given these potential adverse effects, implementing a robust monitoring plan is crucial for early detection and intervention, thereby reducing the severity of these effects. Furthermore, the occurrence of immune-related adverse reactions necessitates close collaboration with immunologists and specialists in managing such events. Guidance can be provided on appropriate treatments and regimen adjustments to mitigate these effects.

#### H3B-6527

H3B-6527 was developed by H3 Biomedicine Inc. Its chemical structure contains an acrylamide group, which establishes a covalent bond with Cys552 of FGFR4 through a michael addition reaction, thus inhibiting the activity of FGFR4 250. It is currently in Phase I clinical trials for the treatment of ICC and has completed Phase I clinical trials in HCC. A total of 128 patients were enrolled in the clinical trial. Of these, 90 patients diagnosed with HCC underwent treatment with H3B-6527. Among these, 48 patients were given the medication once daily, while 42 patients received the treatment twice daily. The dose range of H3B-6527 ranged from 300 to 2000 mg quaque die (QD) or from 500 to 700 mg twice a day. Among HCC patients who had received at least two prior treatments, the QD group exhibited a median OS of 10.6 months and a median progression-free survival of 4.1 months. The ORR was 16.7%, with all responses classified as partial remissions. The clinical benefit rate was 45.8%, identified as objective response plus stable disease lasting more than 17 weeks. Pharmacokinetic data revealed that the maximum plasma concentration (Cmax) and area under the curve (AUC) of H3B-6527 were observed to be lower at the 300 mg dose but comparable between 500 and 2000 mg. After oral administration of 1000 mg under fasting conditions, the time to maximum plasma concentration (Tmax) was found to be approximately 2 to 3 hours, with a terminal half-life of about 4 to 5 hours. Accumulation with QD dosing was not observed, and food intake did not significantly alter plasma exposure of H3B-6527. In terms of safety, no DLTs or TEAEs of grade 4 or 5 were reported. In the QD group, 12.5% of patients experienced grade 3 TEAEs, while 62.5% of patients reported experiencing TEAEs. The most common TEAEs included diarrhea (45.8%), fatigue (12.5%), and nausea (12.5%). The proportion of patients discontinuing treatment due to AEs stood at 8.3% 251. Overall, H3B-6527 has shown good tolerability, safety, and clinical activity in heavily pretreated HCC patients.

#### HS236

HS236, developed by Zhejiang Hisun Pharmaceutical Co., Ltd, is a domestic Class I innovative drug targeting the FGFR4. In preclinical experiments, HS236 demonstrated significant inhibitory effects on the proliferation of FGF19-positive tumor cells *in vitro* by selectively binding to the FGFR4 target in tumor cells and blocking its function. It was found to exhibit high selectivity for FGFR4, specifically inhibiting the signaling pathway mediated by FGFR4 and minimizing the impact on normal cells. On August 21, 2020, Hisun Pharmaceuticals officially initiated Phase I clinical trials for HS236 capsules to evaluate the safety and tolerability of HS236 capsules in patients with advanced solid tumors (CTR20201674). Additionally, it aims to identify the maximum tolerable dose of HS236 and establish the recommended dose for Phase II trials. Other objectives include examining the pharmacokinetics and pharmacodynamics of HS236, and providing initial insights into its efficacy against tumors, as well as how this efficacy may relate to certain biological markers. These findings will help inform the selection of participants for the next phase of clinical trials.

#### BLU-9931

BLU-9931 is classified as an FGFR4 antagonist, initially developed by Blueprint Medicines Corp. It is a highly selective and irreversible FGFR4 inhibitor with an IC50 of 3 nM, exhibiting 297, 184, and 50 times greater selectivity for FGFR1, FGFR2, and FGFR3, respectively 252. The current global development status of this drug is listed as "terminated." Despite its termination status, BLU-9931 demonstrated significant activity in both *in vitro* and *in vivo* experiments during preclinical studies. *In vitro*, BLU-9931 was observed to dose-dependently reduce the phosphorylation of the FGFR4 signaling pathway components, including FRS2, MAPK, and AKT, in MDA-MB-453 and Hep 3B cells 253. In a study, researchers utilized patient-derived organoids (PDOs) to explore the efficacy of FGFR4-targeted therapy for breast cancer, with a focus on the luminal A subtype. They established a PDO model library covering various subtypes involving luminal A, HER2, and TNBC. Immunohistochemical screening revealed strong FGFR4 positivity only in the HBC22 PDO of the luminal A subtype, while other PDOs showed low or no staining. Treatment with the FGFR4 inhibitor BLU9931 significantly inhibited HBC22 PDO proliferation, whereas the low-FGFR4-expressing HBC30 PDO did not respond significantly 254. This highlights FGFR4 as a potential therapeutic target and provides evidence for the clinical potential of BLU9931. In HCC treatment research, researchers have validated the efficacy of BLU-9931 using HCC PDOs, which effectively simulate the pathological characteristics and drug responses of HCC. In the experiment, HCC PDOs were treated with BLU-9931, and a control group was established to evaluate the impact of BLU-9931 alone and in combination with other drugs on the growth and survival of HCC PDOs. The results showed that BLU-9931 significantly inhibited the growth of HCC PDOs and reduced cell viability. When used in combination with lenvatinib, it demonstrated a stronger synergistic inhibitory effect, effectively overcoming drug resistance. Mechanistic studies revealed that BLU-9931 inhibits the FGFR4-mediated STAT3/AKT/ERK signaling pathway, reduces the levels of IL-8 and MMP-9, and induces c-PARP expression, promoting apoptosis 255. In PDAC cells, BLU-9931 was shown to inhibit the proliferation of PK-1 and T3M-4 cells, although it had no effect on PK-45P cells with low FGFR4 expression. Long-term treatment of PDAC cells with BLU-9931 resulted in morphological changes, with some cells becoming enlarged and flattened. Additionally, an increase in the number of SA-β-Gal-positive cells was noted, suggesting that BLU-9931 may potentially induce cellular senescence through DNA damage 253. In Hep 3B cells, treatment with BLU-9931 was found to increase expression of CYP7A1 mRNA and reduce the expression of the proliferation marker EGR1. *In vivo* studies using a Hep 3B tumor xenograft mouse model demonstrated that BLU-9931 dose-dependently inhibited tumor growth. At a dose of 100 mg/kg, some mice remained tumor-free even 30 days after treatment cessation. Regarding AEs, BLU-9931 was generally found to be well-tolerated, with the main drug-related AEs classified as mild and manageable. These AEs included hyperphosphatemia, nail and mucosal disorders, fatigue, and reversible retinal pigment epithelial detachment 256. Most of these AEs were reversible with temporary interruption or, in fewer cases, permanent discontinuation of treatment.

### Pan-FGFR Inhibitors

Pan-FGFR inhibitors possess the capability to inhibit the functional activities of FGFR1-4 concurrently. While promising anti-tumor efficacy has been demonstrated by these pan-FGFR inhibitors in specific tumor types, their lack of selectivity may result in an augmented spectrum of adverse effects. Sensitivity to pan-FGFR inhibitors varies among different FGFR gene variants. For instance, within the BLC2001 trial, a numerically superior response was observed in patients possessing FGFR3 mutations compared to those with FGFR2/3 fusions. This indicates that other gene variants, like FGFR2/3 fusions, may exhibit a poorer response to erdafitinib compared to FGFR3-TACC3 fusions 257. Additionally, in CRC organoids, significant growth reduction has been observed in both FGFR4-variant and wild-type CRC organoids, attributed to FGFR4-targeted drugs, such as erdafitinib and FGFR4-IN-1, suggesting that these inhibitors may lack selectivity for FGFR4 genotypes 258. Furthermore, in solid tumors, cell lines exhibiting high FGFR1/3 expression have demonstrated increased sensitivity to the FGFR inhibitor PD173074, notably in breast, liver, lung, and ovarian cancers 259. Sensitivity to pan-FGFR inhibitors can be significantly impacted by the expression levels of specific FGFR genes and the presence of certain variants. Therefore, a personalized treatment approach is crucial for effectively addressing these challenges. Comprehensive FGFR gene testing, which encompasses mutations, fusions, amplifications, and other variant types, should be conducted prior to treatment to accurately identify patients most likely to respond to specific pan-FGFR inhibitors. Moreover, dynamic monitoring of changes in FGFR gene variants throughout the treatment process is important, utilizing techniques such as liquid biopsy. If new drug-resistant mutations arise or resistance to the current treatment develops, the treatment regimen must be promptly adjusted by transitioning to more potent drugs or adopting combination therapy strategies.

#### Erdafitinib

Erdafitinib, an oral pan-FGFR inhibitor, features a unique structural framework. The core structure of Erdafitinib consists of a quinolinyl ring linked to a pyrazole ring, an essential functional group. Additionally, the methoxyphenyl group acts as a substituent, enhancing the molecule's hydrophobicity. An ethylamine chain connects the methoxyphenyl group and the quinolinyl ring, modulating the molecule's overall conformation. Efficacy in treating advanced cancer with FGFR gene alterations has been demonstrated by Erdafitinib, as evidenced by clinical trials 260, 261. In a Phase I trial enrolling 65 patients with advanced solid tumors, including 8 with urothelial cancer, an ORR of 46.2% was observed among those with pathogenic FGFR changes. The Phase II BLC2001 trial, involving 99 patients with locally advanced or metastatic urothelial cancer post-chemotherapy, reported a 40% ORR, with a partial response rate of 37% and a complete response rate of 3%. At a median follow-up of 11 months, median progression-free survival (PFS) and OS were 5.5 and 13.8 months, respectively, while final analysis at 24 months showed PFS at 5.5 months and OS at 11.3 months. Erdafitinib is currently being compared with standard treatments in the ongoing Phase III THOR trial, involving patients with advanced or unresectable urothelial cancer harboring FGFR3 mutations and/or FGFR2/3 fusions 257. However, Erdafitinib is accompanied by notable adverse reactions. Common adverse effects include hyperphosphatemia (77%), stomatitis (58%), diarrhea (51%), fatigue (28%), dry mouth (42%), blurred vision, and dry eyes. Serious adverse reactions may involve Grade 3 or higher stomatitis (14%), hyponatremia (11%), and potential retinopathy. Additionally, regular monitoring of phosphate levels is necessitated by the risk of elevation. At the same time, patients are advised to avoid pregnancy or fathering children during treatment and for one month post-discontinuation due to potential effects on fertility. Ongoing clinical trials aim to further evaluate the long-term efficacy and safety of Erdafitinib.

#### Futibatinib (TAS-120)

Futibatinib (TAS-120) is an irreversible pan-FGFR inhibitor that covalently binds to cysteine residues located in the ATP-binding sites of FGFR1-4, thereby inhibiting the FGFR signaling pathway. The molecule is characterized by a pyrazolopyrimidine core that forms hydrogen bonds with the FGFR's hinge region, including the amino acids Ala564 and Glu562. A 3,5-dimethoxyphenyl ring is present at the 3-position and occupies the FGFR's hydrophobic pocket, interacting with the amino acid Asp641. The acrylamide group is crucial as it covalently binds to the P-loop cysteine residue, thereby enabling specific and irreversible FGFR inhibition 262. This design is effective in blocking FGFR signaling and tumor cell proliferation. In the FOENIX-CCA2 Phase II clinical trial, an ORR of 41.7% and a DCR of 82.5% were demonstrated among 103 patients who had unresectable, locally advanced, or metastatic CCA harboring FGFR2 fusions or other rearrangements. The median DOR was 9.7 months. Common AEs were noted, including nail toxicity, musculoskeletal pain, constipation, diarrhea, fatigue, dry mouth, alopecia, stomatitis, and abdominal pain, as well as laboratory abnormalities such as hyperphosphatemia and increased creatinine 263. On September 30, 2022, accelerated approval was granted by the U.S. Food and Drug Administration for Futibatinib to treat patients with FGFR2-altered intrahepatic cholangiocarcinoma (iCCA). In a study involving patient-derived xenograft (PDX) models of BC with FGFR genetic abnormalities, significant antitumor effects were found, particularly in models with FGFR2 amplification and FGFR2 Y375C mutations. In the PDX.007 model, Futibatinib treatment reduced tumor volume by an average of 72%, with disease control lasting over 100 days 264. These findings suggest that FGFR2 amplification and activating mutations could serve as biomarkers for sensitivity to Futibatinib in BC patients. In a multi-tumor Phase I expansion trial involving 197 patients with advanced solid tumors, Futibatinib achieved an ORR of 13.7%, with partial responses observed across various tumor types, including CCA, BC, GC, urothelial cancer, central nervous system tumors, and head and neck cancers. Common AEs observed included hyperphosphatemia, fatigue, diarrhea, and nausea, indicating a manageable safety profile 265. Overall, Futibatinib has been shown to exhibit significant antitumor activity and acceptable safety in various cancers characterized by FGFR genetic abnormalities. This is particularly evident in CCA and certain cases of BC, thus providing a new treatment option and offering compelling evidence for future research and clinical applications.

#### AZD4547

AZD4547 was initially developed by AstraZeneca. The design of AZD4547's molecular structure facilitates a tight binding to the kinase domain of FGFR. The crystal structure, resolved at 1.65 Å, reveals its precise molecular recognition, forming a long and narrow binding groove with extensive drug-protein interactions and a network of water molecules 266. AZD4547 has been the subject of investigation in clinical trials for various types of cancer. Its use was explored in combination with aromatase inhibitors like anastrozole or letrozole in the RADICAL trial, focusing on endocrine-resistant, estrogen receptor-positive metastatic BC. After 12 weeks, among the cohort of 52 patients, partial response was achieved by 2, and 19 presented with stable disease, resulting in a clinical benefit rate of 40.4%. At 28 weeks, within the 50 evaluable patients, a partial response was achieved by 5, resulting in an objective response rate of 10% and a clinical benefit rate of 26%. The median progression-free survival was 92 days. However, discontinuation due to AEs occurred in 14 patients, and retinal pigment epithelial detachment was experienced by 11 patients, with only one case being irreversible 267. In GC, it was compared with paclitaxel in a randomized open-label study among patients characterized by FGFR2 overexpression or gene amplification. No significant differences in objective response rate, progression-free survival, or OS were observed between AZD4547 and paclitaxel groups 268. Some antitumor activity was demonstrated in a Phase Ib open-label multicenter study involving patients with advanced squamous cell carcinoma, with an overall response rate of 8% (indicating one partial response), 13.3% remaining progression-free at 12 weeks, and a median OS of 4.9 months. Gastrointestinal and dermatologic AEs were commonly observed, with grade 3 or higher events occurring in 23% of patients. A poor correlation between FGFR1 amplification and expression, along with heterogeneity in the 8p11 amplicon, was demonstrated by molecular tests, likely responsible for the modest efficacy observed with FGFR inhibition in this disease 269. Additionally, it was found to be well tolerated in a Phase I study among Japanese patients with advanced solid tumors, with no DLTs observed and no maximum tolerated dose established. Dysgeusia (50%), stomatitis (41%), diarrhea (38%), hyperphosphatemia (38%), and dry mouth (35%) were the most frequently reported AEs. Serious AEs were infrequent, occurring as grade 3 or higher nausea in 12% of patients and neutropenia in 9% 270. Overall, AZD4547 is indicated to provide some clinical benefits, particularly in endocrine-resistant BC cases, though its efficacy in GC and NSCLC appears limited. The optimization of its safety profile, particularly in addressing retinal adverse reactions, remains necessary.

### Monoclonal Antibody

#### Anti-FGF19 Monoclonal Antibody

Monoclonal antibody 1A6 is an IgG1 subtype targeted therapeutic agent that specifically binds to the C-terminus of FGF19, thereby blocking the interaction between FGF19 and FGFR4 and inhibiting FGF19-mediated signaling. This inhibition has significant antitumor effects, as demonstrated in both CRC xenograft and FGF19 transgenic HCC models. In CRC xenograft studies, treatment with 1A6 at different doses resulted in tumor growth inhibition rates of 57% and 60%, respectively. Within the FGF19 transgenic HCC model, 1A6 treatment significantly reduced HCC incidence, with treated mice exhibiting minimal liver tumor formation, a substantial decrease in liver weight, and a reduction in tumor volume. Micro-CT analysis further revealed that the proportion of tumor volume to total liver volume was significantly lower in treated mice compared to controls 271. Moreover, an *in vivo* experiment evaluated the anti-tumor effect of the FGF19-neutralizing antibody 1A6 in a mouse model of 11q13.3-amplified HCC using Huh-7 cells. Mice bearing 0.2 cm³ tumors were administered PBS, a control antibody, or 1A6. While most PBS and control-treated mice were sacrificed due to tumor burden, 1A6 significantly inhibited tumor growth, demonstrating its therapeutic potential for HCC 73. Although the specific targeting of FGF19 by 1A6 has been shown to effectively inhibit tumor growth and highlight the therapeutic potential of FGF19 for treating CRC and HCC, there remains the possibility of non-specific binding to other proteins, resulting in unintended signaling or immune reactions. Nevertheless, monoclonal antibodies targeting FGF19 have been reported to induce dose-dependent hepatotoxicity, which is accompanied by severe diarrhea and reduced food intake 272. In a therapeutic study involving 1A6 in monkeys, significant increases in serum total bile acids, ALT, and AST levels were observed. The 1A6 antibody was also found to disrupt the normal bile acid regulatory function of FGF19, resulting in severe bile acid-related side effects 273.

G1A8 and HS29 are newly developed humanized monoclonal antibodies that specifically target the N-terminus of FGF19. Through phage display technology and antibody engineering, these antibodies can inhibit FGF19-induced HCC cell proliferation without affecting the normal bile acid regulatory function of FGF19. G1A8 and HS29 significantly inhibited FGF19-induced HCC cell proliferation, with G1A8 showing an inhibitory effect comparable to that of 1A6, while HS29 exhibited superior physicochemical properties. In mouse models, G1A8 and HS29 significantly inhibited the growth of HCC tumors and prolonged the survival of mice. Moreover, HS29 demonstrated antitumor activity in PDX models expressing FGF19, but not in FGF19-negative HCC models. Safety assessments indicated that G1A8 treatment in cynomolgus monkeys did not cause any side effects related to bile acid metabolism, such as weight loss, decreased appetite, or diarrhea. There were no observed increases in CYP7A1 gene expression or significant changes in serum total bile acid, total bilirubin, ALT, AST levels, indicating that G1A8 does not cause liver damage or significant disruption of bile acid metabolism 274. Although G1A8 and HS29 have shown good antitumor activity and safety, there is still room for further optimization. For example, efforts could be made to enhance the antibodies' affinity, stability, and pharmacokinetic properties to improve therapeutic efficacy and reduce potential side effects.

#### Anti-FGFR4 Monoclonal Antibody

Anti-FGFR4 mAbs are emerging as a targeted therapy for RMS. These mAbs bind specifically to FGFR4 on tumor cells, blocking signaling pathways, inducing receptor internalization, and enhancing therapeutic efficacy through ADCs 240. Showing promise in RMS treatment, these mAbs also hold potential for other cancers with high FGFR4 expression, such as prostate, melanoma, lung, breast, colorectal, and gastric cancers 275. Optimizing the development of these antibodies and evaluating their efficacy and safety in preclinical and clinical settings should be a focus in the future. Below are several anti-FGFR4 mAbs that are being studied for the treatment of other types of tumors.

##### U3-1784

U3-1784 is produced through phage display technology as a fully human antibody with high affinity. It specifically targets FGFR4, thereby inhibiting its activation by competing with various FGFs for the binding site. As a result, downstream signaling pathways such as FRS2 and Erk214 are interfered with 276. *In vitro* experiments have shown that U3-1784 exhibits an extremely high affinity for FGFR4, with a Kd of approximately 0.3 nmol/L, while no significant binding to FGFR1-3 is observed, demonstrating good specificity. An effective competitive inhibition of FGF19 binding to FGFR4 with an IC50 value of 2 nmol/L is demonstrated, and inhibition of FGFR4 phosphorylation and its downstream signaling pathways is achieved. *In vivo* experiments have demonstrated that U3-1784 significantly inhibits tumor growth in ten different HCC models with overexpressed FGF19, achieving an inhibition rate of up to 90%. However, it has no significant effect on tumor models that do not express FGF19. The use of U3-1784 alone can lead to increased levels of serum bile acids and liver enzymes in rhesus monkeys, indicating potential liver damage. Nevertheless, no significant effect is observed on tumor models lacking FGF19 expression. The administration of U3-1784 alone can lead to increased levels of serum bile acids and liver enzymes in rhesus monkeys, indicating potential liver damage. However, this toxicity may be mitigated by administering oral bile acid chelators. U3-1784 exhibits advantages such as high specificity and affinity, significant antitumor effects, potential for combination therapy, and manageable toxicity. Nonetheless, certain disadvantages, including potential hepatotoxicity and limitations of preclinical research, are present. Optimization of the molecular structure of U3-1784 may be required. Modification or engineering of certain amino acid residues in the antibody through medicinal chemistry could potentially reduce hepatotoxicity and improve the drug's safety profile. When combined with sorafenib, enhanced antitumor efficacy is achieved by U3-1784 without additional toxicity, offering a promising avenue for combination therapy 277. The utilization of U3-1784 alongside other targeted therapies, immunotherapies, or chemotherapeutic agents may enhance therapeutic outcomes and reduce drug toxicity. Although clinical trials (NCT02690350) for HCC and other advanced solid tumors have been discontinued, the remarkable specificity and antitumor activity observed in preclinical tests suggest that the potential of U3-1784 in cancer treatment may warrant future reconsideration. Potential facilitation of this reevaluation could be achieved by further optimization or novel clinical trial designs.

##### LD1

The variable region of LD1 is designed to precisely recognize and bind to glycine at position 165 (G165) on FGFR4, a critical site for the dimerization and subsequent activation of FGFR4. The constant region of LD1 is based on the human IgG1 framework, which enhances the stability of the antibody and extends its half-life *in vivo*, thereby improving therapeutic efficacy and convenience 278.

LD1 exerts anti-tumor effects through multiple mechanisms. It can effectively block the binding of FGF1 and FGF19 to FGFR4, thereby inhibiting the activation of FGFR4. This blockage leads to the suppression of downstream signaling pathways, particularly the phosphorylation of ERK1/2 and FRS2, which in turn significantly reduces the proliferation and colony formation capabilities of HCC cell lines. *In vitro* experiments have shown that LD1 can inhibit up to 82% of colony formation in the PLC/PRF/5 cell line. *In vivo*, LD1 demonstrates remarkable anti-tumor effects. In the HUH7 liver cancer xenograft model, a weekly dose of 30 mg/kg of LD1 inhibits 96% of tumor growth. Similarly, in the FGF19 transgenic mouse model, LD1 significantly reduces liver weight, effectively preventing the development of liver cancer. Beyond direct tumor growth inhibition, LD1 also regulates gene expression by upregulating CYP7A1 and downregulating c-Fos (FOS), genes closely related to FGFR4 activity 278. In another animal model, chLD1, a chimeric mouse antibody, significantly reduced tumor growth in the HUH7 human HCC xenograft mouse model, with an average tumor volume reduction of approximately 43% compared to the PBS-treated group. However, hLD1.vB, a humanized antibody, exhibited inferior antitumor efficacy, with only a 14% reduction in tumor volume, despite having similar binding affinity to FGFR4 as chLD1. This discrepancy was primarily attributed to the rapid clearance of hLD1.vB in mice, which limited its distribution to target tissues. Affinity maturation led to the development of hLD1.v22, an optimized variant that eliminates C3 binding. hLD1.v22 demonstrates improved pharmacokinetics and tumor inhibition, with a 75% reduction in tumor volume, matching the efficacy of chLD1 279.

These findings further confirm that LD1 inhibits the FGFR4 signaling pathway via multiple pathways. As a monoclonal antibody targeting FGFR4, LD1 has demonstrated high efficiency and specificity in treating liver cancer, both suppressing proliferation and preventing tumor development, which suggests its potential for early intervention strategies. Nevertheless, rapid clearance and suboptimal tissue distribution of hLD1.vB emphasizes the importance of eliminating nonspecific interactions, such as unintended binding to complement proteins, which can negatively impact pharmacokinetics and efficacy. Although humanized to reduce immunogenicity, LD1 may still trigger ADA production in patients, compromising efficacy and causing adverse effects. While hLD1.v22 avoids binding to mouse C3, similar non-specific interactions in other species or humans could lead to inflammation and tissue damage 279.

Future research should focus on further optimizing antibody design to minimize non-specific interactions and enhance *in vivo* stability, leveraging advanced screening methods and *in silico* modeling to predict and avert off-target effects. Comprehensive clinical testing in diverse animal and humanized models is essential for assessing immunogenicity, pharmacodynamics, and tissue distribution. These strategies will be crucial for the development of safer and more efficacious FGFR4-targeted antibodies for clinical use. Additionally, exploring the utility of LD1 in other FGFR4-driven malignancies and evaluating its safety and efficacy in clinical trials remain important directions for future investigation.

### Antibody-drug Conjugates

ADCs represent a novel therapeutic approach that leverages the specificity of monoclonal antibodies to selectively deliver cytotoxic drugs to cancer cells, thereby minimizing damage to healthy tissues. Two FGFR4-targeted ADCs, 3A11-monomethyl auristatin E (MMAE) and 3A11-Exatecan, have been developed to exploit this strategy. 3A11-MMAE is conjugated to MMAE via a cathepsin-cleavable linker, inducing apoptosis in FGFR4-positive cells. Conversely, 3A11-Exatecan utilizes a legumain-cleavable linker to conjugate an exatecan derivative, resulting in cancer cell death primarily through DNA damage.

Experimental studies have shown that the 3A11 antibody is efficiently internalized by FGFR4-positive cells, with internalization rates correlating with the surface expression levels of FGFR4. ADCs based on 3A11 selectively kill FGFR4-expressing cells *in vitro*, with cytotoxic efficacy proportional to both target expression and antibody uptake. It has been confirmed that 3A11-MMAE induces specific apoptosis in FGFR4-positive RMS cells, while 3A11-Exatecan induces cell death following DNA damage. To enhance the therapeutic potential of 3A11, its variable light and heavy domains were cloned to create a chimeric 3A11 scFvFc structure (mouse Fv and human IgG1 Fc), which was successfully expressed *in vitro* and maintained its specificity 280. *In vitro* cytotoxicity experiments revealed that co-culturing the FGFR4-expressing RH30 RMS cell line with chimeric 3A11 and duocarmycin DM-conjugated polyclonal anti-mouse 2° ADC for 72 hours resulted in dose-dependent cytotoxicity, indicating that chimeric 3A11 can effectively deliver cytotoxic payloads to FGFR4-positive cells. *In vivo* experiments in subcutaneous xenograft models further validated the therapeutic efficacy of these ADCs. In a fusion-positive RMS (RH4) model, 3A11-MMAE significantly delayed tumor progression and extended survival by 30% at 3 mg/kg dosing. In the fusion-negative, FGFR4 V550L-mutant RMS559 model, 40% of mice achieved complete tumor remission and 70% overall survival. Notably, 3A11-Exatecan was even more potent, achieving complete tumor eradication and 100% survival in RH4 mice with a single dose and clearing all tumors in RMS559 mice following two doses. Both ADCs also strongly inhibited the growth of FGFR4-positive MDA-MB-453 breast cancer xenografts 281, 282.

In RMS, FGFR4 is highly expressed and regulated by PAX3-FOXO1 and PAX3/PAX7, making it a key molecule in this aggressive pediatric cancer 280. Overexpression of FGFR4 has also been observed in HCC and BC, further highlighting its potential as a target for immunotherapy. The collective experimental evidence summarized above underscores the significant antitumor potential of FGFR4-targeted ADCs in both *in vitro* and *in vivo* models. These ADCs may provide a selective approach to delivering cytotoxic agents specifically to FGFR4-positive tumor cells, thereby reducing off-target toxicity and offering a promising immunotherapeutic strategy for the treatment of RMS, HCC, and BC. Future work should focus on humanizing the lead antibody (3A11) to mitigate immunogenicity. Additionally, the design of initial clinical trials will be crucial to establish the safety, tolerability, and preliminary efficacy of FGFR4-targeted ADCs in humans. Moreover, combination strategies with immune checkpoint inhibitors or other targeted agents may be explored to enhance therapeutic efficacy further.

### Application of CRISPR-Cas9 Technology in FGF19/FGFR4 Targeted Therapy

CRISPR-Cas9 is a revolutionary gene-editing tool derived from the bacterial adaptive immune system. Through the design of specific guide RNAs, this system enables precise targeting and modification of genes 283. Owing to its high efficiency, specificity, and operational simplicity, CRISPR-Cas9 has become widely employed in basic research, disease models, and the development of novel biotechnological therapies.

In the field of cancer research and treatment, CRISPR-Cas9 provides new avenues for targeted therapies, exemplified by studies on the FGF19/FGFR4 signaling axis. Abnormal activation of the FGF19/FGFR4 pathway is closely linked to poor prognosis and increased malignancy in cancers such as HCC and HNSCC. Functional genomics studies have leveraged CRISPR-Cas9 to knock out FGFR4, resulting in enhanced sensitivity of HCC cells to sorafenib-induced ROS generation and apoptosis, a phenotype also observed with FGF19 knockout 284. In HNSCC, FGF19 gene knockout can reduce the expression of the Vimentin protein, inhibit cell migration, and enhance the inhibitory effect of melatonin on cell migration 20. Additionally, it has been discovered that the KLB gene plays a crucial role in FGF19 signal transduction. KLB can bind to FGFR3 and FGFR4, mediating the pro-survival function of FGF19. Knocking out the KLB gene using CRISPR-Cas9 technology can significantly inhibit the growth and tumor formation of HCC cells, as well as reduce the incidence of lymph node metastasis in a mouse model. Whole-genome CRISPR-Cas9 loss-of-function screening has revealed that FGFR3 limits the activity of FGFR4-selective inhibitors in inducing cell death, whereas the pan-FGFR inhibitor erdafitinib exhibits stronger efficacy than FGFR4-selective inhibitors in inhibiting the growth and survival of FGF19-positive HCC cells 285. Through CRISPR-Cas9 screening and *in vivo* and *in vitro* experiments, the study also revealed the synthetic lethal interaction between FGFR3 and FGFR4 in FGF19-positive HCC. The study found that the HuH-7 and JHH-7 cell lines had high dependency on FGFR4 and KLB, but low dependency on FGF19, suggesting that FGF19 may influence the CRISPR screening results through paracrine action. The experiments showed that the KLB knockout had a stronger inhibitory effect on cell growth and tumor growth than the FGFR4 knockout. In addition, FGFR3 knockout significantly enhanced the antitumor activity of FGFR4 inhibitors, and pan-FGFR inhibitors, such as erdafitinib, demonstrated stronger efficacy. Further research found that dual knockout of FGFR3 and FGFR4 led to a 607-fold depletion of cells in HuH-7 tumors and a 5.8-fold depletion in JHH-7 tumors, indicating functional redundancy between the two, which may be a key factor limiting the efficacy of FGFR4 inhibitors 285. These findings highlight new strategies for drug combination approaches and underscore the importance of considering FGFR family redundancy in therapeutic design.

These research results not only reveal the critical role of the FGF19/FGFR4 signaling pathway in cancer drug resistance but also provide a theoretical basis for the development of more effective targeted therapeutic strategies. In the future, combining CRISPR-Cas9 technology with drug therapy is expected to bring breakthroughs in the treatment of HCC, HNSCC, and other tumors. Despite these advances, several challenges hinder the clinical application of CRISPR-Cas9 in FGF19/FGFR4-targeted cancer therapy, including off-target effects, low *in vivo* delivery efficiency, immunogenicity, uncertain DNA repair mechanisms, and low homology-directed repair efficiency in non-dividing cells 286-288. Continued research and technological innovation are crucial for addressing these limitations and unlocking the full potential of CRISPR-based strategies in oncology.

### Combination Therapies

Although these inhibitors have demonstrated efficacy in clinical trials, the problem of resistance persists as a significant challenge for FGFR4 inhibitors. Acquired resistance to FGFR4 inhibitors occurs when cancer cells adapt to their presence. For instance, mutations in the FGFR4 gene, such as V550M, V550L, and C552G, alter the drug-binding site and reduce the binding affinity of inhibitors. FGFR4 gene amplification increases the expression of the FGFR4 protein, thereby hindering effective inhibition 289. Resistance may be developed by tumor cells through the induction of structural changes in the FGFR4 protein, especially within the ATP-binding pocket, which prevents accurate inhibitor binding 290. Modifications in FGFR4 protein degradation can also be made, such as reducing its degradation rate, which leads to intracellular accumulation and thus maintains FGFR4 signaling pathway activity. Furthermore, tumor cells can activate other FGFR family members or alternative signaling pathways to bypass the effects of the FGFR4 inhibitor, allowing cancer cell proliferation to continue even in the presence of the inhibitor 291.

However, the efficacy of inhibitors may be further enhanced by combination therapy strategies. The combination of FGFR4 inhibitors with immune checkpoint inhibitors, such as anti-programmed cell death protein 1(PD-1) antibodies, can significantly increase sensitivity to immunotherapy 292. When administered in conjunction with the anti-PD-1 antibody spartalizumab, FGF401 has shown promising safety and initial clinical effectiveness in patients with FGFR4/KLB-positive tumors. Additionally, lenvatinib, a multi-kinase inhibitor, can lower PD-L1 levels in tumors, thereby enhancing the efficacy of anti-PD-1 treatments. Pembrolizumab is a humanized monoclonal antibody that significantly improves the antitumor activity of T cells by blocking the binding of PD-1 to its ligands PD-L1 and PD-L2. The FGFR4 gene is part of an 8-gene mTOR signature, correlating with increased PD-1/L1 expression and indicating potential for predicted improved survival outcomes in patients treated with immune checkpoint inhibitors across various cancer types 293. The combination of Futibatinib and Pembrolizumab is attracting widespread attention as a treatment for advanced or metastatic HCC. Currently, a key clinical trial (NCT04828486) is underway. This is an open-label, single-arm Phase II study focusing on evaluating the efficacy and safety of Futibatinib in combination with Pembrolizumab for the treatment of advanced or metastatic HCC that is FGF19-positive and classified as BCLC Stage A, B, or C. The trial takes 6-month PFS as the primary endpoint, and plans to recruit 25 patients. The core of this combination therapy strategy lies in simultaneously inhibiting the FGFR signaling pathway and enhancing the immune response to improve treatment efficacy, bringing new hope to patients with advanced or metastatic HCC. The complete clinical results have not yet been published. However, in patients with metastatic urothelial carcinoma (mUC) unsuitable for or refusing platinum-based chemotherapy (NCT04601857), their combination therapy achieved an ORR of 47.1% in those with FGFR3 mutations or FGFR1-4 fusions/rearrangements, with a median PFS of 8.3 months and a median DOR of 12.3 months. In wild-type or other non-FGFR abnormal tumors, the ORR was 26.9% 294. In esophageal carcinoma patients, the study included both chemotherapy and ICI-naive and previously treated individuals (NCT05945823). The first-line cohort (chemotherapy and ICI-naive) had an ORR of 68.4%, a DCR of 89.5%, and a median DOR of 5.6 months. The second-line and beyond ICI-naive cohort had an ORR of 42.9%, a DCR of 71.4%, and a median DOR of 16.0 months. Additionally, Futibatinib monotherapy increased CD3+CD8+ T cells in the tumor microenvironment, with a more pronounced effect observed after 21 days of combination therapy 295. Regarding safety, 94.1% and 100% of patients in Cohort A and Cohort B, respectively, experienced treatment-related adverse events (TRAEs), with grade 3 TRAEs occurring in 41.2% and 42.3%, respectively. In patients with esophageal carcinoma, common TRAEs included hyperphosphatemia (up to 91.7%), neutropenia, stomatitis, diarrhea, and nail disorders; however, overall tolerability was good, with only one case of grade 3 stomatitis reported. These results indicate that the combination of Futibatinib and Pembrolizumab may hold potential therapeutic value and manageable safety in specific patients with FGFR-abnormal mUC and esophageal carcinoma. Future research should explore its application in these populations and its long-term impact on the tumor immune microenvironment. Besides, in a phase 1/1b, open-label, dose-escalation study (NCT03547037) of the PD-1 inhibitor cetrelimab (JNJ-63723283) in combination with erdafitinib for the treatment of advanced solid cancers, 13 patients received a combination of 240 mg cetrelimab and 6 mg erdafitinib. One patient experienced a grade 3 Stevens-Johnson syndrome, which was identified as an immune-related DLT. All patients encountered at least one TEAE, with 53.8% experiencing grade 3 or higher TEAEs, 38.5% reporting severe TEAEs, and 15.4% discontinuing treatment due to TEAEs. The exposure of cetrelimab was unaffected by the addition of erdafitinib, as evidenced by comparable trough concentrations of cetrelimab when used alone versus in combination. Cetrelimab achieved 100% occupancy of PD-1 receptors on circulating T cells within 2 hours of administration, which was maintained across all dose levels. One patient had detectable anti-cetrelimab antibodies at baseline, but no treatment-induced antibodies were detected during the study. Among the 12 evaluable patients, the ORR was 16.7%, with two patients achieving confirmed partial responses. The median DOR was 2.73 months, and the median PFS was 2.76 months 296.

Furthermore, the synergistic combination of BLU-554 and CS1001 can address HCC through dual pathway targeting. CS1001, an anti-PD-L1 monoclonal antibody, inhibits the interaction between PD-L1 and PD-1, thereby restoring T-cell function and bolstering the immune response within the tumor microenvironment. In a Phase Ib/II trial (NCT04194801) assessing the combination of BLU-554 and the anti-PD-L1 monoclonal antibody CS1001 for the treatment of locally advanced or metastatic HCC, 4 FGF19-positive patients were administered BLU-554 (600 mg once daily) alongside CS1001 (1200 mg every three weeks), achieving an ORR of 50% and a DCR of 100%. This dual mechanism is beneficial not only for halting tumor progression but also for enhancing the tumor microenvironment. Generally well-tolerated, the therapy's most frequent adverse effects included diarrhea, liver dysfunction, and skin rashes, while only one patient experienced immune-related AEs 12. In addition, the Phase II clinical trial (NCT05441475) results for ABSK011 in combination with the anti-PD-L1 antibody Atezolizumab for advanced HCC patients were presented at the 2024 ESMO-GI Congress by Abbisko Therapeutics. The combination therapy demonstrated an ORR of 50% (5/10) and exhibited considerable efficacy and safety in patients who had previously received ICIs. These observations suggest that PD-1-involving combination therapies may have broad potential applications in treating various types of cancer. Another Phase 2 clinical trial (NCT07010497) is currently recruiting to test the safety and efficacy of Irapragratinib in Combination with Atezolizumab and Bevacizumab for HCC with FGF19 overexpression. In addition, in a Phase Ib clinical trial (NCT03238196), the combination therapy of Erdafitinib, Fulvestrant, and Palbociclib was assessed for its safety, tolerability, and efficacy in 35 postmenopausal women with HR+/HER2- metastatic breast cancer, 29 of whom had FGFR1-4 -amplified tumors. The maximum tolerated dose of Erdafitinib was 6 mg daily. Results showed a 10% partial response rate, 55% stable disease, 23% clinical benefit rate at 6 months, and a median progression-free survival of 12 weeks. High FGFR1 protein expression correlated with longer progression-free survival, though FGFR1 amplification did not predict response. Adverse events included neutropenia, oral mucositis, hyperphosphatemia, and ocular toxicity, leading to dose adjustments or discontinuation in some cases 297. The triplet regimen was tolerable but did not outperform prior trials of FGFR inhibitors. High FGFR1 protein expression may inform future treatment strategies.

Additionally, H3B-6527, when combined with Palbociclib, a CDK4/6 inhibitor, effectively induces tumor regression in HCC xenograft models. Palbociclib arrests the cell cycle by inhibiting CDK4/6 and preventing Rb protein phosphorylation, while H3B-6527 targets the FGFR4 pathway, crucial to the growth of HCC 298. Together, they exhibit an enhanced anti-tumor effect. Another study involving HCC cell lines and PDX models demonstrated that FGF19 expression predicted H3B-6527 efficacy, and this combination significantly reduced tumor size 250. This therapy not only enhances efficacy and overcomes resistance but also offers new options for HCC patients unresponsive to traditional treatments. In addition to the promising combination of H3B-6527 and Palbociclib, another notable approach in the treatment of HCC involves the combination of FGF401 and infigratinib, which has also demonstrated significant antitumor effects in HCC PDX models. The combination of FGF401 and infigratinib has shown significant antitumor effects in HCC PDX models in the treatment of HCC. This combined therapy works by inhibiting the FGFR2/3 and FGF19/FGFR4 signaling pathways, thereby curbing tumor cell proliferation and inducing apoptosis. It also reduces the levels of various proteins, such as p-p70S6K/4EBP1/S6R, p-Cdk2, p-Cdc2, CDC25C, p-Cdc2, Cyclin D1, c-Myc, and survivin, while upregulating p27, the dephosphorylated form of Bim, and cleaved caspase 3, thus causing G1 cell cycle arrest and apoptosis. Moreover, the combined treatment lowers the metastatic potential of tumors by inhibiting the ERK, c-met, Wnt/β-catenin, and HDAC1-3 pathways, as well as the p70S6K pathway. In terms of the tumor microenvironment, the combined treatment normalizes blood vessels and reduces tumor hypoxia, thereby transforming the immunosuppressive microenvironment into an immune-supportive one. This results in a significant increase in the infiltration of immune effector cells into the tumor, leading to superior tumor regression compared to monotherapy. This combined treatment not only enhances antitumor activity but also reduces the effective dose of each drug. It should be noted that it is currently unclear whether this combined treatment will cause cholestasis in a clinical setting, which may have an impact on patients' liver function 299.

Additionally, studies have indicated that the activation of the EGFR signaling pathway might serve as one of the main mechanisms of resistance to FGFR4 inhibitors. Blocking the EGFR circumvents resistance to FGFR4 inhibitors and amplifies their therapeutic benefits in treating HCC 300. FGFR4 amplifies the cancer-promoting signaling of EGFR in lung adenocarcinoma tissues, with notable effectiveness being observed when both pathways are simultaneously inhibited 301. Regulation of EGFR and FGFR4 facilitated by SORL1 promotes chemoresistance in ovarian cancer 302. Therefore, the development of combination therapy regimens targeting both the FGF19/FGFR4 and EGFR pathways may provide an effective strategy for overcoming resistance in the future. Establishing organoid models carrying FGF19/FGFR4 mutations can also be used to evaluate the antitumor effects of relevant inhibitors and provide insights into the role of the FGF19/FGFR4 pathway in tumor resistance mechanisms, thereby offering new ideas and strategies for overcoming drug resistance.

Nanomedicine also holds great promise for cancer treatment. As nanotechnology advances, nanoparticles can deliver drugs more precisely to tumors, thereby increasing drug accumulation while reducing toxicity to normal tissues 303. They can carry chemotherapy, targeted therapies, or immunotherapies and can be designed to deliver FGFR4 inhibitors, which block FGF19-FGFR4 binding and inhibit tumor growth in the future. Nanoparticles may also deliver immune-modulating drugs to counteract FGF19-induced immune suppression and boost the efficacy of immunotherapy. Combining nanomedicine with FGF19-targeted therapy and delivering multiple drugs via nanoparticles will enable multi-targeted synergistic treatment, significantly enhancing cancer treatment outcomes. This approach offers a comprehensive and precise solution, bringing more hope and a better prognosis for cancer patients.

### Other Therapies

Chimeric antigen receptor (CAR)-T cell therapy is an advanced cancer immunotherapy that involves extracting T cells from a patient, genetically engineering them to express CARs that enable the T cells to specifically recognize and attack cancer cells, and then reinfusing the modified T cells back into the patient to enhance the body's antitumor immune response 304. The CAR-T cell therapy targeting FGF19/FGFR4 is currently in the preclinical development stage. FGFR4 is overexpressed in pediatric RMS due to the downstream effect of the PAX3-FOXO1 fusion protein. However, resistance mutations such as N535K and V550L often occur before treatment, which may limit the effectiveness of small-molecule inhibitors. Researchers have developed CAR-T cells targeting FGFR4, such as RJ154-HL and 3A11, which use different single-chain variable fragments to target FGFR4 305. The 3A11 CAR-T cells have demonstrated robust cytokine secretion and cytotoxicity against RMS cell lines *in vitro*, without recognition and killing of healthy human primary cells, confirming their selectivity for tumor cells with high FGFR4 expression. *In vivo* experiments have shown that FGFR4 CAR-T cells exhibit an effective response in metastatic RMS models, but not in RMS orthotopic models 306. However, when combined with pharmacological inhibition of myeloid components in the tumor stroma, FGFR4 CAR-T cells can eliminate orthotopic RMS tumors in mouse models. To address the heterogeneous expression of tumor antigens, researchers have developed bispecific CAR-T cells that target both FGFR4 and CD276. These dual-target CAR-T cells have demonstrated more potent antitumor activity in both *in vitro* and *in vivo* models 307. In particular, the FGFR4.28HTM.28z-CD276.8HTM.BBz BiCisCAR T cells have demonstrated faster tumor clearance, higher persistence, and lower expression of exhaustion markers in RMS models. To enhance the safety of CAR-T cell therapy, researchers have introduced an inducible caspase-9 suicide gene system. This system can induce apoptosis of CAR-T cells when they become overactivated, thereby reducing severe adverse reactions such as cytokine release syndrome 308. In preclinical studies, FGFR4 CAR-T cells have shown manageable safety characteristics in mouse models. However, since mouse models cannot fully simulate the adverse reactions of CAR-T cells in humans, further evaluation of the on-target/off-target toxicity of FGFR4 CAR-T cells is needed in humanized immune system mouse models or small-scale clinical trials 306. Besides, CAR-T cell therapy can cause severe side effects, and its high cost and complex manufacturing process limit its widespread use. Ongoing research should mitigate these challenges by developing safer, more cost-effective, and streamlined CAR-T cell therapies for broader application.

Small interfering RNA (siRNA) is a double-stranded RNA molecule that can specifically bind to and degrade target mRNA, thereby inhibiting the expression of specific genes. This gene silencing technology holds great potential in cancer therapy because it can precisely target key genes in cancer cells, block their signaling pathways, and inhibit tumor growth and metastasis 309. In BC research, transfection with specific siRNA to silence the FGF19 gene can effectively block the signaling between FGF19 and FGFR4, inhibiting the proliferation and migration of tumor cells 46. In TC cells, transfection with siRNA-FGF19 can significantly inhibit the expression of FGF19, thereby suppressing the migration and invasion of thyroid cancer cells 22. In OC research, targeting FGFR4 with siRNA and the FGFR4 trap protein can significantly inhibit the progression of ovarian cancer 310. In NPC cells, downregulation of FGFR4 expression by anti-FGFR4 siRNA can significantly inhibit the proliferation and migration of NPC cells 311. In gastric cancer cells, silencing FGFR4 through the use of FGFR4-siRNA has the potential to influence the biological properties 312. In CRC cells, silencing FGFR4 with siRNA technology can significantly inhibit cell proliferation, migration, and invasion, and reverse the EMT process 313. In addition, small hairpin RNA (shRNA) functions as a molecular tool capable of specifically silencing the expression of target genes. It achieves this by binding to the target mRNA and facilitating its degradation, thereby inhibiting the expression of specific genes. Within the realm of cancer treatment research, shRNA has been extensively utilized to target and silence crucial genes in cancer cells, aiming to curb tumor growth and metastasis. Notably, shRNA that targets FGFR4 has demonstrated significant efficacy in inhibiting the proliferation and metastasis of GC, PCa, and CRC cells. Additionally, it has effectively suppressed the growth of colon cancer xenografts 314. These research findings indicate that siRNA and shRNA technology have broad application prospects in various types of cancer and are expected to become an important means of cancer therapy in the future. However, both siRNA and shRNA therapies face limitations, including off-target effects, delivery challenges, and potential immune responses, which hinder their widespread clinical application. Nevertheless, the integration of nanotechnology holds promise for enhancing their delivery efficiency and specificity, potentially overcoming these hurdles 315.

Furthermore, as previously mentioned, FXR agonists and antagonists play a crucial role in regulating bile acid metabolism and the gut microbiota, thereby influencing the development of FGF19-related cancers. Natural FXR agonists, such as OCA, as well as natural antagonists like DCA, may affect the composition and function of the gut microbiota, which in turn modulate the synthesis and metabolism of bile acids. For example, the gut microbiota can influence the deconjugation of bile acids, thereby affecting the *de novo* synthesis of bile acids in the liver. This process relies on the FXR-FGF15/19 axis. In FGF19-related cancers involving HCC and CRC, the use of FXR agonists or antagonists may exert anticancer effects by modulating bile acid metabolism and the gut microbiota 81, 91. Moreover, modulation of the gut microbiota may also improve cancer treatment outcomes by influencing the FXR-FGF19 signaling pathway. Therefore, FXR agonists and antagonists, as well as modulation of the gut microbiota, may offer novel strategies for treating FGF19-related cancers. Additionally, Five Prime Therapeutics has developed an FGFR4 fusion trap protein, named FTP-091. It is composed of the three Ig-like extracellular domains of FGFR4. By replacing the acid box linker region, it gains high affinity for ligands such as FGF17 and FGF18. This fusion protein can capture ligands like FGF19, thereby inhibiting the growth signals mediated by the FGF1/FGFR4 pathway 316. However, its specificity is limited because ligands such as FGF1 can also bind to other members of the FGFR family. Future research should prioritize improving the specificity of fusion trap proteins such as FTP-091 to reduce unintended binding and enhance therapeutic outcomes.

## Conclusion and Future Perspectives

The multifaceted roles of FGF19 in cancer progression underscore its importance as a therapeutic target. Through its interactions with FGFR4 and β-Klotho, FGF19 modulates critical physiological processes, including bile acid, glucose, and lipid metabolism 317. These mechanisms are co-opted in cancer to promote tumor proliferation, invasion, metastasis, and treatment resistance. The interaction between FGF19 and FXR is vital for maintaining bile acid balance. Disruptions in this interaction can cause bile acid metabolic disturbances, which in turn drive cancer development. Overexpression of FGF19 has been associated with poor prognosis in various cancers, underlining its potential as a therapeutic target 47. The clinical research progress involving FGF19/FGFR4 inhibitors has been promising, with several specific inhibitors and pan-inhibitors showing significant anti-tumor effects in preclinical and clinical settings. However, challenges such as side effects, drug toxicity, and the development of resistance necessitate further optimization 318. The use of CRISPR-Cas9 technology and ADCs offers innovative approaches to overcoming these challenges and enhancing therapeutic precision 280, 284. CAR-T, siRNA, and shRNA have also shown great promise in therapeutic applications, paving the way for more effective cancer therapies.

The future of FGF19 research in cancer appears both promising and demanding. A deeper understanding of the molecular mechanisms underlying FGF19's oncogenic functions, particularly its interactions with the tumor microenvironment and other signaling pathways, is essential. In the realm of tumor metabolism, FGF19's capacity to regulate key metabolic pathways, including glycolysis, fatty acid synthesis, and amino acid metabolism, establishes it as a pivotal factor in the metabolic reprogramming of cancer cells 11. Targeting FGF19 could not only inhibit tumor growth but also disrupt the metabolic advantages that cancer cells gain through the Warburg effect and other metabolic adaptations. Moreover, the growing understanding of FGF19's role in metabolic reprogramming indicates potential synergies between cancer treatment and metabolic interventions. For example, leveraging the effects of FGF19 on energy expenditure and insulin sensitivity could provide new strategies for addressing cancer cachexia or metabolic comorbidities in cancer patients 319, 320. Future research should focus on elucidating the specific metabolic pathways regulated by FGF19 in different cancer types and identifying potential metabolic vulnerabilities that might be exploited for therapeutic benefit.

Additionally, the development of more specific and effective inhibitors, along with the identification of predictive biomarkers for response to FGF19/FGFR4-targeted therapies, is expected to improve clinical outcomes. Combination strategies that integrate FGFR4 inhibitors with immunotherapies, chemotherapy, or other targeted agents are considered potentially beneficial for overcoming resistance and enhancing patient survival 12, 246, 293, 321. The gut microbiota, bile acids, and FGF19 signaling pathway interact significantly, presenting a promising area for future cancer research. Modulating these elements through FGF19 may provide novel therapeutic strategies. The development of advanced drug delivery systems, including nanomedicine, can improve the precision and efficacy of FGF19 inhibitors. Nanoparticles can be engineered to deliver these inhibitors directly to tumor cells, reducing systemic toxicity and enhancing therapeutic outcomes. Furthermore, the potential for FGF19 as a biomarker in early cancer detection and monitoring warrants further investigation, given its involvement in multiple cancer types. As our understanding of FGF19's role in cancer continues to expand, the potential for innovative therapeutic approaches that capitalize on this knowledge is likely to increase, thereby enhancing the effectiveness of combating malignancies. Ultimately, the goal remains translating these insights into tangible improvements in cancer treatment and patient care. A multidisciplinary effort will be required to combine basic research, translational studies, and clinical trials, fully realizing the potential of targeting FGF19 in cancer therapy. With continued advancements, more effective and personalized treatment options for patients with FGF19-driven malignancies hold promise for the future.

## Figures and Tables

**Table 1:** The role of FGF family in cancer development

Member	Type of Cancer	Up (↑) / Down (↓) regulation	Model	Effects	Reference
FGF1	BC	↑	Animal Model *in vivo*: female Rag1KO mice, Cell Model *in vitro*: MCF7, TAMR	Promoting cancer progression and glycolytic phenotype, regulating metabolic reprogramming	322
	LC	↑	Cell Model *in vitro*: A549	Enriching and expanding liver cancer stem cells	323
	AS	\	Cell Model *in vitro*: ISOS-1	Inhibiting proliferation, invasion, and migration	324
	OC	↑	Human Model *in vivo*: OC patient samples, Animal Model *in vivo*: 7-8 week-old female nude mice, Cell Model *in vitro*: OVCAR3, OVCAR5, SKOV3, TOV-112D, TOV-21G, OV-90, MDAH 2774, ES2	Promoting tumor progression	325
	TC	↑	Human Model *in vivo*: TC and paracancerous tissues, Cell Model: B-CPAP, CAL-62	Promoting tumor invasion and migration	326
	MM	↑	Human Model *in vivo*: MM biopsy samples	Promoting melanoma pathogenesis	327
	PCa	↑	Animal Model *in vivo*: xenograft mouse, Cell Model *in vitro*: LNCaP, PC3, 22RV1, C4-2	Promoting tumor metabolic reprogramming	328
	BT	↑	Human Model *in vivo*: BT tissue samples, Cell Model *in vitro*: JMSU1, UMUC3	Promoting tumor proliferation	329
	NPC	↑	Human Model *in vivo*: nude mouse xenograft tumor model, Animal Model *in vivo*: nude mouse xenograft tumor model, Cell Model *in vitro*: SUNE1, CNE1, CNE2, 5-8F, 6-10B, HONE1	Promoting tumor growth and metastasis	330
FGF2	NPC	↑	Huma Model *in vivo*: NPC patient samples, Animal Model *in vivo*: female C57BL/6 and BALB/c-nude mice, Cell Model *in vitro*: 5-8F, SUNE-1	Promoting tumor metastasis	331
	OS	↑	Human Model *in vivo*: Human osteosarcoma sections, Cell Model *in vitro*: MG63, SaOS-2	Promoting tumor metastasis	332
	BC	↑	Cell Model *in vitro*: MCF10DCIS, MCF7, HS578T	Promoting cell migration and invasion	333
FGF3	BC	↑	Cell Model *in vitro*: MDA-MB-231, T47D, Cos-7	Accelerating cell cycle progression, promoting cell proliferation, and inhibiting cell apoptosis	334
	NSCLC	↑	Human Model *in vivo*: tissue samples from NSCLC patients, Animal Model *in vivo*: female BALB/c nu/nu mice, Cell Model *in vitro*: PC-9, HCC827	Promoting cell proliferation and gefitinib resistance	335
FGF4	CC	↓	Cell Model *in vitro*: SiHa, C-33A, ME-180, MS-751, HCC-94, HeLa	Inhibiting cell proliferation and metastasis	336
	BC	↑	Cell Model *in vitro*: MDA-MB-231, MCF-7	Promoting cell proliferation and metastasis	337
FGF5	NPC	↑	Animal Model *in vivo*: Male BALB/c nude mice, Cell Model *in vitro*: NPC/HK1, C666-1	Inhibiting ferroptosis, reducing cell sensitivity to cisplatin	338
	PanCa	↑	Human Model *in vivo*: patient samples, Cell Model *in vitro*: COLO-357	Promoting tumor proliferation	339
	OS	↑	Human Model *in vivo*: 15 OS patient samples, Animal Model *in vivo*: nude mouse orthotopic, Cell Model *in vitro*: U2OS, SAOS, MG63	Promotes cell proliferation	340
FGF6	OSCC	↑	Human Model *in vivo*: tissue samples of patients, Animal Model *in vivo*: male nude mice, Cell Model *in vitro*: HSC-4	Promoting cell proliferation, inhibiting apoptosis, and accelerating cell cycle	341
	BICa	↑	Cell Model *in vitro*: HUVECs	Promoting aerobic glycolysis and angiogenesis	342
FGF7	CCA	↑	Human Model *in vivo*: CCA patient samples, Cell Model *in vitro*: HuCCT1, RBE, CCLP-1, HCCC-9810	Promoting tumor cell proliferation, migration and invasion	343
	GC	↑	Human Model *in vivo*: GC patient samples, Cell Model *in vitro*: SGC7901, MKN28, NCI-N87	Promoting tumor invasion and migration	344
	OC	↑	Human Model *in vivo*: OC tissue specimens, Cell Model *in vitro*: A2780, HO8910	Promoting cancer progression, facilitating EMT	345
	PCa	↑	Animal Model *in vivo*: male athymic nude mice, Cell Model *in vitro*: PNT1A	Promoting tumor progression and invasion, enhancing cell proliferation	346
FGF8	EOC	↑	Human Model *in vivo*: EOC patient samples, Cell Model *in vitro*: SKOV3	Promoting tumorigenesis and metastasis	347
	HB	↑	Human Model *in vivo*: 35 childhood liver tumor samples; Cell Model *in vitro*: HUH6, HepG2 HepT1	Promoting tumor migration and invasion	348
FGF9	HCC	↑	Human Model *in vivo*: HCC patient samples; Cell Model *in vitro*: Hep3B, HepG2, PLC, Huh7	Promoting cell proliferation, enhancing clonogenic ability, migration capacity, and resistance to sorafenib	349
	TNBC	↑	Human Model *in vivo*: TNBC patient samples, Animal Model *in vivo*: Nude mice, Cell Model *in vitro*: MDA-MB-231, BT-549	Promoting cell proliferation, migration, invasion, and tumor growth	350
	LC	↑	Animal Model *in vitro* and vivo: mouse LLC cell line and six-week-old male C57BL/6 mice	Promoting tumorigenesis and tumor metastasis	351
	GC	↑	Human Model *in vivo*: 160 GC patient samples, Cell Model *in vitro*: MGC-803, SGC-7901	Promoting cell migration and invasion	352
FGF10	PanCa	↑	Human Model *in vivo*: 76 PanCa patient samples, Cell Model *in vitro*: AsPC-1, MIA PaCa-2, PANC-1, CFPAC-1	Promoting cell migration and invasion	353
FGF11	NSCLC	↑	Animal tumor xenograft model *in vivo*: Balb/c nude mice, Human Model *in vivo*: NSCLC patient samples, Cell Model *in vitro*: A549, NCI-H460, CALU3, H1975	Promote tumor cell proliferation, migration, and invasion	354
	OPSCC	↑	Human Model *in vivo*: tumor tissues with HPV+ TSCC, BOTSCC	Promote cell proliferation, migration, and invasion	355
	NPC	↓	Human Model *in vivo*: NPC patients' serum samples, Cell Model *in vitro*: NP69	Promoting tumor immune evasion	356
FGF12	ESCC	↑	Human Model *in vivo*: ESCC patient samples	Promote tumor cell proliferation, colony formation, and cell migration.	357
FGF13	CRC	↑	Human Model *in vivo*: 28 CRC patient samples, Cell Model *in vitro*: SW480, SW620, HT-29, HCT116, LOVO	Promoting cell proliferation, migration, and invasion	358
	BC	↑	Animal Model *in vivo*: mice, Cell Model *in vitro*: MMTV-PyMT 419	Promoting cell metastasis and migration	359
	TNBC	↑	Animal Model *in vivo*: mouse, Cell Model *in vitro*: MDA-MB-23, MCF-7, MDA-MB-361	Promoting cell metastasis	360
	HCC	↑	Cell Model *in vitro*: A549	Accelerating cell cycle progression, promoting proliferation, and inhibiting apoptosis	361
FGF14	BC	↓	Human Model *in vivo*: 45 BC patient samples, Animal Model *in vivo*: Female athymic BALB/c nu/nu mice, Cell Model *in vitro*: MCF-7, MDA-MB-453, MDA-MB-231, HCC-1937	Promoting tumor invasion and metastasis	362
	CRC	↓	Human Model *in vivo*: 13 CRC patient samples, Animal Model *in vivo*: 4-week-old male Balb/c nude mice, Cell Model *in vitro*: CaCO2, CL4, DLD-1, HCT116, HT29, LOVO, LS180, SW480, SW620, SW1116	Inhibiting cell proliferation, inducing apoptosis	363
FGF15 /FGF19	CRC	↑	Animal Model *in vivo*: male 6-week-old BALB/c nude mice, Cell Model *in vitro*: SW480, DiFi, Caco-2, DLD-1, SW620, LoVo, HT29, HCT116	Promoting tumor occurrence, progression, migration, and liver metastasis	183
	HCC	↑	Human Model *in vivo*: HCC patient samples, Animal Model *in vivo*: C57BL/6 mice, Cell Model *in vitro*: PLC/PRF/5, MHCC97H, Hepa1-6, H22	Promoting cell metastasis, migration, proliferation, and inhibiting apoptosis	154, 163
	PDAC	↑	Animal Model in vivo: KPC model mice and 10-week-old female nude mice, Cell Model *in vitro*: E3LZ10.7 (1), MIA PaCa-2, AsPC-1	Promoting cell proliferation, invasion, and metastasis	21, 37
	OC	↑	Human Model *in vivo*: HGSOC patient samples, Cell Model *in vitro*: OVCAR3, HO8910, HO8910pm, SKOV3, SKOV3-IP, A2780	Promoting autophagy and chemoresistance	18
	HNSCC	↑	Human Model *in vivo*: HNSCC patient samples, Animal Model *in vivo*: six-week-old NSG mice, Cell Model *in vitro*: HN6, HN12, HN30	Promoting tumor metastasis, reversing melatonin's suppressive effects on cancer progression	20
	BC	↑	Human Model *in vivo*: BC patient samples, Animal Model *in vivo* nude mice, Cell Model *in vitro*: MDA-MB-231	Promoting cell migration and invasion	16
	GC	↑	Human Model *in vivo*: 116 patient samples, Cell Model *in vitro*: MKN-28, MKN-45, SGC-7901, AGS	Promoting tumor migration and invasion	19
	CCA	↑	Animal Model *in vivo*: 6 week-old BALB/c nude mice, Cell Model *in vitro*: HCCC-9810, HuCCT1	Promoting cell proliferation, migration, and invasion	229, 230
	TC	↑	Human Model *in vivo*: TC patient samples, Cell, Model *in vitro*: B-CPAP, TCP-1	Promoting migration and invasion	22
	NPC	↑	Human Model *in vivo*: NPC patient samples, Animal model *in vivo*: 5-week-old BALB/c male nude mice, Cell Model *in vitro*: CNE1, CNE2, 5-8F, 6-10B, C666-1	Promoting tumor angiogenesis	13
	LUSC	↑	Human Model *in vivo*: LUSC patient samples, Animal Model *in vivo*: BALB/C nude mice, Cell Model *in vitro*: H520, SK-MES-1, HCC95, H1703	Promote cell proliferation	234
	GBC	↑	Human Model *in vivo*: GBC patient samples, Animal model *in vivo*: GPBAR1^-/-^ mice, Cell Model *in vitro*: GBC-SD	Promoting tumor growth and metastasis	162
FGF16	LC	↑	Human Model *in vivo*: LC patient samples, Cell Model *in vitro*: A549, H1299	Promote cell proliferation, influencing the tumor microenvironment	364
FGF17	PCa	↑	Human Model *in vivo*: PCa patient samples, Cell Model *in vitro*: LNCaP, DU145, PC3M	Promote cell proliferation, metastasis and carcinogenesis	365
FGF18	BC	↑	Cell Model *in vivo*: MCF-7, MDA-MB-453, SK-BR-3, T47D	Promoting cell migration and EMT	366
	OC	↑	Animal model *in vivo*: five female SCID hairless mice, Cell Model *in vitro*: A224, OVCA429, SKOV3	Promoting tumor cell invasion, metastasis and angiogenesis	367
	ccRCC	↓	Human Model *in vivo*: tumor patient samples, Animal Model *in vivo*: female BALB/c nude mice, Cell Model *in vitro*: 769-P, A498, 786-O	Inhibiting cell proliferation, invasion, and metastasis	368
FGF20	Glioma	↑	Cell Model *in vitro*: U251	Promoting tumor immune evasion, proliferation, invasion, and angiogenesis	369
FGF21	BC	↑	Human Model *in vivo*: BC patient samples, Animal Model *in vivo*: hemizygous female MMTV-PyMT mice, Cell Model *in vitro*: MDA-MB-231, BT-549, E0771, MCF-7, 4T1, MIHA, MCF-10A	Promoting tumor growth, inhibiting apoptosis	370
	NSCLC	↑	Human Model *in vivo*: 34 NSCLC patient samples, Cell Model *in vitro*: A549, H460	Promoting cell growth and migration	371
	PCa	↓	Human Model *in vivo*: NSCLC patient samples, Animal Model *in vivo*: 5-week-old male BALB/c nude mice, Cell Model *in vitro*: LNCaP, 22Rv1, DU145, PC3, RWPE-1	Inhibiting cell proliferation, promoting apoptosis	372
FGF22	PanCa	↑	Human Model *in vivo*: PanCa patient samples, Cell Model *in vitro*: PANC-1, Mia PaCa-2	Promoting cell migration and invasion	373
FGF23	OS	↑	Cell Model *in vitro*: hFOB1.19, MG-63, U2-OS	Promoting cell proliferation, migration, and invasion	374

AS, angiosarcoma; BC, breast cancer; BICa, bladder tumor; ccRCC, clear cell renal cell carcinoma; CC, cervical cancer; CRC, colorectal cancer; CCA, cholangiocarcinoma; EMT: epithelial-mesenchymal transition; ESCC, esophageal squamous cell carcinoma; EOS, epithelial ovarian cancer; GBC, gallbladder cancer; GC, gastric cancer; HNSCC, head and neck squamous cell carcinoma; HCC, hepatocellular carcinoma; HB, hepatoblastoma; HGSOC, high-grade serous ovarian cancer; LLC: lewis lung carcinoma; LUSC, lung squamous cell carcinoma; LC, lung cancer; MM, melanoma; NPC, nasopharyngeal carcinoma; NSCLC, non-small cell lung cancer; OC, ovarian cancer; OSCC, oral squamous cell carcinoma; OS, osteosarcoma; PDAC, pancreatic ductal adenocarcinomas; PanCa, pancreatic cancer; PCa, prostate cancer; TC, thyroid carcinoma; TNBC, triple negative breast cancer.

**Table 2:** The inhibitors of FGF19/FGFR4-targeted therapeutic strategies

Trial Design	Inhibitor	Institute/Company	Structure	Molecular Formula	Phase/ Clinical trial ID	Reference
ALC, HCC	Irpagratinib / ABSK011	Abbisko Therapeutics Co., Ltd.	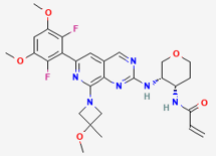	C₂₈H₃₂F₂N₆O₅	Phase 1 / NCT04906434, Phase 2 / NCT05441475	208
HCC	Roblitinib / FGF401	Novartis Pharma AG	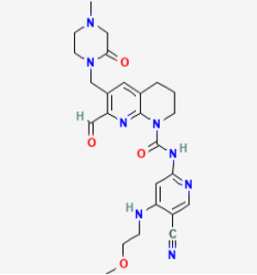	C_25_H_30_N_8_O_4_	Phase 1/2 / NCT02325739	246
	Fisogatinib / BLU-554	Blueprint Medicines	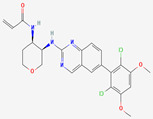	C_24_H_24_Cl_2_N_4_O_4_	Phase 1/2 / NCT04194801, phase 1 / NCT02508467	12, 249
	H3B-6527	H3 Biomedicine, Inc.	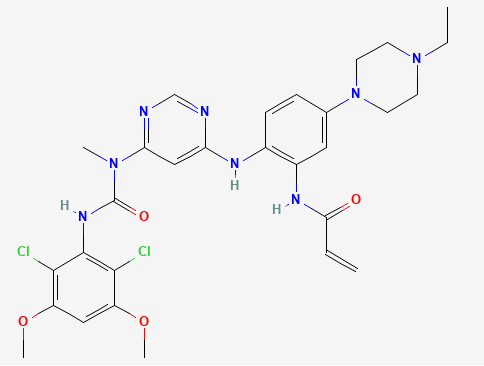	C_29_H_34_Cl_2_N_8_O_4_	Phase 1 / NCT02834780	251
NSCLC, EC, CCA, BT, PCa, aUC, HCC	Erdafitinib	Astex Pharmaceuticals, Inc.	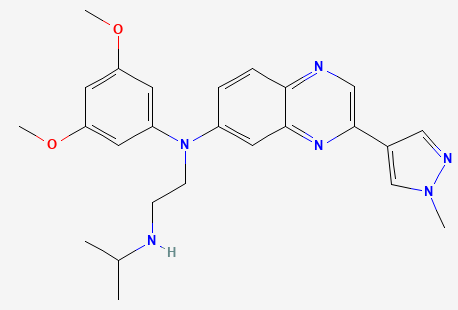	C_25_H_30_N_6_O_2_	Phase 1 / NCT03238196, phase 1 / NCT03547037, phase 2 / NCT02699606, phase 2 / NCT04172675, phase 1/2 / NCT02421185	257, 261
ICC, BC, AST, CCA	Futibatinib / TAS-120	Taiho Pharmaceutical Co., Ltd.	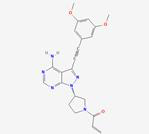	C_22_H_22_N_6_O_3_	Phase 1/2 / NCT02052778, phase 2 / NCT04024436, phase 2/3 / NCT06506955	263, 264
NSCLC, MG, mUC, GC, BC, advanced tumors	AZD4547	AstraZeneca	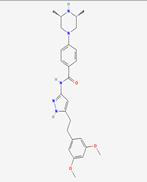	C_26_H_33_N_5_O_3_	Phase 1 / NCT00979134, phase 1/2 / NCT01824901, phase 1/2 / NCT02824133, phase 1/2 / NCT01202591, phase 1 / NCT01213160, phase 2 / NCT01457846	375
AST	Derazantinib / ARQ0873	ArQule, Inc	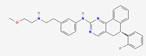	C_29_H_29_FN_4_O	Phase 1/2 / NCT01752920	376
ST, CCA, UC	Pemigatinib	Incyte Biosciences Distribution BV	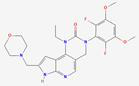	C_24_H_27_F_2_N_5_O_4_	Phase 2 / NCT04256980, phase 2 / NCT02872714, phase 2 / NCT05565794	377, 378
aUC, SQCLC, BC	Rogaratinib	Bayer AG	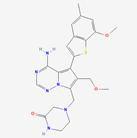	C_23_H_26_N_6_O_3_S	Phase 1, NCT03473756, phase 1 / NCT04483505, phase 2 / NCT03762122, phase 2 / NCT04040725	379
PanCa	BLU-9931	Blueprint Medicines	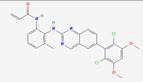	C_26_H_22_Cl_2_N_4_O_3_	Preclinical	256

ALC, advanced liver cancer; AST, advanced solid tumors; aUC, advanced urothelial carcinoma; BC, breast cancer; BT, bladder tumor; CCA, cholangiocarcinoma; EC, esophageal cancer; GC, gastric cancer; HCC, hepatocellular carcinoma; iCC, intrahepatic cholangiocarcinoma; mUC, metastatic urothelial carcinoma; MG, malignant glioma; NSCLC, non-small cell lung cancer; PCa, prostate cancer; PanCa, pancreatic cancer; ST, solid tumor; SQCLC, squamous-cell lung carcinoma; UC, urothelial carcinoma.

## Abbreviations

ANXA2: annexin A2
ADCs: antibody-drug conjugates
ATF4: activating transcription factor 4
ACC: acetyl-coA carboxylase
AEs: adverse events
BC: breast cancer
CD: Crohn's disease
CAFs: cancer-associated fibroblasts
CYP7A1: cholesterol 7α-hydroxylase
CRC: colorectal cancer
CAR: chimeric antigen receptor
CCA: cholangiocarcinoma
CDKs: cyclin-dependent kinases
DFSP: dermatofibrosarcoma protuberans
DLTs: dose-limiting toxicities
DCA: deoxycholic acid
DCR: disease control rate
DOR: duration of response
EOC: epithelial ovarian cancer
ER: endoplasmic reticulum
EGFR: epidermal growth factor receptor
EMT: epithelial-mesenchymal transition
FASN: fatty acid synthase
FGF: fibroblast growth factor
FGF19: fibroblast growth factor 19
FGFR4: fibroblast growth factor receptor 4
FXR: farnesoid X receptor
GEAC: gastroesophageal adenocarcinoma
GTAT4: GATA binding protein 4
GC: gastric cancer
GBM: glioblastoma multiforme
HMGA1: high mobility group A1
HCC: hepatocellular carcinoma
HNSCC: head and neck squamous cell carcinoma
iCAFs: inflammatory cancer-associated fibroblasts
iCCA: intrahepatic cholangiocarcinoma
KLB: β-Klotho
KLF15: Krüppel-like factor 15
LIF: leukemia inhibitory factor
LSQ: lung squamous cell carcinoma
LUSC: lung squamous cell carcinoma
mTORC1: mechanistic target of rapamycin complex 1
mUC: metastatic urothelial carcinoma
mAbs: monoclonal antibodies
MDSCs: myeloid-derived suppressor cells
MAFLD: metabolic-associated fatty liver disease
MMAE: monomethyl auristatin E
MMPs: matrix metalloproteinases
NET: neutrophil extracellular trap
NPC: nasopharyngeal carcinoma
NASH: non-alcoholic steatohepatitis
OS: overall survival
OCA: obeticholic acid
ORR: overall response rate
PDO: patient-derived organoids
PBC: primary biliary cholangitis
PD-L1: programmed death-ligand 1
PD-1: programmed cell death protein 1
PSA: prostate-specific antigen
PCa: prostate cancer
PIP2: phosphorylates phosphatidylinositol-4,5-bisphosphate
PIP3: phosphatidylinositol-3,4,5-trisphosphate
PFS: progression-free survival
PDX: patient-derived xenograft
PDK1: pyruvate dehydrogenase kinase 1
PDH: pyruvate dehydrogenase
PDAC: pancreatic ductal adenocarcinoma
QD: quaque die
RMS: rhabdomyosarcoma
SOCE: store-operated calcium entry
SHP: small heterodimer partner
siRNA: small interfering RNA
shRNA: small hairpin RNA
TC: thyroid cancer
TCA: tricarboxylic acid
TAMs: tumor-associated macrophages
TRIM21: tripartite motif containing 21
TNM: tumor, node, metastasis
TRAE: treatment-related adverse event
TGF-β: transforming growth factor-β
VEGF: vascular endothelial growth factor
